# Bone-associated gene evolution and the origin of flight in birds

**DOI:** 10.1186/s12864-016-2681-7

**Published:** 2016-05-18

**Authors:** João Paulo Machado, Warren E. Johnson, M. Thomas P. Gilbert, Guojie Zhang, Erich D. Jarvis, Stephen J. O’Brien, Agostinho Antunes

**Affiliations:** CIIMAR/CIMAR, Interdisciplinary Centre of Marine and Environmental Research, University of Porto, Rua dos Bragas, 177, 4050-123 Porto, Portugal; Abel Salazar Biomedical Sciences Institute (ICBAS), University of Porto, Porto, Portugal; Smithsonian Conservation Biology Institute, National Zoological Park, 1500 Remount Road, Front Royal, VA 22630 USA; Centre for GeoGenetics, Natural History Museum of Denmark, University of Copenhagen, Øster Volgade 5-7, 1350 Copenhagen, Denmark; China National GeneBank, BGI-Shenzhen, Shenzen, 518083 China; Centre for Social Evolution, Department of Biology, Universitetsparken 15, University of Copenhagen, DK-2100 Copenhagen, Denmark; Department of Neurobiology Box 3209, Duke University Medical Center, Durham, NC 27710 USA; Howard Hughes Medical Institute, Chevy Chase, MD 20815 USA; Theodosius Dobzhansky Center for Genome Bioinformatics, St. Petersburg State University, St. Petersburg, 199004 Russia; Oceanographic Center, 8000 N. Ocean Drive, Nova Southeastern University, Ft Lauderdale, FL 33004 USA; Department of Biology, Faculty of Sciences, University of Porto, Porto, Portugal

## Abstract

**Background:**

Bones have been subjected to considerable selective pressure throughout vertebrate evolution, such as occurred during the adaptations associated with the development of powered flight. Powered flight evolved independently in two extant clades of vertebrates, birds and bats. While this trait provided advantages such as in aerial foraging habits, escape from predators or long-distance travels, it also imposed great challenges, namely in the bone structure.

**Results:**

We performed comparative genomic analyses of 89 bone-associated genes from 47 avian genomes (including 45 new), 39 mammalian, and 20 reptilian genomes, and demonstrate that birds, after correcting for multiple testing, have an almost two-fold increase in the number of bone-associated genes with evidence of positive selection (~52.8 %) compared with mammals (~30.3 %). Most of the positive-selected genes in birds are linked with bone regulation and remodeling and thirteen have been linked with functional pathways relevant to powered flight, including bone metabolism, bone fusion, muscle development and hyperglycemia levels. Genes encoding proteins involved in bone resorption, such as *TPP1*, had a high number of sites under Darwinian selection in birds.

**Conclusions:**

Patterns of positive selection observed in bird ossification genes suggest that there was a period of intense selective pressure to improve flight efficiency that was closely linked with constraints on body size.

**Electronic supplementary material:**

The online version of this article (doi:10.1186/s12864-016-2681-7) contains supplementary material, which is available to authorized users.

## Background

**Fig. 1:**
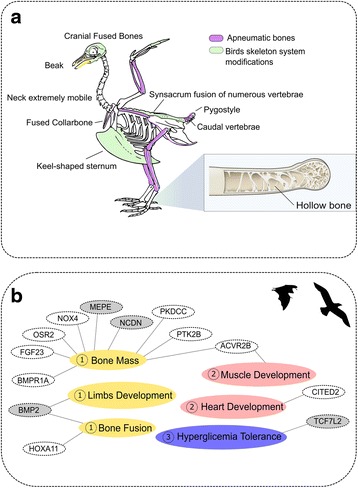
Skeleton adaptations in birds and mammals and adaptive selection in bone-associated genes. **a** Rock pigeon skeleton (adapted from Wikimedia Commons licensed under a Creative Commons Attribution-Share Alike 3.0 Unported (CC BY-SA 3.0)) showing the key bone modifications observed in birds, and bones containing red-blood-cell-producing marrow (apneumatic bones). Most bones (except very small ones) are pneumatized. The structure of a pneumatic bone is highlighted in the light blue box (licensed by Rice University under a Creative Commons Attribution License (CC-BY 3.0)). **b** Positively selected genes in birds and those genes showing a dissimilar evolutionary rate in bats when compared to other mammals (lower evolutionary rate—colored in grey; and higher evolutionary rate—colored in white). Representation of the link between gene and physiological/development systems (colored accordingly: skeleton system (1), muscular system (2) and glucose (3) that are plausibly related with flight adaptation

Powered flight evolved independently in birds and bats, but required similar trade-offs and limitations, including strong constraints on traits such body size [1, 2] and skeletal structure to minimize energy requirements [3]. While body sizes have tended to increase through evolutionary time in many lineages [4], the size of flying vertebrates has been more constrained [5]. However, postcranial skeleton pneumatization (hollow air-filled bones) and bone modifications (such as bone fusion) may have provided increased evolutionary flexibility among birds [6] (Fig. 1a). In birds, hollow bones are formed with pneumatic foramina or openings in the wall of the bone that permit air sacs to perforate internal bone cavities [7, 8]. The development of pneumatic bones in birds led to reductions in overall body mass and has also been associated with bone resorption [6, 9]. These pneumatic bones have often been assumed to have lightened the entire avian skeleton relative to mammals [10] and to have reduced the metabolic cost of flight [3, 11–14]. However, some skeletal structures, such as the humerus, ulna-radius, tibio-tarsus and fibula, have more body mass in birds than mammals [15], suggesting that modern bird skeletons have experienced diverse bone-specific selection patterns.

**Fig. 2:**
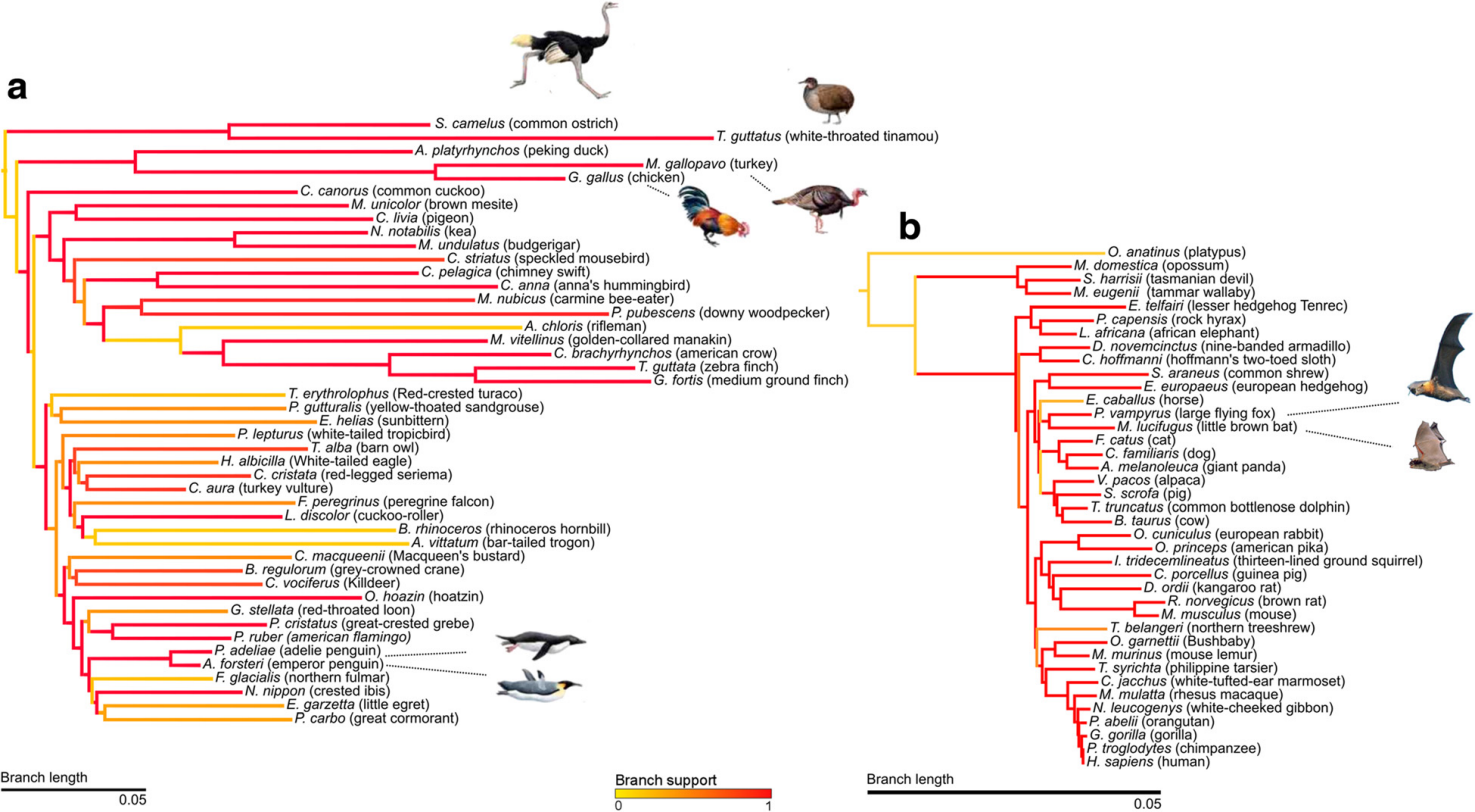
The gene-tree-based phylogeny from concatenation analysis of 89 genes in 45 avian and 39 mammalian genomes using maximum likelihood. **a** The species with images are flightless. The species *Haliaeetus leucocephalus* (Bald Eagle) and *Pelecanus crispus* (Dalmatian Pelican) were excluded from the phylogenetic analyses given the low number of retrieved sequences (n < =5). **b** The species with images represent the species with powered flight

Bats are the only mammals capable of sustained flight, but have distinct traits than birds that likely reflect key differences in ecological adaptations and distinct evolutionary histories [16]. Bats have elongated fingers instead of elongated forearms as seen in birds and have bones with high levels of mineral density that increases the stiffness of the skeleton [3]. On the other hand, as with birds, bats have relatively small bodies [17], fused bones and lightweight skeletons [3] (Additional file 1: Figure S1). Many of the other shared traits among birds and bats are probably also associated with the challenges imposed by the evolution of powered flight (Additional file 1: Figure S1). These include improved respiratory systems [18], high metabolic output [19], hyperglycemia tolerance [20, 21], diminished production of reactive oxidative species [22, 23] and smaller intestines [24].

Here, we tested the evolutionary rate of change in 89 bone-associated genes in 47 avian and 39 mammalian genomes and evaluated genetic distinctions among flying versus non-flying species to assess patterns of selection in genes involved in bone development. Birds displayed a higher number of the bone-associated genes under positive selection, the majority of which were associated with regulatory process of bone remodeling. Of the 89 analyzed genes, 13 positively-selected genes in birds also had different evolutionary rates in bats relative to other mammals. These were mainly genes involved in bone fusion and bone-remodeling, which affirms the role of adaptive selection as a key process driving the evolution of flight.

## Results

### Bone-associated gene locations and related phylogenetic analyses

The 89 bone-related genes (Additional file 2: Table S1) represent a subset of the genes associated with bone development [25]. These bone-associated genes were distributed widely across the genomes of mammals and birds (Additional file 3: Figure S2).

The inferred topology for bone-associated genes was significantly different from the avian species tree using the whole genome data [26, 27], ∆*lnL* = 1891.34, but more similar to the tree topology obtained from protein coding only genes [27] ∆*lnL* = 537.06 (Fig. 2a). Both the avian species-tree and protein coding-genes tree showed significant differences under the tests 1sKH (one sided KH test based on pairwise SH tests), SH (Shimodaira-Hasegawa), and ELW (Expected Likelihood Weight) at a critical 5 % significance level relative to those obtained with the bone-associated gene-tree-based phylogeny. With the mammalian bone-associated genes the tree topology was slightly different from the mammalian species tree [28, 29], since significant differences were obtained under the tests 1sKH, SH, and ELW at 5 % significance level, ∆*lnL* = 271.70 (comparison accepted species tree vs. obtained tree) (Fig. 2b). We note that the mammalian species tree was also generated mostly with protein coding sequences.

### Site-models show a higher evolutionary rate in bird bone-associated genes

**Fig. 3:**
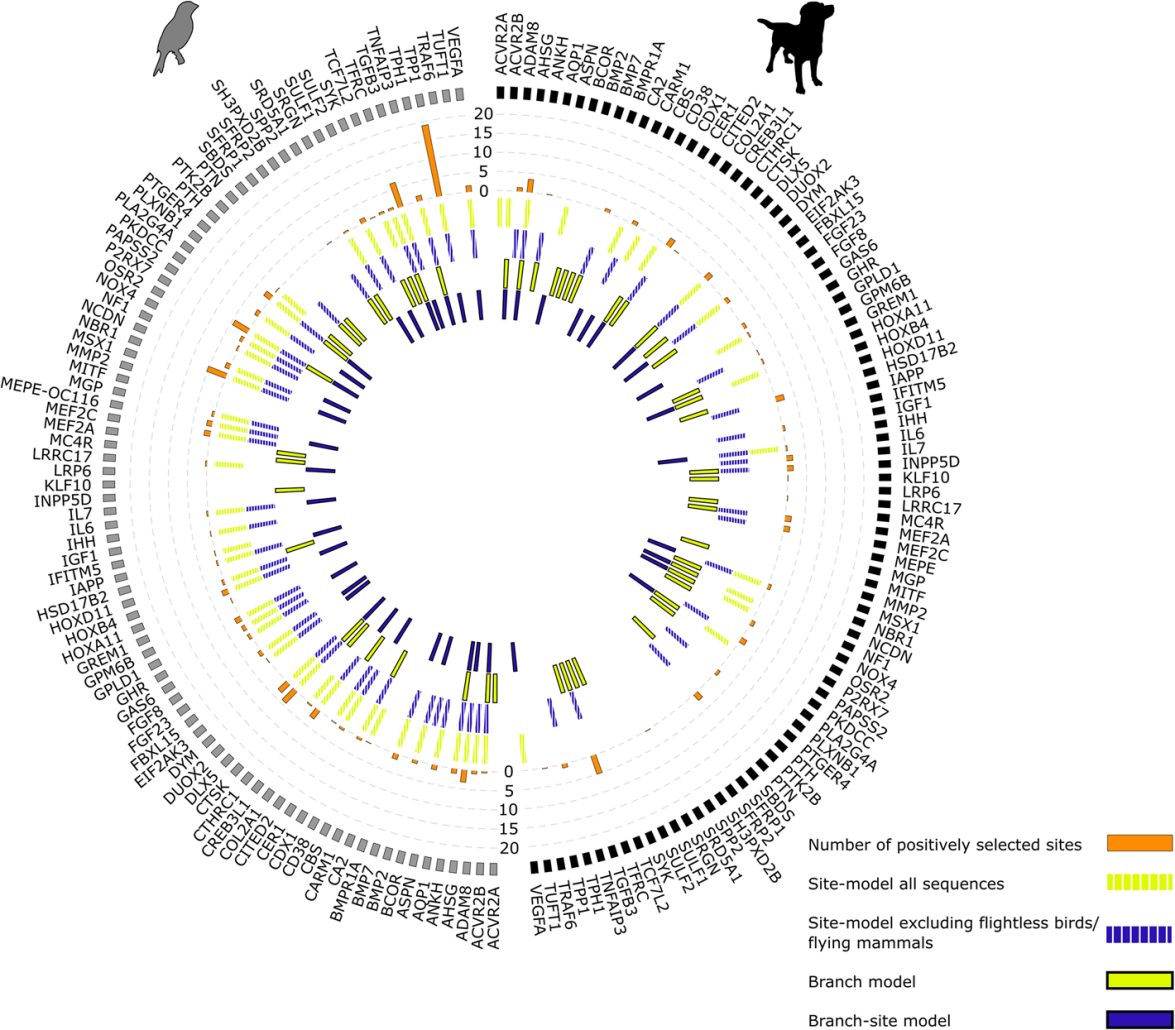
Positive selection in bird and mammal bone-associated genes. All results from evolutionary analyses were corrected for multiple testing using the q-value. The bars in the four inner circles show which of the alternate models (listed in the lower right corner) are most likely. The genes listed on the left of the circle are from the bird analyses and those on the right are the results for mammals. In the four inner circles, the presence of the bars represent positively selected genes after running the models M2a vs M1a. The bars closest to the gene names indicate the number of positively selected genes (posterior probabilities > = 0.95), each tick represents 5 positively selected sites under Bayesian Empirical Bays post-hoc analysis

**Fig. 4:**
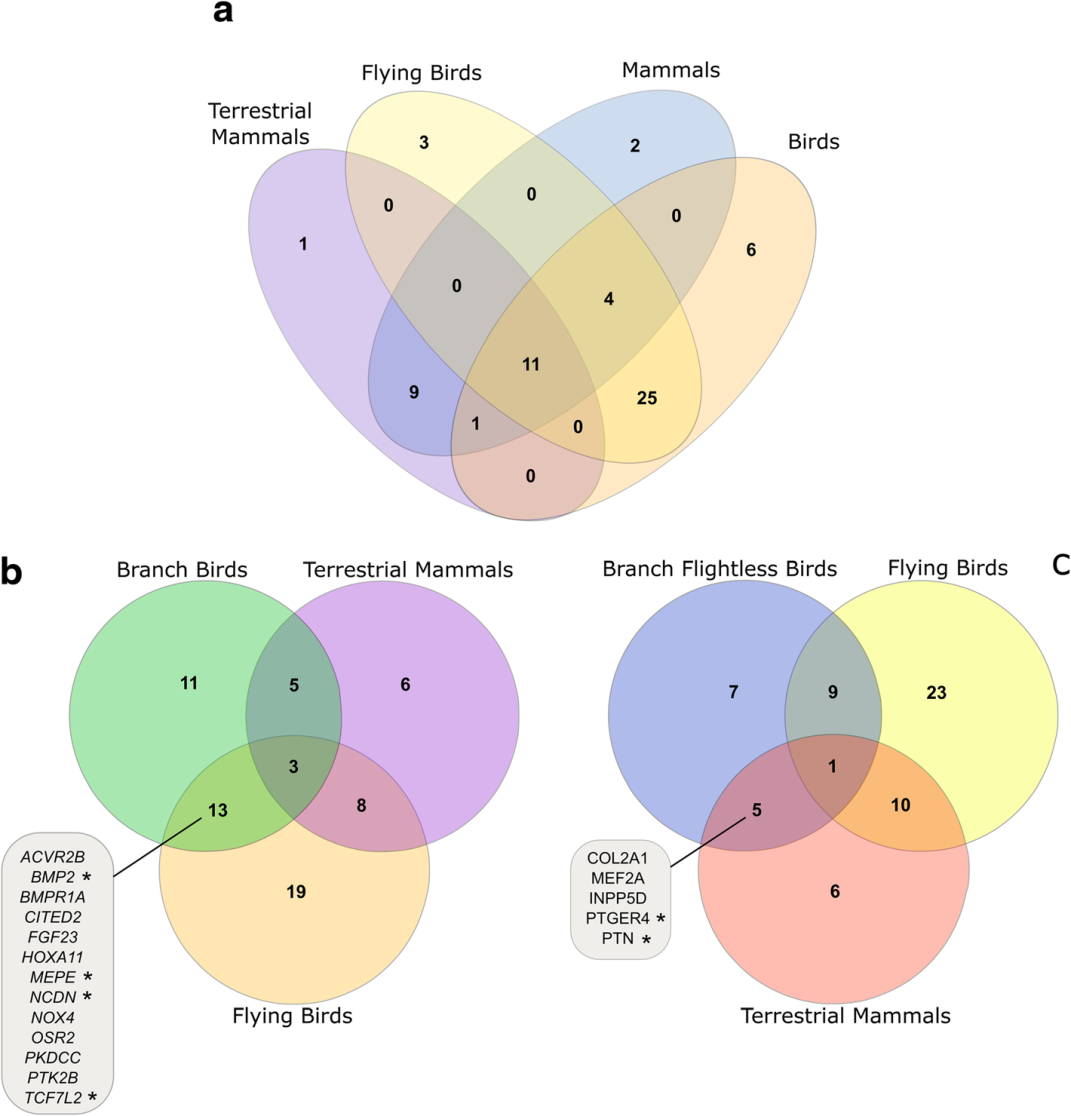
Venn diagrams of positively-selected bone-associated genes. **a** Intersection between positively-selected genes shared in different combinations among mammals and birds, with the datasets including only terrestrial mammals and flying birds. **b** Intersection between positively-selected genes in terrestrial mammals, flying birds and those genes showing a different evolutionary rate in bats. **c** Intersection between positively-selected genes in terrestrial mammals, branch of flightless birds and flying birds. Asterisks (*) represent genes where the foreground branch was slower than background

In site models, of the 89 mammalian genes, 27 (~30.3 %) favored the alternate model (evolved under positive selection) (Fig. 3; Additional file 4: Table S2), whereas in birds, 47 (52.8 %) were positively selected (Fig. 3; Additional file 5: Table S3). This difference in the number of selected genes in birds compared to mammals was significant (Fisher’s Exact Test, two-tailed, *p*-value = 0.003722). Additionally, we tested for signals of positive selection in reptiles. The observed positive selection in birds is a unique signature and not a ubiquitous tendency in sauropsida, since only 20 (~22 %) of 88 genes showed significant evidence of positive selection in reptiles (Additional file 6: Table S4). Furthermore, the presence of positive selection in bone-associated genes revealed different targets in the three different clades (Additional file 7: Figure S3). Of the 89 genes, ~18 % (16) were positively selected in both birds and mammals, 34.8 % (31) were only positively selected in birds and only 12.4 % (11) were identified in only mammals (Fig. 4a).

In birds the highest global omega values (0.53 and 0.71) were observed for *AHSG* (Alpha-2-HS-glycoprotein) and *P2RX7* (P2X purinoceptor 7), respectively (Additional file 5: Table S3). Both genes are associated with bone mineral density and bone remodeling [30, 31]. However, considering only the number of sites with omega > 1.0 and a Posterior Probability (pp) ≥ 0.95, two genes involved in bone resorption, *TPP1* (Tripeptidyl peptidase I) and *TFRC* (Transferrin Receptor), had the highest number of positively selected sites, 95 and 33, respectively, corresponding to 19.8 % and 4.2 % of the alignment length (Additional file 5: Table S3). Since tpp1 protein is secreted by osteoclasts and Peptidase S53 is involved in bone collagen proteolysis [32], the positive selection may be related with the optimization of this proteolytic process during bone resorption.

### Branch and branch-site models show increased selection in bone genes of flying species

For the branch-model analyses, the datasets were labeled according to their life-habits (flying vs. non-flying). Flightless birds [33] included those unable to sustain flight for long distances (such as turkey or chicken), aquatic-birds and running birds (e.g. ratites). This approach permitted the identification of genes evolving under different evolutionary rates in the different lineages of flightless and flying species. The correlation between mammals and birds had the lowest rho (ρ) value for flightless birds and flying mammals (Spearman’s ρ = 0.579; *p*-value < 0.01) (Table 1). The highest similarities in d_N_/d_S_ values were obtained within each taxonomic clade; for bats and other mammals ρ = 0.833 (*p*-value <0.01) and for flightless and flying birds ρ = 0.883 (*p*-value <0.01). These patterns suggest that although a relatively small number of sites were affected, they were sufficient to be identified as evolving under positive selection, yet were insufficient to result in a significant different evolutionary rates between flying and flightless species. This is particularly evident in the branch-site models, since 10 of 86 genes (three genes were unreported in chiropterans species) were best fit the alternate model in branch-site analyses in flying birds and bats (Additional file 8: Table S5 and Additional file 9: Table S6). While 52 out of 86 genes best fit the null model in both flying birds and bats, in bats 59 out 86 genes and 63 out of 86 genes in flying birds had at least one site with an pp > =0.5 (Additional file 8: Table S5 and Additional file 9: Table S6). This suggests that positive selection only affected a few sites while the majority of the proteins evolved under neutral and/or negative selection. Only 879 sites in flying birds (Additional file 8: Table S5) and 475 sites from a total of 53,526 analyzed positions were positively selected in flying mammals (Additional file 9: Table S6). The branch-site analyses also revealed four genes with the same positively-selected sites in both flying birds and bats, *AHSG* (two sites), *ANKH*, ANKH Inorganic Pyrophosphate Transport Regulator (one site), *HOXA11*, Homeobox protein Hox-A11, (three sites), *MC4R*, Melanocortin receptor 4 (one site).

**Table 1:** Spearman correlations between the estimated ω for branches: Flight vs Non-Flight Birds and Other Mammals vs Bats

	Flying Birds	Flightless Birds	Bats	Flightless Mammals
Flying Birds	-	0.883	0.605	0.717
Flightless Birds		-	0.579	0.668
Bats			-	0.833
Flightless Mammals				-

All correlations are significant at the *p* < 0.01 (2-tailed). The sample used for the correlation, list-wise *n* = 85

### Flying species have a high prevalence of positive selection in bone regulatory genes

**Fig. 5:**
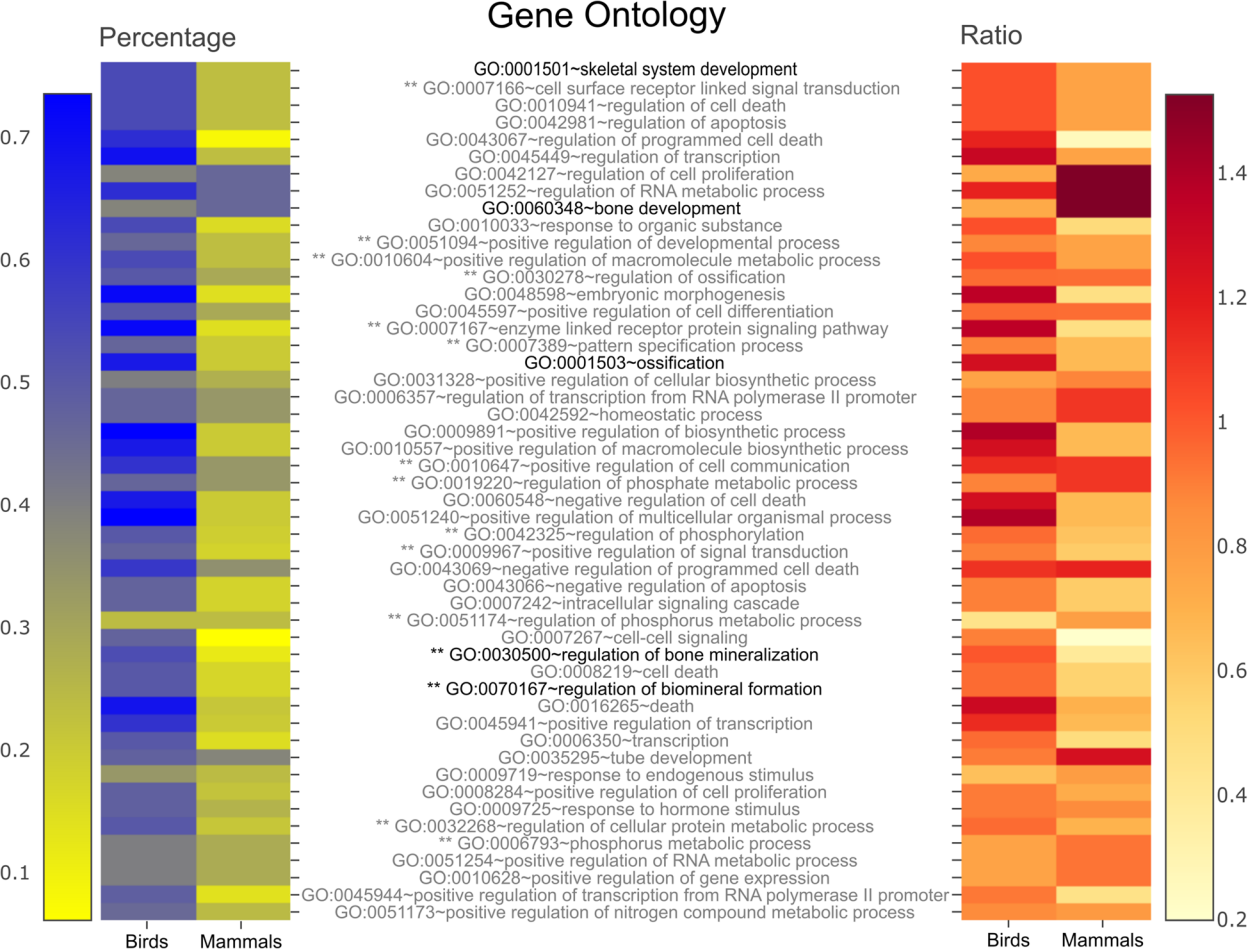
Functional annotation of positively-selected genes in birds and mammals. The heat map on the left represents the percentage of positively-selected genes in birds and mammals for each GO category. Terms directly associated with bones are highlighted in bold, and those where there is a significant statistical difference between birds and mammals, upon Fisher’s Exact Test, are marked with two asterisks (**). The heat map on the right presents the ratio obtained in heat map on the left for each GO term, divided by the ratio of positively-selected genes in birds and mammals respectively. A value great than one is indicative that there is evidence that the GO category has experienced positive selection

In birds, the functional category analysis showed that genes under positive selection are mainly involved in processes regulating ossification (13 out of 19, ~68 %), bone mineralization (10 out of 14, ~71 %) and biomineral formation (10 out of 14, ~71 %) (Fig. 5). These processes are significantly less represented in the list of positively-selected genes in mammals (Fisher’s Exact Test *p*-value < 0.01). Notably, 13 genes that were positively selected in birds also had different evolutionary rates between bats and non-flying mammals (Fig. 4b; Additional file 10: Table S7 and Additional file 11: Table S8). Additionally, we identified five genes that had different evolutionary rate in flightless birds and were positively selected in terrestrial mammals and negatively selected in flying birds (Fig. 4c; Additional file 12: Table S9 and Additional file 13: Table S10).

### Correlation between substitution rates and body mass

To determine if there is a possible correlation between evolution rates in flying species and body mass, we used the Bayesian method CoEvol that provides comparisons between rates of change in phenotypic traits and rates of molecular evolution [34]. In CoEvol, a high posterior-probability of covariance between the rate of change in d_S_, d_N_/d_S_, GC nucleotide content and the change of a phenotypic trait would suggest that there is evidence of a link between molecular and phenotypic processes. The separate estimation of covariance for d_S_ and d_N_/d_S_ distinguishes mutational effects of d_S_ from selective effects of d_N_/d_S._ In birds, high GC content has been associated with large population sizes and short generation times [35]. Therefore, GC content analysis can act as a control measure for the effects of small-bodied animals with putatively large populations that typically have lower the d_N_/d_S_ ratios [36]. Comparison between all birds vs. only flying birds was used to help understand the effect in the model estimation when flightless birds were included. A similar approach was employed for mammals, using a dataset including all mammals compared with other sets using only terrestrial mammals.

When only bird species that could fly were tested, a negative covariance was found between average body mass and d_S_ (R = −0.507, posterior probability (pp) =0.023**), GC content and d_N_/d_S_ (R = −0.9605, pp = 0**). When flightless species were included, in addition to the d_S_ correlation with body mass (average) (R = −0.398, pp = 0.039*), there was also a negative covariance between GC content and body mass (R = −0.542, pp = 0.0405*), and a positive correlation between d_N_/d_S_ and the body mass (R = 0.507, pp = 0.955*) (Table 2; Additional file 14: Figure S4).

**Table 2:** Covariance between d_S_, ω (d_N_/d_S_), gc content, and the three body mass measures (minimum, maximum and average) in 45 bird genomes

	Avian dataset					
	d_S_	ω	gc	Minimum weight	Maximum weight	Average weight
d_S_	-	−0.0358 (0.425)	0.07445 (0.655)	**−0.403** **(0.0355)** ^**b**^	**−0.3965** **(0.04)** ^**b**^	**−0.398** **(0.039)** ^**b**^
ω	−0.1645 (0.215)	-	**−0.9465** **(0.0014)** ^**a**^	**0.499** **(0.95)** ^**b**^	**0.5055** **(0.955)** ^**b**^	**0.507** **(0.955)** ^**b**^
gc	0.196 (0.83)	**−0.9605** **(0)** ^**a**^	-	**−0.534** **(0.0425)** ^**b**^	**−0.5405** **(0.0395)** ^**b**^	**−0.542** **(0.0405)** ^**b**^
Minimum weight	**−0.5005** **(0.024)** ^**a**^	0.132 (0.64)	−0.1475 (0.345)	-	**0.9935** **(1)** ^**a**^	**0.997** **(1)** ^**a**^
Maximum weight	**−0.506** **(0.0245)** ^**a**^	0.07725 (0.58)	−0.0976 (0.4)	**0.9895** **(1)** ^**a**^	-	**0.999** **(1)** ^**a**^
Average weight	**−0.507** **(0.023)** ^**a**^	0.0979 (0.605)	−0.1168 (0.38)	**0.995** **(1)** ^**a**^	**0.999** **(1)** ^**a**^	-

The upper triangle shows the values obtained for all birds and the lower triangle excluding flightless birds. Each cell represent the covariance values and posterior probability are the bracketed values, posterior probability (^a^ - < = 0.025 or > =0.975; ^b^ - < =0.05 or > =0.95) are highlighted in bold for the statistically significant correlations

Mammals exhibited a different trend, since when bats were included, there was a negative correlation between body mass and d_S_ (R = −0.534, pp = 0.0093**), and between body mass and GC content (R = −0.5035, pp = 0.01615**) and a positive correlation with body mass and d_N_/d_S_ (R = 0.496, pp = 0.985**) (Table 3). In contrast, when bats were excluded, d_N_/d_S_ (R = 0.572, pp = 0.995**), d_S_ (R = −0.5465, pp = 0.01085**) and GC (R = −0.511, pp = 0.012**) were significantly correlated with average body mass. Thus, in contrast with the results of birds’ analysis, the correlation between body size and d_N_/d_S_ was maintained, independent of including or excluding bats (flying species) in the mammalian dataset.

**Table 3:** Covariance between d_S_, ω (d_N_/d_S_), gc content, and the three weight measures (minimum, maximum and average) in 39 mammal genomes

	Mammalian dataset					
	d_S_	ω	gc	Minimum weight	Maximum weight	Average weight
d_S_	-	**−0.5265** **(0.014)** ^**a**^	**0.351** **(0.95)** ^**b**^	**−0.566** **(0.00715)** ^**a**^	**−0.522** **(0.0098)** ^**a**^	**−0.534** **(0.0093)** ^**a**^
ω	**−0.4825** **(0.0375)** ^**b**^	-	**−0.4635** **(0.0025)** ^**a**^	**0.5045** **(0.985)** ^**a**^	**0.4855** **(0.985)** ^**a**^	**0.496** **(0.985)** ^**a**^
gc	0.3395 (0.93)	**−0.4655** **(0.00605)** ^**a**^	-	**−0.4615** **(0.0295)** ^**b**^	**−0.4995** **(0.0185)** ^**a**^	**−0.5035** **(0.01615)** ^**a**^
Minimum weight	**−0.5705** **(0.0084)** ^**a**^	**0.569** **(0.995)** ^**a**^	**−0.455** **(0.027)** ^**b**^	-	**0.9635** **(1)** ^**a**^	**0.974** **(1)** ^**a**^
Maximum weight	**−0.535** **(0.0124)** ^**a**^	**0.562** **(0.995)** ^**a**^	**−0.5095** **(0.013)** ^**a**^	**0.96** **(1)** ^**a**^	-	**0.999** **(1)** ^**a**^
Average weight	**−0.5465** **(0.01085)** ^**a**^	**0.572** **(0.995)** ^**a**^	**−0.511** **(0.012)** ^**a**^	**0.9715** **(1)** ^**a**^	**0.998** **(1)** ^**a**^	-

The upper triangle shows the values obtained for all mammals and the lower triangle excluding bats. Each cell represent the covariance values and posterior probability are the bracketed values, posterior probability (^a^ - < = 0.025 or > =0.975; ^b^ - < =0.05 or > =0.95) are highlighted in bold for the statistically significant correlations

For mammals and birds the results were also consistent under a different phylogenetic assumption, *i.e.*, using the gene-based tree instead of the species tree (Additional file 15: Table S11 and Additional file 16: Table S12). These findings suggest that including or excluding bats has little effect on the results which can be partially explained by the relatively small number of bats in the dataset (~5 % of the total amount of sequences) compared with the larger percentage of flightless species (~87 %) in the avian comparison. Additionally, the large flying fox is often reported as the largest bat, and therefore potentially introduces a slight bias in the analyses given its large body mass.

## Discussion

We assessed the evolutionary patterns of 89 bone-related genes in 47 avian and 39 mammalian genomes and demonstrate that there has been significantly higher positive selective pressure on several of the bone-associated genes of birds, particularly in those involved in bone-regulatory processes. Moreover, just as in birds, flying mammals (bats) had several genes with evolutionary rates that contrasted with the patterns observed in other mammals. These results highlight convergent changes in bone genes in the evolution of flight and the extensive selective pressure that flight triggered in bone-associated genes.

### Body mass and bone-associated genes

The different evolutionary trajectories for developing the capacity to fly in birds and bats led to distinct mechanical and biochemical solutions to the adaptive challenges. Nevertheless, both birds and bats have bones with high mineral content [3] and both have body sizes that approach the predicted theoretical limit, *i.e*, the tradeoff between the mechanical power and the capacity for metabolic output essential for flight [37]. Among different avian orders, skeletal measurements and body mass are correlated, as they are limited by ecological and biomechanical constraints on bone dimensions [38]. The different life habits among birds partially explains the higher correlation between body mass and d_N_/d_S_ that was observed when assessing the dataset including all the bird species. Since this covariance suggests a relaxation on the selective pressure on bone-associated genes in non-flying species, the findings are consistent with the hypothesis that the skeleton of flightless birds can be larger than in flying birds. The absence of this correlation among flying species may reflect their lower variation in the body mass and differences in the foraging habits irrespective of their body size, since bone structure is often associated with the life history of the species [39]. In contrast, extant mammals display a wider range of body mass than extant birds [40], supporting the observed correlation between d_N_/d_S_ and average body mass.

Furthermore, the opposite trend in birds and mammals might partially be explained by the contrasting life-histories of the species in the two clades. Bird evolution seems to have favored size reduction in Neoaves, while in mammals, trends in body mass vary among subclades [36]. This can also explain the higher correlation between d_N_/d_S_ and body mass when bats are included. However, in both scenarios, either including or excluding bats, there was a positive and statistically significant correlation between body mass and d_N_/d_S_.

### Evolutionary rate in flying versus non-flying species

Although vertebrate powered flight is not restricted to birds, flight is more ubiquitous in birds. Powered flight has been linked with low body mass [41], high metabolic rate [42], metabolic efficiency [43], and specialized mechanical systems supported by skeletal adaptations. Yet, many aspects of flight remain unclear, including how bone-related genes evolved in birds and other taxonomic groups such as bats. The high rates of selection that we found for several bone-related genes suggest that the observed variation among avian species is higher than would be expected under models of neutrality. Therefore, the presence of adaptive and positive selection in these genes is likely indicative of a fundamental feature of trait modeling in the evolution of the skeleton. The phylogeny also supports this observation since the incongruence between the species-tree and gene-tree reinforces the hypothesis that flight was a key event that had a noticeable impact on the evolution of bone-associated genes in birds and mammals.

### Extended impact of flight on bone-associated genes

Our results suggest that a relatively small number of genes involved in bone structures may have independently evolved in birds and bats in similar ways that permitted the transition from terrestrial to aerial life styles. Of the 89 bone-associated genes, only 13 showed signatures of selection in both birds (site model) and bats (branch model exhibiting acceleration/deceleration relatively to terrestrial mammals with significant statistical support). The function of these 13 genes, summarized below, probably reflect key genetic pathways and adaptations that enable flight. However, since several of these bone-associated genes are also involved in other processes, the comparison between flying and non-flying species suggests that some of the genes involved in the evolution of flight may also have had other evolutionary constraints (Fig. 1b).

*BMP2* (Bone morphogenetic protein 2) has been implicated in the stimulation of cartilage proliferation and differentiation and in the increase in digit length in bat embryonic forelimbs [44]. Similarly, *PKDCC* (protein kinase domain containing cytoplasmic) is implicated in the control of limbs length, since the target disruption of this gene leads to short limbs [45]. The lengthening of forelimbs was an essential step in the evolution of flight in vertebrates [46, 47]. Birds also share several other features, including a fused cranial bone, which might be linked with *BMP2* [48]. Importantly, several other examples of bone fusion (e.g. vertebrae fusion) have been cited as being crucial for the evolution of flight [49].

*OSR2* (odd-skipped related 2) has been associated with forelimb, hindlimb and craniofacial development [50] and is a likely candidate gene for many of the fundamental changes in the limbs of birds and bats. At the beginning of avian evolution, the allometric coupling of forelimb and hindlimb with body size was disrupted, and as wings began to significantly elongate, they maintained a positive allometric relationship with body size, but their legs significantly shortened [47]. This would have facilitated the diversification of forelimb and hindlimb shapes and sizes that are currently observed in extant birds [47] and which are closely linked with foraging habits in birds and bats [47].

*HOXA11* (homeobox A11) may also be related with bone fusion, as this gene has been reported to influence radio-ulnar fusion [51] and bats may also display partial fusion of those bones (see Additional file 1: Figure S1). Although birds presented no evidence of fusion of the radio and ulna, these bones are typically apneumatic in birds and therefore contain bone marrow; and *HOXA11* has been associated with bone marrow failure syndrome [51]. Interestingly in this gene are detected three homologous sites under positive selection in bats and flying birds, suggestive of functional convergence, likely due to flight evolution (Additional file 8: Table S5 and Additional file 9: Table S6).

*FGF23* (fibroblast growth factor 23), *MEPE* (matrix extracellular phosphoglycoprotein), *NCDN* (neurochondrin), *NOX4* (NADPH oxidase 4) are involved in bone metabolism [52–55]. Bone metabolism genes are often associated with alterations of Bone Mineral Density (BMD) [56], and BMD alterations in birds and bats have previously been linked with flight adaptations [3].

*BMPR1A* (bone morphogenetic protein type IA gene) is involved in bone remodeling, and the ablation of this receptor in osteoblasts increases bone mass [57]. This makes *BMPR1A* a prime candidate for the maintenance of bone strength, which is essential for a stiff, but lightweight skeleton system in flying species [3]. Similarly, *ACVR2B* (activin receptor type-2B) is involved in the control of bone mass, but interestingly is mediated by *GDF-8* (myostatin) which is also involved in improving muscle strength [58].

*PTK2B* is involved in bone resorption [59], a process involved in bone remodeling, during which osteoclasts digest old bone [60]. Bone remodeling is essential to making the necessary adjustments of bone architecture for the mechanical needs of flight [60]. It may well be responsible for alterations that support the increased BMD levels [61] that are observed in both bats and birds.

*CITED2* (Cbp/P300-interacting transactivator, with Glu/Asp-rich carboxyl-terminal) is involved in bone formation [62], but also plays a pivotal role in muscle mass regulation since it also counteracts glucocorticoid-induced muscle atrophy [63]. Flight in vertebrates requires powerful muscles, particularly those connected to sternum bones [64]. *CITED2* has also been linked with some heart diseases [65], which may be of note since birds [66] and small bats [67] possess larger hearts relative to vertebrates of similar size.

*TCF7L2* (Transcription factor 7-like 2) is associated with bone mineralization [68]. However, it is also considered to be the most significant genetic marker that has been linked with Diabetes mellitus Type 2 risk and it is a key regulator of glucose metabolism [69]. The signatures of selection observed in birds and bats in *TCF7L* are remarkable given the high blood glucose levels observed in birds [70] and fruit and nectar-feeding bats [21, 71]. The tolerance of birds and bats to blood-hyperglycemia may therefore be related with the evidence for positive selection observed in our analyses, as flight requires efficient glucose metabolism and efficient transportation to the energy-demanding organs (e.g. flight muscles) that are involved in powered flight [71, 72].

Despite the similarities between bats and birds, extensive positive selection is observed in some genes in birds but is absent in bats, including *P2RX7* and *TPP1,* which are mainly involved in bone resorption [32, 73]. In birds, the pneumatic epithelium that forms the diverticula is capable of extensive resorption of bone material given its close association with osteoclasts [74]. Bone remodeling through resorption may be crucial to the formation of the bone trabeculae and by extension, the formation of the pneumatic bones. Recently, polymorphisms described in *P2RX7* have been associated with osteoporosis in humans [75], which is typically linked with increased bone resorption and a decrease in bone mineral density (BMD) [76]. Here we demonstrated that genes involved in bone remodeling (particularly evident in the sub-process bone resorption) had multiple signals of positive selection in birds, but contrary to osteoporosis, bird bones attain a high value of BMD [3].

### Gene’s functional categories, bone remodelling and their implication in life-habits

Although bone pneumaticity may have facilitated the transition to flight in birds, it may not have been a necessary step, since bats evolved the ability to fly without postcranial skeletal pneumaticity. Pneumatization preceded the origin of avian flight and evolved independently in several groups of bird-line archosaurs (ornithodirans) [77], and therefore cannot be exclusively the result of adaptation for flight [77]. It has been suggested that skeletal pneumaticity, in early evolutionary stages, provided no selective advantage [78] and also did not significantly affect the skeleton through the lightening or remodeling of individual bones [78]. Although skeletal density modulation would have resulted in energetic savings as part of a multi-system response to increased metabolic demands and the acquisition of an extensive postcranial skeleton, pneumaticity may have favored high-performance endothermy [77].

Nevertheless, the finding that genes involved in bone remolding have been subjected to a higher prevalence of positive selection is interesting because: 1) development of postcranial skeletal pneumaticity occurs after hatching [79]; 2) the skeleton is a metabolically active tissue that undergoes continuous remodeling throughout life [60]; and 3) bone remodeling may lead to a more porous bone structure [60]. Bone remodeling involves the removal of mineralized bone by osteoclasts followed by the formation of a bone matrix through the osteoblasts that is subsequently mineralized [60]. It is generally assumed that bone remodeling is essential for maintaining skeletal mechanical properties and mineral homeostasis [80]. Therefore the higher prevalence of positive selection in bone-remodeling genes suggests that bones with higher mineral density were attained as a response to the selective contingencies imposed by flying, including bone remodeling and bone resorption. The similarities among bats and flying birds, bones with high mineral content, suggests that genes involved in bone remodeling probably play a pivotal role in avian diversification and adaptation in a wide range of ecological and behavioral niches.

## Conclusions

The evolution of flight in birds was a pivotal event in their successful adaptation to new ecological niches. However, the transition to flight imposed new challenges on their bone structure. The high rate of positive selection in bone-associated genes in birds suggests that there was a strong link among changes in these genes and the adaptations necessary for flight. Limitations imposed on body size were probably also a key factor in bird evolution, as we have shown here that body mass covaried significantly with the d_N_/d_S_ value only when flightless birds were included. Evidence of adaptive selection in birds and bats also were apparent in genes plausibly linked with bone-remodeling, bone fusion, lengthening of forelimbs, as well as with functions outside the skeleton system, including glucose tolerance that also would have had a major influence on the capacity for powered flight. However, the examples of positive selection that were only observed in birds, such as the evolution of a more-diversified and richer-variety of protein-encoding genes involved in bone resorption (e.g. *TPP1* and *P2RX7)* and the formation of bone trabeculae that are likely critical to the evolution of hollow or pneumatic bones*,* suggest that these might be crucial steps in the evolution of avian flight that are unique to birds.

## Methods

### Sequences and alignment

A list of bone-associated genes was retrieved from the GO database by querying the term “bone” in QuickGO [81]. The resulting list was filtered using unique terms and the correct gene name was mapped using the REST API available in bioDBnet [82]. The gene list was then used in Ensembl Biomart to retrieve the Ensembl Gene ID using *Gallus gallus* as reference. The gene name and/or gene ID was used to search in each genome file that contained the annotated gene sequences from each bird species. The avian dataset derived from 47 bird genomes provided by the Avian Genome Consortium [26] encompasses 89 bone-associated genes (Additional file 2: Table S1), resulting in a total of 3,388 sequences and ~38 species sequences on average per multiple sequence alignment (MSA). Sequences for each gene were translated into amino acids, aligned using MUSCLE [83] and back-translated to nucleotides. Aberrant sequences, containing frame-shifts (e.g. stop codons) and duplicated sequences, were removed from the MSA. The dataset from reptiles was retrieved from the NCBI nucleotide database [84], which encompassed 20 different species (Additional file 17: Figure S6). For *MEPE* only one sequence was retrieved and therefore 88 genes were successfully used (672 sequences, ~7.6 sequences per gene). The sequences were aligned using MUSCLE [83] and back-translated to nucleotides. The mammalian dataset was derived from 39 genomes (2,903 sequences, ~32 per gene) that were manually retrieved from ENSEMBL [28, 29]. The MSA of each gene was built using the same strategy as with the avian genes. The 89 genes were concatenated using SequenceMatrix v 1.7.8 [85] to one MSA containing all the avian data, and a second MSA containing the 89 mammalian genes. A phylogenetic tree was built separately for birds and mammals using the 89 concatenated genes with PhyML v3.0 [86] under the Generalized Time-Reversible (GTR + Г + I) model and the branch-support was provided by aLRT [87]. The obtained phylogenetic trees were compared using TREE-PUZZLE [88].

For the comparison between birds of different flying ability, we included among flightless birds: 1) aquatic birds, *Pygoscelis adeliae* (adelie penguin) and *Aptenodytes forsteri* (emperor penguin), 2) ratites, *Tinamus guttatus* (white-throated tinamou) and *Struthio camelus* (ostrich) and 3) poor or weakly flyers [33], *G. gallus* (chicken) and *Meleagris gallopavo* (turkey), since these can only flap for a short distance but are incapable of sustained flight.

### Site models

CODEML, as implemented in PAML v4.7 [30, 89], was used to test for selection signatures in the avian, mammalian and reptilian bone genes using three models (Models 0, 1 and 2). Model 0 was used to calculate the global d_N_/d_S_ and Model 1 vs Model 2 to identify the sites under positive selection. Sites with significant signatures of selection were retrieved after a post-hoc analysis using Bayesian Empirical Bayes [90]. The tree topology used as the input for the CODEML models for mammals was the tree retrieved from ENSEMBL, for birds was the full-genome derived tree “species tree” [27] (Additional file 18: Figure S5) and for reptiles was adapted from recent publications [91, 92] (Additional file 17: Figure S6). Estimations for d_N_ and d_S_ under Model 0 for each branch showed low levels of saturation (Additional file 19: Table S14).

### Branch models

We tested for selection using branch models with a two-ratio model that allow variation in the d_N_/d_S_ ratio between the background and foreground branches. The two-ratio model was compared against a one-ratio model. In the bird and mammal datasets the “exceptions” (flightless birds and flying mammals) were compared against the flying birds and flightless mammals, providing an understanding of which genes were under differential selection patterns in the two clades. Spearman’s correlations were performed in SPSS v20 [93].

### Branch-site models

The branch-site model detects positive selection when it occurs in sites along particular lineages or labeled branches (foreground branches). This model allows the d_N_/d_S_ ratio either to vary along the sites or the branches on the tree (foreground vs background branches). To compare the effect of flight in bone-associated genes, the terminal branches of flying species in birds were considered to be the foreground branches and the non-flying species the background branches. The sequences were aligned using all sequences and later separated into two different alignments. For each MSA was performed a branch-site model A with ω_2_ = 1 fixed in the null model.

### Correction for multiple testing

All the results from site, branch-site and branches models were corrected for possible multiple testing bias using the procedure of Benjamini and Hochberg [94] as implemented in the program Q-Value [95]. For each *p*-value, we also estimated the corresponding q-value; where the q-value represents the false discovery rate using the critical value 0.05. When the q-value was below the critical *p*-value estimated for the Likelihood-Ratio Test value, the gene was considered to be under positive selection (1), and when above, the gene was considered negatively selected (0).

### Functional classification of bone-associated genes

Functional annotation enrichment analyses were performed using the Database for Annotation, Visualization and Integrated Discovery (DAVID v6.7) [96, 97]. Each derived gene list was processed in DAVID for functional terms using *Homo sapiens* as background. Venn diagrams were generated using VENNY [98].

### Correlation model between body mass and bone-associated genes

CoEvol 1.3c [34] implements a phylogenetic model that correlates the evolution of substitution rates (e.g. d_s_, ω (d_N_/d_S_)) with continuous phenotypic characters (e.g. body mass, longevity). The MSA of the 89 bone-associated genes was divided into two different datasets, one including all birds and the other restricted to only the flying bird species. CoEvol was ran under two different phylogenetic assumptions: 1) using the species-tree used in the evolutionary analysis; 2) using the gene-based tree estimated for birds and mammals with the 89 concatenated genes in PhyML v3.0 and the Generalized Time-Reversible (GTR + Г + I) evolutionary model. To ensure convergence, we ran two different chains to at least an effective number of 50. Calibration of the tree was done using the divergence-time-based option in TimeTree [99] (Additional file 20: Table S15) and body mass estimates are provided in a supplemental table (Additional file 21: Table S16).

CoEvol models evolutionary rates of substitution and phenotypic characters as a multivariate Brownian diffusion process along the branches, correcting for the uncertainty about branch lengths and substitution history in the phylogenetic tree. Correlations among rates of substitution and phenotypic characters were calculated with posterior probabilities varying from 0 to 1 using a Bayesian Markov chain Monte Carlo and correcting for phylogenetic inertia using the independent contrast method. Posterior probabilities close to 0 indicate a negative correlation while values close to 1 indicate a positive correlation. Cut-offs of pp < 0.05 and pp > 0.95 suggest negative or positive covariance, respectively, between the substitution rates and the phenotypic trait. The CoEvol analyses were run for at least 2000 points for both phylogenetic trees (species tree and gene tree), for all genes and only positively selected genes in each clade (mammals and birds).

## Additional files

Additional file 1: Figure S1. Skeletal adaptations to flight in bats. Skeleton of Large flying fox (adapted from Wikimedia Commons licensed under a Creative Commons Attribution-Share Alike 3.0 Unported (CC BY-SA 3.0)) and the key features observed in bats skeleton system. The typical bone structure of long bones is highlighted in the light blue box (adapted from Wikimedia Commons licensed under a Creative Commons Attribution-Share Alike 3.0 Unported (CC BY-SA 3.0)). (DOC 331 kb) Additional file 2: Table S1. List of bone associated genes used in this study. (DOC 61 kb) Additional file 3: Figure S2. Genomic location of bone-associated genes. The circular ideogram represents the genomic location of bone-associated genes in four of the studied species. Each end-line represents the location of the bone-associated genes. Blue indicates human chromosomes (mammal representative). Dark orange the zebra finch (flying species), green the chicken and yellow the turkey (flightless species). (DOC 896 kb) Additional file 4: Table S2. Positively selected sites of bone-associated genes in Mammalian dataset after multiple testing correction. The alignment length is on Amino acids (aa). Gene in bold are positively selected under the comparison M2a vs M1a. Q-value estimations for multiple testing are represented as positive selected (1) and negative selected (0). (DOC 88 kb) Additional file 5: Table S3. Positively selected sites of bone-associated genes in Avian dataset after multiple testing correction. The alignment length is on Amino acids (aa). Gene in bold are positively selected under the comparison M2a vs M1a. Q-value estimations for multiple testing are represented as positive selected (1) and negative selected (0). (DOC 88 kb) Additional file 6: Table S4. Positively selected sites of bone-associated genes in Reptilian dataset after multiple testing correction. The alignment length is on Amino acids (aa). Bold represents statistical significance (*p* < 0.05). Q-value estimations for multiple testing are represented as positive selected (1) and negative selected (0). (DOC 158 kb) Additional file 7: Figure S3. Venn diagrams of positively-selected bone-associated genes. The intersection between the positively selected genes in the three clades, birds, mammals and reptiles. (DOC 149 kb) Additional file 8: Table S5. Branch-site model for birds. Genes were the alternate model was preferred relatively to the null model are highlighted as italic and underlined positively selected. (DOC 141 kb) Additional file 9: Table S6. Branch-site model for mammals. Genes without flying mammals present in the alignment are marked (###). (DOC 138 kb) Additional file 10: Table S7. Results from the nested models (M0, M1a, M2a) likelihood ratio test results PAML from Avian dataset excluding flightless birds. The alignment length is on Amino acids (aa). Bold represents statistical significance (*p* < 0.05). Q-value estimations for multiple testing are represented as positive selected (1) and negative selected (0). (DOC 162 kb) Additional file 11: Table S8. Results from the nested models (M0, M1a, M2a) likelihood ratio test results PAML from Mammalian dataset excluding bats. The alignment length is on Amino acids (aa). Bold represents statistical significance (*p* < 0.05). Q-value estimations for multiple testing are represented as positive selected (1) and negative selected (0). (DOC 162 kb) Additional file 12: Table S9. Branch model for birds. Bold represents statistical significance (*p* < 0.05). Q-value estimations for multiple testing are represented as positive selected (1) and negative selected (0). (DOC 114 kb) Additional file 13: Table S10. Branch model for mammals. Genes without flying mammals present in the alignment are marked (###). Bold represents statistical significance (*p* < 0.05). Q-value estimations for multiple testing are represented as positive selected (1) and negative selected (0). (DOC 114 kb) Additional file 14: Figure S4. Body mass association with ω (d_N_/d_S_). Avian cladogram showing from CoEvol, the labels are the estimated ω (minimum maximum) for each branch on top and the estimated weight (minimum maximum). (DOC 423 kb) Additional file 15: Table S11. Covariance between d_S_, ω (d_N_/d_S_), gc content, and the three body mass measures (minimum, maximum and average) in 45 bird genomes using gene-based tree. The upper triangle shows the values obtained for all birds and the lower triangle excluding flightless birds. Each cell represent the covariance values and posterior probability are the bracketed values, posterior probability (** - < = 0.025 or > =0.975; * - < =0.05 or > =0.95) are highlighted in bold for the statistically significant correlations. (DOC 35 kb) Additional file 16: Table S12. Covariance between d_S_, ω (d_N_/d_S_), gc content, and the three body mass measures (minimum, maximum and average) in 39 mammalian genomes using gene-based tree. The upper triangle shows the values obtained for all mammals and the lower triangle excluding bats. Each cell represent the covariance values and posterior probability are the bracketed values, posterior probability (** - < = 0.025 or > =0.975; * - < =0.05 or > =0.95) are highlighted in bold for the statistically significant correlations. (DOC 35 kb) Additional file 17: Figure S6. Phylogenetic trees of reptiles used in CODEML analysis. (DOC 90 kb) Additional file 18: Figure S5. Avian and Mammalian phylogenetic trees used in CODEML analysis. Lineages of flightless birds are highlighted in red, while flying mammals are highlighted in blue. (DOC 339 kb) Additional file 19: Table S13. Estimation of d_N_ and d_S_ for each branch under Model 0. For each branch, average of d_N_ and d_S_ and the corresponding standard deviation. (DOC 165 kb) Additional file 20: Table S14. Divergence limit estimations. Calibration points retrieved from TimeTree. (DOC 35 kb) Additional file 21: Table S15. Body mass in birds and mammals. (DOCX 46 kb)

## Abbreviations

1sKH: one sided KH test based on pairwise SH tests
BMD: bone mineral density
ELW: expected likelihood weight
MSA: multiple sequence alignment
pp: posterior probability
SH: Shimodaira-Hasegawa
ω: omega

## References

[1] Puttick MN, Thomas GH, Benton MJ. High Rates of Evolution Preceded the Origin of Birds. Evolution. 2014;68(5):1497–1510. 10.1111/evo.12363 PMC4289940 24471891

[2] Smith FA, Brown JH, Haskell JP, Lyons SK, Alroy J, Charnov EL, Dayan T, Enquist BJ, Ernest SK, Hadly EA (2004). Similarity of mammalian body size across the taxonomic hierarchy and across space and time. Am Nat.

[3] Dumont ER (2010). Bone density and the lightweight skeletons of birds. Proc Biol Sci..

[4] Hone DW, Dyke GJ, Haden M, Benton MJ (2008). Body size evolution in Mesozoic birds. J Evol. Biol..

[5] Alexander RM (1998). All-time giants: the largest animals and their problems. Palaeontology.

[6] Gutzwiller SC, Su A, O’Connor PM (2013). Postcranial pneumaticity and bone structure in two clades of neognath birds. Anat Rec (Hoboken).

[7] Fastovsky DE, Weishampel DB. The Evolution and Extinction of the Dinosaurs. Cambridge, UK: Cambridge University Press; 2005.

[8] Currey JD (2003). The many adaptations of bone. J Biomech.

[9] Smith TD, Rossie JB, Cooper GM, Mooney MP, Siegel MI (2005). Secondary pneumatization of the maxillary sinus in callitrichid primates: insights from immunohistochemistry and bone cell distribution. Anat Rec A Discov Mol Cell Evol Biol..

[10] Prange HD, Anderson JF, Rahn H. Scaling of skeletal mass to body mass in birds and mammals. American Naturalist. 1979;1:103–122.

[11] Fedducia A (1996). The origin and evolution of birds.

[12] Podulka S, Rohrbaugh RW, Bonney R (2004). Handbook of bird biology.

[13] Freeman S. Biological science. Upper Saddle River, N.J.: Pearson Prentice Hall; 2005.

[14] Gill FB. Ornithology. 3rd. In: New York: WH Freeman. xxvi;2007.

[15] Cubo J, Casinos A. Scaling of skeletal element mass in birds. Belgian J Zool. 1994;124:127–137.

[16] Dececchi TA, Larsson HC (2011). Assessing arboreal adaptations of bird antecedents: testing the ecological setting of the origin of the avian flight stroke. PLoS One.

[17] Maurer BA, Brown JH, Dayan T, Enquist BJ, Ernest SM, Hadly EA, Haskell JP, Jablonski D, Jones KE, Kaufman DM (2004). Similarities in body size distributions of small-bodied flying vertebrates. Evol. Ecol. Res..

[18] Thomas SP, Follette DB, Thomas GS (1995). Metabolic and ventilatory adjustments and tolerance of the bat Pteropus poliocephalus to acute hypoxic stress. Comp Biochem Physiol A Physiol.

[19] Ward S, Bishop CM, Woakes AJ, Butler PJ (2002). Heart rate and the rate of oxygen consumption of flying and walking barnacle geese (Branta leucopsis) and bar-headed geese (Anser indicus). J Exp. Biol..

[20] Braun EJ, Sweazea KL (2008). Glucose regulation in birds. Comp Biochem Physiol B Biochem Mol Biol.

[21] Kelm DH, Simon R, Kuhlow D, Voigt CC, Ristow M (2011). High activity enables life on a high-sugar diet: blood glucose regulation in nectar-feeding bats. Proc. Biol. Sci./R Soc.

[22] Brunet-Rossinni AK (2004). Reduced free-radical production and extreme longevity in the little brown bat (< i > Myotis lucifugus</i>) versus two non-flying mammals. Mech Ageing Dev.

[23] Barja G (1998). Mitochondrial Free Radical Production and Aging in Mammals and Birdsa. Ann N Y Acad Sci.

[24] Caviedes-Vidal E, McWhorter TJ, Lavin SR, Chediack JG, Tracy CR, Karasov WH (2007). The digestive adaptation of flying vertebrates: high intestinal paracellular absorption compensates for smaller guts. Proc Natl Acad Sci U S A.

[25] Bassett JH, Gogakos A, White JK, Evans H, Jacques RM, van der Spek AH, Ramirez-Solis R, Ryder E, Sunter D, Boyde A (2012). Rapid-throughput skeletal phenotyping of 100 knockout mice identifies 9 new genes that determine bone strength. PLoS Genet.

[26] Zhang G, Li C, Li Q, Li B, Larkin DM, Lee C, Storz JF, Antunes A, Greenwold MJ, Meredith RW (2014). Comparative genomics reveals insights into avian genome evolution and adaptation. Science.

[27] Jarvis ED, Mirarab S, Aberer AJ, Li B, Houde P, Li C, Ho SY, Faircloth BC, Nabholz B, Howard JT (2014). Whole-genome analyses resolve early branches in the tree of life of modern birds. Science.

[28] Flicek P, Ahmed I, Amode MR, Barrell D, Beal K, Brent S, Carvalho-Silva D, Clapham P, Coates G, Fairley S (2013). Ensembl 2013. Nucleic Acids Res.

[29] Flicek P, Amode MR, Barrell D, Beal K, Billis K, Brent S, Carvalho-Silva D, Clapham P, Coates G, Fitzgerald S (2014). Ensembl 2014. Nucleic Acids Res.

[30] Yang Z (2007). PAML 4: phylogenetic analysis by maximum likelihood. Mol Biol Evol.

[31] Jorgensen NR, Husted LB, Skarratt KK, Stokes L, Tofteng CL, Kvist T, Jensen JE, Eiken P, Brixen K, Fuller S (2012). Single-nucleotide polymorphisms in the P2X7 receptor gene are associated with post-menopausal bone loss and vertebral fractures. Eur J Hum Genet.

[32] Page AE, Fuller K, Chambers TJ, Warburton MJ (1993). Purification and characterization of a tripeptidyl peptidase I from human osteoclastomas: evidence for its role in bone resorption. Arch Biochem Biophys.

[33] Shen YY, Shi P, Sun YB, Zhang YP (2009). Relaxation of selective constraints on avian mitochondrial DNA following the degeneration of flight ability. Genome Res.

[34] Lartillot N, Poujol R (2011). A phylogenetic model for investigating correlated evolution of substitution rates and continuous phenotypic characters. Mol Biol Evol.

[35] Weber CC, Boussau B, Romiguier J, Jarvis ED, Ellegren H (2014). Evidence for GC-biased gene conversion as a driver of between-lineage differences in avian base composition. Genome Biol.

[36] Nabholz B, Uwimana N, Lartillot N. Reconstructing the phylogenetic history of long-term effective population size and life-history traits using patterns of amino-acid replacement in mitochondrial genomes of mammals and birds. Genome Biol Evol. 2013;evt083:1273–1290. 10.1093/gbe/evt083 PMC3730341 23711670

[37] Maina J (2000). What it takes to fly: the structural and functional respiratory refinements in birds and bats. J. Exp. Biol..

[38] Field DJ, Lynner C, Brown C, Darroch SA (2013). Skeletal Correlates for Body Mass Estimation in Modern and Fossil Flying Birds. PLoS One.

[39] Smith ND (2012). Body mass and foraging ecology predict evolutionary patterns of skeletal pneumaticity in the diverse “waterbird” clade. Evolution.

[40] Bouzat JL (2000). The importance of control populations for the identification and management of genetic diversity. Genetica.

[41] Schmidt-Wellenburg CA, Engel S, Visser GH (2008). Energy expenditure during flight in relation to body mass: effects of natural increases in mass and artificial load in Rose Coloured Starlings. J Comp Physiol B.

[42] Munshi-South J, Wilkinson GS (2010). Bats and birds: Exceptional longevity despite high metabolic rates. Ageing Res Rev.

[43] Morris CR, Nelson FE, Askew GN (2010). The metabolic power requirements of flight and estimations of flight muscle efficiency in the cockatiel (Nymphicus hollandicus). J Exp Biol.

[44] Sears KE, Behringer RR, Rasweiler JJ, Niswander LA (2006). Development of bat flight: morphologic and molecular evolution of bat wing digits. Proc Natl Acad Sci U S A.

[45] Imuta Y, Nishioka N, Kiyonari H, Sasaki H (2009). Short limbs, cleft palate, and delayed formation of flat proliferative chondrocytes in mice with targeted disruption of a putative protein kinase gene, Pkdcc (AW548124). Dev Dyn.

[46] Padian K, Chiappe LM (1998). The origin of birds and their flight. Sci Am.

[47] Dececchi TA, Larsson HC (2013). Body and limb size dissociation at the origin of birds: uncoupling allometric constraints across a macroevolutionary transition. Evolution.

[48] Chen D, Zhao M, Mundy GR (2004). Bone morphogenetic proteins. Growth Factors.

[49] Cubo J, Casinos A (2000). Incidence and mechanical significance of pneumatization in the long bones of birds. Zool J Linn Soc.

[50] Lan Y, Kingsley PD, Cho ES, Jiang R (2001). Osr2, a new mouse gene related to Drosophila odd-skipped, exhibits dynamic expression patterns during craniofacial, limb, and kidney development. Mech Dev.

[51] Thompson AA, Nguyen LT (2000). Amegakaryocytic thrombocytopenia and radio-ulnar synostosis are associated with HOXA11 mutation. Nat Genet.

[52] Rowe PS. Regulation of Bone − Renal Mineral and Energy Metabolism: The PHEX, FGF23, DMP1, MEPE ASARM Pathway. Critical Reviews™ in Eukaryotic Gene Expression. 2012;22(1):61–86. 10.1615/critreveukargeneexpr.v22.i1.50 PMC3362997 22339660

[53] Dateki M, Horii T, Kasuya Y, Mochizuki R, Nagao Y, Ishida J, Sugiyama F, Tanimoto K, Yagami K, Imai H (2005). Neurochondrin negatively regulates CaMKII phosphorylation, and nervous system-specific gene disruption results in epileptic seizure. J Biol Chem.

[54] Fukumoto S (2008). Physiological regulation and disorders of phosphate metabolism--pivotal role of fibroblast growth factor 23. Intern Med.

[55] Goettsch C, Babelova A, Trummer O, Erben RG, Rauner M, Rammelt S, Weissmann N, Weinberger V, Benkhoff S, Kampschulte M (2013). NADPH oxidase 4 limits bone mass by promoting osteoclastogenesis. J Clin Invest.

[56] Alexopoulou O, Jamart J, Devogelaer JP, Brichard S, de Nayer P, Buysschaert M (2006). Bone density and markers of bone remodeling in type 1 male diabetic patients. Diabetes Metab.

[57] Baud'huin M, Solban N, Cornwall-Brady M, Sako D, Kawamoto Y, Liharska K, Lath D, Bouxsein ML, Underwood KW, Ucran J, et al. A soluble bone morphogenetic protein type IA receptor increases bone mass and bone strength. Proc Natl Acad Sci U S A. 2012;109(30):12207–12. 10.1073/pnas.1204929109 PMC3409793 22761317

[58] Hamrick MW (2010). Myostatin (GDF-8) as a therapeutic target for the prevention of osteoporotic fractures. IBMS BoneKEy.

[59] Miyazaki T, Sanjay A, Neff L, Tanaka S, Horne W, Baron R (2004). Src kinase activity is essential for osteoclast function. J Biol Chem.

[60] Hadjidakis DJ, Androulakis II (2006). Bone remodeling. Ann N Y Acad Sci.

[61] Arrabal‐Polo MA, Arrabal‐Martin M, de Haro‐Munoz T, Lopez‐Leon VM, Merino‐Salas S, Ochoa‐Hortal MA, et al. Mineral density and bone remodelling markers in patients with calcium lithiasis. BJU Int. 2011;108(11):1903–8. 10.1111/j.1464-410X.2011.10167.x 21554525

[62] Lee JY, Taub PJ, Wang L, Clark A, Zhu LL, Maharam ER, et al. Identification of CITED2 as a negative regulator of fracture healing. Biochem Biophys Res Commun. 2009;387(4):641–5. 10.1016/j.bbrc.2009.07.029 PMC3008352 19607804

[63] Tobimatsu K, Noguchi T, Hosooka T, Sakai M, Inagaki K, Matsuki Y, et al. Overexpression of the transcriptional coregulator Cited2 protects against glucocorticoid-induced atrophy of C2C12 myotubes. Biochem Biophys Res Commun. 2009;378(3):399–403. 10.1016/j.bbrc.2008.11.062 19032942

[64] Olson SL, Feduccia A (1979). Flight capability and the pectoral girdle of Archaeopteryx.

[65] Li Q, Pan H, Guan L, Su D, Ma X (2012). CITED2 mutation links congenital heart defects to dysregulation of the cardiac gene VEGF and PITX2C expression. Biochem Biophys Res Commun.

[66] Grubb BR (1983). Allometric relations of cardiovascular function in birds. Am. J. Physiol. Heart Circ. Physiol..

[67] Canals M, Atala C, Grossi B, Iriarte-Díaz J (2005). Relative size of hearts and lungs of small bats. Acta Chiropterologica.

[68] Friedman MS, Oyserman SM, Hankenson KD (2009). Wnt11 promotes osteoblast maturation and mineralization through R-spondin 2. J. Biol. Chem..

[69] Vaquero AR, Ferreira NE, Omae SV, Rodrigues MV, Teixeira SK, Krieger JE, Pereira AC (2012). Using gene-network landscape to dissect genotype effects of TCF7L2 genetic variant on diabetes and cardiovascular risk. Physiol Genomics.

[70] Szwergold BS, Miller CB. Potential of birds to serve as a pathology-free model of Type 2 Diabetes, 1: Is the apparent absence of the RAGE gene a factor in the resistance of avian organisms to chronic hyperglycemia? Rejuvenation Research. 2013(ja);17(1):54–61. 10.1089/rej.2013.1498 24313337

[71] Shen B, Han X, Zhang J, Rossiter SJ, Zhang S (2012). Adaptive evolution in the glucose transporter 4 gene Slc2a4 in Old World fruit bats (family: Pteropodidae). PLoS One.

[72] Shen YY, Liang L, Zhu ZH, Zhou WP, Irwin DM, Zhang YP (2010). Adaptive evolution of energy metabolism genes and the origin of flight in bats. Proc Natl Acad Sci U S A.

[73] Armstrong S, Pereverzev A, Dixon SJ, Sims SM (2009). Activation of P2X7 receptors causes isoform-specific translocation of protein kinase C in osteoclasts. J Cell Sci.

[74] Witmer LM (1997). The evolution of the antorbital cavity of archosaurs: a study in soft-tissue reconstruction in the fossil record with an analysis of the function of pneumaticity. J. Vertebr. Paleontol..

[75] Wesselius A, Bours MJ, Henriksen Z, Syberg S, Petersen S, Schwarz P, Jorgensen NR, van Helden S, Dagnelie PC (2013). Association of P2X7 receptor polymorphisms with bone mineral density and osteoporosis risk in a cohort of Dutch fracture patients. Osteoporos Int.

[76] Riggs BL (2000). The mechanisms of estrogen regulation of bone resorption. J. Clin. Invest..

[77] Benson RB, Butler RJ, Carrano MT, O’Connor PM (2012). Air-filled postcranial bones in theropod dinosaurs: physiological implications and the ‘reptile’-bird transition. Biol Rev Camb Philos Soc.

[78] Wedel MJ, Taylor MP (2013). Caudal pneumaticity and pneumatic hiatuses in the sauropod dinosaurs giraffatitan and apatosaurus. PLoS One.

[79] Hogg DA (1984). The distribution of pneumatisation in the skeleton of the adult domestic fowl. J Anat.

[80] Parfitt AM (2002). Targeted and nontargeted bone remodeling: relationship to basic multicellular unit origination and progression. Bone.

[81] Binns D, Dimmer E, Huntley R, Barrell D, O’Donovan C, Apweiler R (2009). QuickGO: a web-based tool for Gene Ontology searching. Bioinformatics.

[82] Mudunuri U, Che A, Yi M, Stephens RM (2009). bioDBnet: the biological database network. Bioinformatics.

[83] Edgar RC (2004). MUSCLE: multiple sequence alignment with high accuracy and high throughput. Nucleic Acids Res.

[84] Pruitt KD, Tatusova T, Maglott DR (2005). NCBI Reference Sequence (RefSeq): a curated non-redundant sequence database of genomes, transcripts and proteins. Nucleic Acids Res.

[85] Vaidya G, Lohman DJ, Meier R (2011). SequenceMatrix: concatenation software for the fast assembly of multi-gene datasets with character set and codon information. Cladistics.

[86] Guindon S, Delsuc F, Dufayard JF, Gascuel O (2009). Estimating maximum likelihood phylogenies with PhyML. Methods Mol Biol.

[87] Anisimova M, Gascuel O (2006). Approximate likelihood-ratio test for branches: A fast, accurate, and powerful alternative. Syst Biol.

[88] Schmidt HA, Strimmer K, Vingron M, von Haeseler A (2002). TREE-PUZZLE: maximum likelihood phylogenetic analysis using quartets and parallel computing. Bioinformatics.

[89] Yang Z (1997). PAML: a program package for phylogenetic analysis by maximum likelihood. Comput Appl Biosci.

[90] Yang Z, Wong WS, Nielsen R (2005). Bayes empirical bayes inference of amino acid sites under positive selection. Mol Biol Evol.

[91] Pincheira-Donoso D, Bauer AM, Meiri S, Uetz P (2013). Global taxonomic diversity of living reptiles. PLoS One.

[92] Brown RM, Siler CD, Das I, Min Y (2012). Testing the phylogenetic affinities of Southeast Asia’s rarest geckos: Flap-legged geckos (Luperosaurus), Flying geckos (Ptychozoon) and their relationship to the pan-Asian genus Gekko. Mol Phylogenet Evol.

[93] SPSS S, v20 (2011). IBM SPSS Statistics for Windows. Version 20.0 edn.

[94] Benjamini Y, Hochberg Y. Controlling the false discovery rate: a practical and powerful approach to multiple testing. Journal of the Royal Statistical Society Series B (Methodological). 1995;57(1):289–300.

[95] Storey JD, Tibshirani R (2003). Statistical significance for genomewide studies. Proc Natl Acad Sci.

[96] da Huang W, Sherman BT, Lempicki RA (2009). Systematic and integrative analysis of large gene lists using DAVID bioinformatics resources. Nat Protoc.

[97] Huang d W, Sherman BT, Tan Q, Kir J, Liu D, Bryant D, Guo Y, Stephens R, Baseler MW, Lane HC (2007). DAVID Bioinformatics Resources: expanded annotation database and novel algorithms to better extract biology from large gene lists. Nucleic Acids Res.

[98] Oliveros JC. VENNY. In: An interactive tool for comparing lists with Venn Diagrams. 2007. http://bioinfogp.cnb.csic.es/tools/venny/index.html. Accessed July 2015.

[99] Hedges SB, Dudley J, Kumar S (2006). TimeTree: a public knowledge-base of divergence times among organisms. Bioinformatics.

