# Supplementary material for: Bone-associated gene evolution and the origin of flight in birds
Source: BMC Genomics. 2016 May 18;17:371. doi: 10.1186/s12864-016-2681-7 (PMC4870793; doi:10.1186/s12864-016-2681-7)
Supplement: Additional file 1: Figure S1. — Skeletal adaptations to flight in bats. Skeleton of Large flying fox (adapted from Wikimedia Commons licensed under a Creative Commons Attribution-Share Alike 3.0 Unported (CC BY-SA 3.0)) and the key features observed in bats skeleton system. The typical bone structure of long bones is highlighted in the light blue box (adapted from Wikimedia Commons licensed under a Creative Commons Attribution-Share Alike 3.0 Unported (CC BY-SA 3.0)). (DOC 331 kb) [file 12864_2016_2681_MOESM1_ESM.doc]

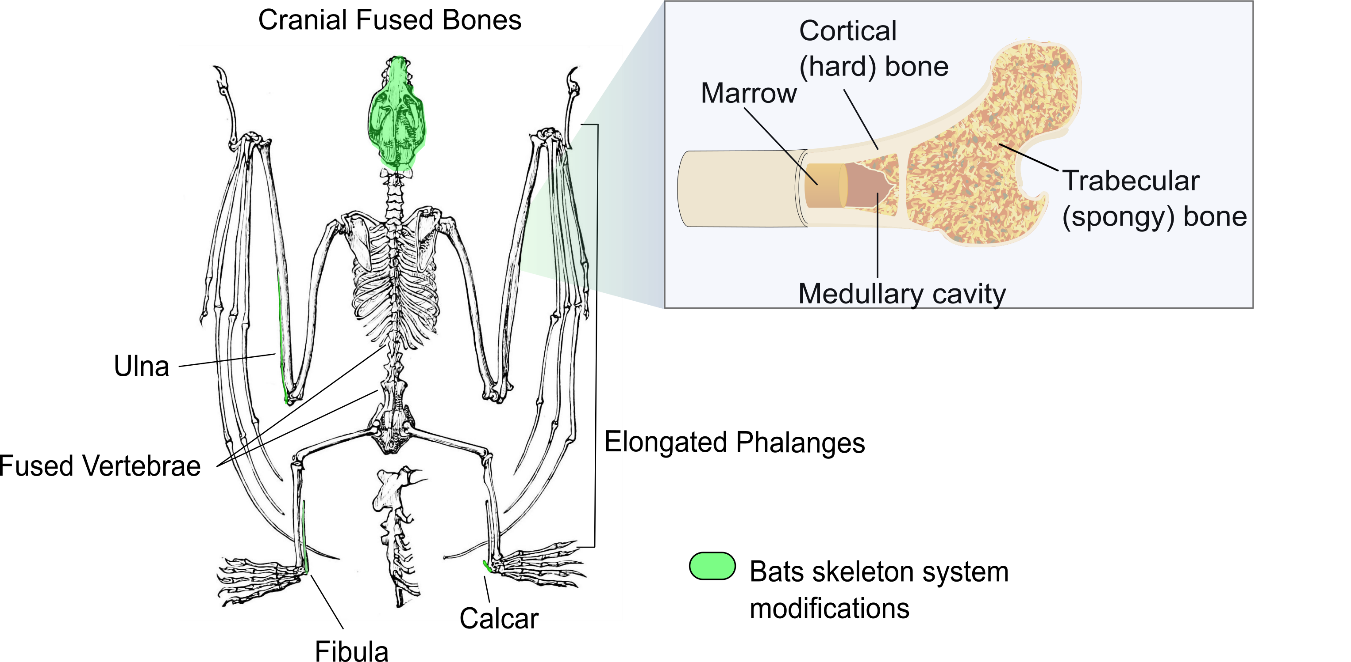


# **Additional file 1: Figure S1 -** Skeletal adaptations to flight in bats**. Skeleton of large flying fox (adapted from Wikimedia Commons licensed under a Creative Commons Attribution-Share Alike 3.0 Unported (CC BY-SA 3.0)) and the key features observed in bats skeleton system. The typical bone structure of long bones is highlighted in the light blue box (adapted from Wikimedia Commons licensed under a Creative Commons Attribution-Share Alike 3.0 Unported (CC BY-SA 3.0)).**
