# Supplementary material for: Bone-associated gene evolution and the origin of flight in birds
Source: BMC Genomics. 2016 May 18;17:371. doi: 10.1186/s12864-016-2681-7 (PMC4870793; doi:10.1186/s12864-016-2681-7)
Supplement: Additional file 2: Table S1. — List of bone associated genes used in this study. (DOC 61 kb) [file 12864_2016_2681_MOESM2_ESM.doc]

# Additional file 2: Table S1 - List of bone associated genes used in this study.

| Gene Symbol | Approved Name |
| --- | --- |
| *ACVR2A* | activin A receptor, type IIA |
| *ACVR2B* | activin A receptor, type IIB |
| *ADAM8* | ADAM metallopeptidase domain 8 |
| *AHSG* | alpha-2-HS-glycoprotein |
| *ANKH* | ANKH inorganic pyrophosphate transport regulator |
| *AQP1* | aquaporin 1 (Colton blood group) |
| *ASPN* | asporin |
| *BCOR* | BCL6 corepressor |
| *BMP2* | bone morphogenetic protein 2 |
| *BMP7* | bone morphogenetic protein 7 |
| *BMPR1A* | bone morphogenetic protein receptor, type IA |
| *CA2* | carbonic anhydrase II |
| *CARM1* | coactivator-associated arginine methyltransferase 1 |
| *CBS* | cystathionine-beta-synthase |
| *CD38* | CD38 molecule |
| *CDX1* | caudal type homeobox 1 |
| *CER1* | cerberus 1, DAN family BMP antagonist |
| *CITED2* | Cbp/p300-interacting transactivator, with Glu/Asp-rich carboxy-terminal domain, 2 |
| *COL2A1* | collagen, type II, alpha 1 |
| *CREB3L1* | cAMP responsive element binding protein 3-like 1 |
| *CTHRC1* | collagen triple helix repeat containing 1 |
| *CTSK* | cathepsin K |
| *DLX5* | distal-less homeobox 5 |
| *DUOX2* | dual oxidase 2 |
| *DYM* | dymeclin |
| *EIF2AK3* | eukaryotic translation initiation factor 2-alpha kinase 3 |
| *FBXL15* | F-box and leucine-rich repeat protein 15 |
| *FGF23* | fibroblast growth factor 23 |
| *FGF8* | fibroblast growth factor 8 (androgen-induced) |
| *GAS6* | growth arrest-specific 6 |
| *GHR* | growth hormone receptor |
| *GPLD1* | glycosylphosphatidylinositol specific phospholipase D1 |
| *GPM6B* | glycoprotein M6B |
| *GREM1* | gremlin 1, DAN family BMP antagonist |
| *HOXA11* | homeobox A11 |
| *HOXB4* | homeobox B4 |
| *HOXD11* | homeobox D11 |
| *HSD17B2* | hydroxysteroid (17-beta) dehydrogenase 2 |
| *IAPP* | islet amyloid polypeptide |
| *IFITM5* | interferon induced transmembrane protein 5 |
| *IGF1* | insulin-like growth factor 1 (somatomedin C) |
| *IHH* | indian hedgehog |
| *IL6* | interleukin 6 (interferon, beta 2) |
| *IL7* | interleukin 7 |
| *INPP5D* | inositol polyphosphate-5-phosphatase, 145kDa |
| *KLF10* | Kruppel-like factor 10 |
| *LRP6* | low density lipoprotein receptor-related protein 6 |
| *LRRC17* | leucine rich repeat containing 17 |
| *MC4R* | melanocortin 4 receptor |
| *MEF2A* | myocyte enhancer factor 2A |
| *MEF2C* | myocyte enhancer factor 2C |
| *MEPE* | matrix extracellular phosphoglycoprotein |
| *MGP* | matrix Gla protein |
| *MITF* | microphthalmia-associated transcription factor |
| *MMP2* | matrix metallopeptidase 2 (gelatinase A, 72kDa gelatinase, 72kDa type IV collagenase) |
| *MSX1* | msh homeobox 1 |
| *NBR1* | neighbor of BRCA1 gene 1 |
| *NCDN* | neurochondrin |
| *NF1* | neurofibromin 1 |
| *NOX4* | NADPH oxidase 4 |
| *OSR2* | odd-skipped related transciption factor 2 |
| *P2RX7* | purinergic receptor P2X, ligand-gated ion channel, 7 |
| *PAPSS2* | 3'-phosphoadenosine 5'-phosphosulfate synthase 2 |
| *PKDCC* | protein kinase domain containing, cytoplasmic |
| *PLA2G4A* | phospholipase A2, group IVA (cytosolic, calcium-dependent) |
| *PLXNB1* | plexin B1 |
| *PTGER4* | prostaglandin E receptor 4 (subtype EP4) |
| *PTH* | parathyroid hormone |
| *PTK2B* | protein tyrosine kinase 2 beta |
| *PTN* | pleiotrophin |
| *SBDS* | Shwachman-Bodian-Diamond syndrome |
| *SFRP1* | secreted frizzled-related protein 1 |
| *SFRP2* | secreted frizzled-related protein 2 |
| *SH3PXD2B* | SH3 and PX domains 2B |
| *SPP2* | secreted phosphoprotein 2, 24kDa |
| *SRD5A1* | steroid-5-alpha-reductase, alpha polypeptide 1 (3-oxo-5 alpha-steroid delta 4-dehydrogenase alpha 1) |
| *SRGN* | serglycin |
| *SULF1* | sulfatase 1 |
| *SULF2* | sulfatase 2 |
| *SYK* | spleen tyrosine kinase |
| *TCF7L2* | transcription factor 7-like 2 (T-cell specific, HMG-box) |
| *TFRC* | transferrin receptor |
| *TGFB3* | transforming growth factor, beta 3 |
| *TNFAIP3* | tumor necrosis factor, alpha-induced protein 3 |
| *TPH1* | tryptophan hydroxylase 1 |
| *TPP1* | tripeptidyl peptidase I |
| *TRAF6* | TNF receptor-associated factor 6, E3 ubiquitin protein ligase |
| *TUFT1* | tuftelin 1 |
| *VEGFA* | vascular endothelial growth factor A |
