# Supplementary material for: Bone-associated gene evolution and the origin of flight in birds
Source: BMC Genomics. 2016 May 18;17:371. doi: 10.1186/s12864-016-2681-7 (PMC4870793; doi:10.1186/s12864-016-2681-7)
Supplement: Additional file 3: Figure S2. — Genomic location of bone-associated genes. The circular ideogram represents the genomic location of bone-associated genes in four of the studied species. Each end-line represents the location of the bone-associated genes. Blue indicates human chromosomes (mammal representative). Dark orange the zebra finch (flying species), green the chicken and yellow the turkey (flightless species). (DOC 896 kb) [file 12864_2016_2681_MOESM3_ESM.doc]

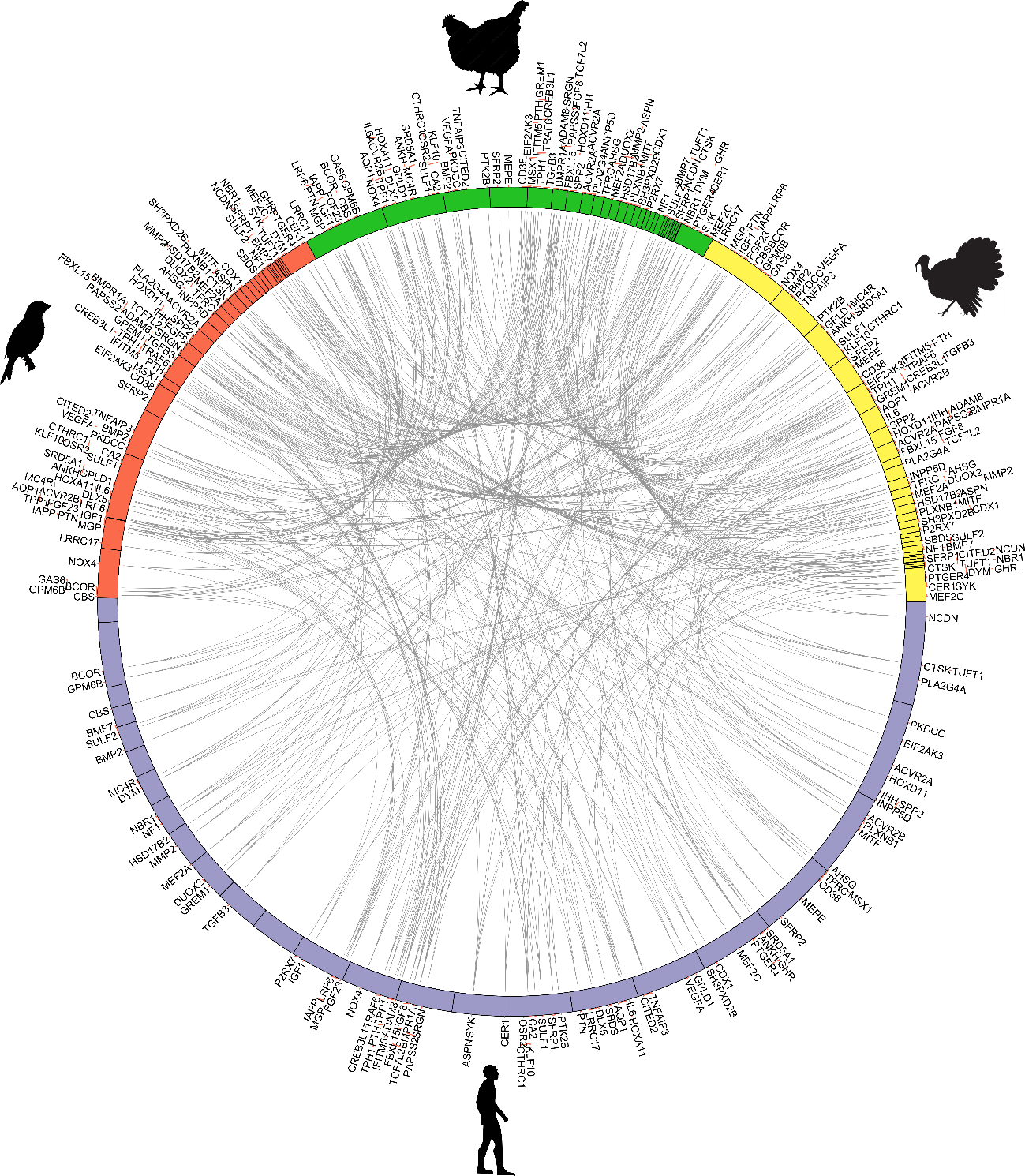


# Additional file 3: Figure S2 - Genomic location of bone-associated genes. The circular ideogram represents the genomic location of bone-associated genes in four of the studied species. Each end-line represents the location of the bone-associated genes. Blue indicates human chromosomes (mammal representative). Dark orange the zebra finch (flying species), green the chicken and yellow the turkey (flightless species).
