# Supplementary material for: Bone-associated gene evolution and the origin of flight in birds
Source: BMC Genomics. 2016 May 18;17:371. doi: 10.1186/s12864-016-2681-7 (PMC4870793; doi:10.1186/s12864-016-2681-7)
Supplement: Additional file 4: Table S2. — Positively selected sites of bone-associated genes in Mammalian dataset after multiple testing correction. The alignment length is on Amino acids (aa). Gene in bold are positively selected under the comparison M2a vs M1a. Q-value estimations for multiple testing are represented as positive selected (1) and negative selected (0). (DOC 88 kb) [file 12864_2016_2681_MOESM4_ESM.doc]

# Additional file 4: Table S2 – Positively selected sites of bone-associated genes in Mammalian dataset after multiple testing correction. The alignment length is on Amino acids (aa). Gene in bold are positively selected under the comparison M2a vs M1a. Q-value estimations for multiple testing are represented as positive selected (1) and negative selected (0).

| Genes | Number of sequences | Alignment Length (aa) | Site-Class > 1 | PP >= 0.95 | q-value |
| --- | --- | --- | --- | --- | --- |
| ACVR2A | 37 | 581 | 0.027 | 0 | 0 |
| ACVR2B | 32 | 493 | 0.000 | 0 | 0 |
| ***ADAM8*** | ***29*** | ***941*** | ***0.012*** | ***5*** | ***1*** |
| ***AHSG*** | ***37*** | ***662*** | ***0.112*** | ***17*** | ***1*** |
| ANKH | 36 | 492 | 0.000 | 0 | 0 |
| ***AQP1*** | ***28*** | ***304*** | ***0.004*** | ***1*** | ***1*** |
| ASPN | 37 | 390 | 0.000 | 0 | 0 |
| BCOR | 37 | 1859 | 0.000 | 0 | 0 |
| BMP2 | 36 | 400 | 0.002 | 0 | 0 |
| BMP7 | 35 | 438 | 0.000 | 0 | 0 |
| BMPR1A | 30 | 544 | 0.000 | 0 | 0 |
| ***CA2*** | ***36*** | ***274*** | ***0.020*** | ***4*** | ***1*** |
| ***CARM1*** | ***13*** | ***658*** | ***0.018*** | ***0*** | ***1*** |
| CBS | 30 | 609 | 0.001 | 0 | 0 |
| ***CD38*** | ***37*** | ***321*** | ***0.115*** | ***5*** | ***1*** |
| CDX1 | 24 | 296 | 0.040 | 0 | 0 |
| CER1 | 33 | 293 | 0.091 | 0 | 0 |
| CITED2 | 21 | 276 | 0.000 | 0 | 0 |
| ***COL2A1*** | ***34*** | ***1521*** | ***0.009*** | ***11*** | ***1*** |
| CREB3L1 | 36 | 547 | 0.002 | 1 | 0 |
| CTHRC1 | 35 | 256 | 0.035 | 0 | 0 |
| CTSK | 37 | 353 | 0.003 | 0 | 0 |
| DLX5 | 35 | 299 | 0.012 | 0 | 0 |
| ***DUOX2*** | ***36*** | ***1634*** | ***0.016*** | ***9*** | ***1*** |
| DYM | 36 | 686 | 0.000 | 0 | 0 |
| EIF2AK3 | 39 | 1150 | 0.001 | 0 | 0 |
| ***FBXL15*** | ***29*** | ***393*** | ***0.085*** | ***5*** | ***1*** |
| FGF23 | 32 | 263 | 0.039 | 0 | 0 |
| FGF8 | 27 | 211 | 0.050 | 0 | 0 |
| ***GAS6*** | ***32*** | ***791*** | ***0.019*** | ***4*** | ***1*** |
| ***GHR*** | ***37*** | ***694*** | ***0.014*** | ***2*** | ***1*** |
| GPLD1 | 36 | 881 | 0.059 | 0 | 0 |
| ***GPM6B*** | ***35*** | ***378*** | ***0.011*** | ***1*** | ***1*** |
| ***GREM1*** | ***32*** | ***240*** | ***0.020*** | ***2*** | ***1*** |
| HOXA11 | 33 | 346 | 0.004 | 0 | 0 |
| HOXB4 | 26 | 294 | 0.045 | 0 | 0 |
| HOXD11 | 18 | 367 | 0.397 | 0 | 0 |
| ***HSD17B2*** | ***35*** | ***403*** | ***0.035*** | ***11*** | ***1*** |
| IAPP | 28 | 98 | 0.000 | 0 | 0 |
| IFITM5 | 24 | 135 | 0.024 | 0 | 0 |
| ***IGF1*** | ***28*** | ***241*** | ***0.033*** | ***3*** | ***1*** |
| IHH | 33 | 461 | 0.000 | 0 | 0 |
| ***IL6*** | ***35*** | ***317*** | ***0.107*** | ***2*** | ***1*** |
| ***IL7*** | ***14*** | ***185*** | ***0.298*** | ***8*** | ***1*** |
| INPP5D | 33 | 1262 | 0.087 | 8 | 0 |
| KLF10 | 32 | 512 | 0.002 | 1 | 0 |
| LRP6 | 38 | 1639 | 0.000 | 0 | 0 |
| LRRC17 | 37 | 458 | 0.026 | 1 | 0 |
| MC4R | 32 | 341 | 0.000 | 0 | 0 |
| ***MEF2A*** | ***38*** | ***574*** | ***0.017*** | ***8*** | ***1*** |
| ***MEF2C*** | ***34*** | ***494*** | ***0.016*** | ***8*** | ***1*** |
| ***MEPE*** | ***35*** | ***637*** | ***0.017*** | ***0*** | ***1*** |
| MGP | 35 | 129 | 0.044 | 0 | 0 |
| MITF | 36 | 533 | 0.000 | 0 | 0 |
| MMP2 | 36 | 687 | 0.001 | 0 | 0 |
| MSX1 | 31 | 320 | 0.000 | 0 | 0 |
| ***NBR1*** | ***32*** | ***1118*** | ***0.006*** | ***4*** | ***1*** |
| NCDN | 28 | 764 | 0.033 | 0 | 0 |
| NF1 | 36 | 2854 | 0.00 | 0 | 0 |
| NOX4 | 35 | 661 | 0.01 | 0 | 0 |
| ***OSR2*** | ***32*** | ***312*** | ***0.03*** | ***5*** | ***1*** |
| P2RX7 | 34 | 613 | 0.06 | 0 | 0 |
| ***PAPSS2*** | ***39*** | ***653*** | ***0.01*** | ***8*** | ***1*** |
| PKDCC | 34 | 504 | 0.02 | 0 | 0 |
| PLA2G4A | 38 | 764 | 0.00 | 0 | 0 |
| PLXNB1 | 34 | 2247 | 0.00 | 0 | 0 |
| ***PTGER4*** | ***33*** | ***546*** | ***0.01*** | ***4*** | ***1*** |
| PTH | 34 | 126 | 0.08 | 0 | 0 |
| PTK2B | 37 | 1036 | 0.00 | 0 | 0 |
| ***PTN*** | ***35*** | ***257*** | ***0.08*** | ***10*** | ***1*** |
| SBDS | 21 | 255 | 0.01 | 0 | 0 |
| SFRP1 | 31 | 329 | 0.00 | 0 | 0 |
| SFRP2 | 18 | 304 | 0.01 | 0 | 0 |
| SH3PXD2B | 29 | 939 | 0.00 | 0 | 0 |
| SPP2 | 33 | 224 | 0.07 | 0 | 0 |
| SRD5A1 | 33 | 266 | 0.01 | 1 | 0 |
| SRGN | 33 | 212 | 0.08 | 0 | 0 |
| SULF1 | 37 | 1169 | 0.00 | 0 | 0 |
| SULF2 | 31 | 929 | 0.03 | 0 | 0 |
| SYK | 36 | 665 | 0.03 | 0 | 0 |
| TCF7L2 | 37 | 697 | 0.04 | 0 | 0 |
| ***TFRC*** | ***37*** | ***807*** | ***0.05*** | ***25*** | ***1*** |
| TGFB3 | 35 | 490 | 0.00 | 0 | 0 |
| TNFAIP3 | 38 | 809 | 0.00 | 0 | 0 |
| ***TPH1*** | ***30*** | ***535*** | ***0.03*** | ***5*** | ***1*** |
| TPP1 | 33 | 589 | 0.00 | 0 | 0 |
| TRAF6 | 36 | 559 | 0.00 | 1 | 0 |
| TUFT1 | 35 | 422 | 0.10 | 0 | 0 |
| VEGFA | 32 | 484 | 0.08 | 0 | 0 |
