# Supplementary material for: Bone-associated gene evolution and the origin of flight in birds
Source: BMC Genomics. 2016 May 18;17:371. doi: 10.1186/s12864-016-2681-7 (PMC4870793; doi:10.1186/s12864-016-2681-7)
Supplement: Additional file 5: Table S3. — Positively selected sites of bone-associated genes in Avian dataset after multiple testing correction. The alignment length is on Amino acids (aa). Gene in bold are positively selected under the comparison M2a vs M1a. Q-value estimations for multiple testing are represented as positive selected (1) and negative selected (0). (DOC 88 kb) [file 12864_2016_2681_MOESM5_ESM.doc]

# Additional file 5: Table S3 – Positively selected sites of bone-associated genes in Avian dataset after multiple testing correction. The alignment length is on Amino acids (aa). Gene in bold are positively selected under the comparison M2a vs M1a. Q-value estimations for multiple testing are represented as positive selected (1) and negative selected (0).

| Genes | Number of Sequences | Alignment Length (aa) | Site-Class > 1 | PP >= 0.95 | q-value |
| --- | --- | --- | --- | --- | --- |
| ***ACVR2A*** | ***43*** | ***513*** | ***0.007*** | ***3*** | ***1*** |
| ***ACVR2B*** | ***43*** | ***516*** | ***0.015*** | ***2*** | ***1*** |
| ***ADAM8*** | ***32*** | ***531*** | ***0.014*** | ***4*** | ***1*** |
| ***AHSG*** | ***43*** | ***384*** | ***0.118*** | ***16*** | ***1*** |
| ***ANKH*** | ***44*** | ***509*** | ***0.020*** | ***6*** | ***1*** |
| AQP1 | 44 | 271 | 0.000 | 0 | 0 |
| ***ASPN*** | ***40*** | ***641*** | ***0.011*** | ***6*** | ***1*** |
| BCOR | 29 | 1798 | 0.002 | 0 | 0 |
| ***BMP2*** | ***45*** | ***367*** | ***0.015*** | ***4*** | ***1*** |
| BMP7 | 44 | 425 | 0.000 | 0 | 0 |
| ***BMPR1A*** | ***42*** | ***541*** | ***0.018*** | ***7*** | ***1*** |
| CA2 | 45 | 271 | 0.031 | 0 | 0 |
| CARM1 | 19 | 412 | 0.044 | 0 | 0 |
| ***CBS*** | ***42*** | ***94*** | ***0.014*** | ***1*** | ***1*** |
| ***CD38*** | ***44*** | ***312*** | ***0.036*** | ***3*** | ***1*** |
| CDX1 | 30 | 255 | 0.000 | 0 | 0 |
| ***CER1*** | ***45*** | ***273*** | ***0.033*** | ***3*** | ***1*** |
| ***CITED2*** | ***16*** | ***293*** | ***0.031*** | ***1*** | ***1*** |
| COL2A1 | 19 | 268 | 0.038 | 0 | 0 |
| ***CREB3L1*** | ***43*** | ***520*** | ***0.044*** | ***13*** | ***1*** |
| ***CTHRC1*** | ***45*** | ***244*** | ***0.071*** | ***2*** | ***1*** |
| CTSK | 19 | 132 | 0.132 | 0 | 0 |
| ***DLX5*** | ***16*** | ***316*** | ***0.135*** | ***21*** | ***1*** |
| ***DUOX2*** | ***43*** | ***1623*** | ***0.014*** | ***15*** | ***1*** |
| DYM | 24 | 680 | 0.009 | 0 | 0 |
| EIF2AK3 | 44 | 1012 | 0.000 | 0 | 0 |
| ***FBXL15*** | ***44*** | ***297*** | ***0.003*** | ***1*** | ***1*** |
| ***FGF23*** | ***45*** | ***275*** | ***0.026*** | ***5*** | ***1*** |
| FGF8 | 36 | 229 | 0.021 | 0 | 0 |
| ***GAS6*** | ***44*** | ***686*** | ***0.020*** | ***4*** | ***1*** |
| ***GHR*** | ***42*** | ***619*** | ***0.022*** | ***5*** | ***1*** |
| ***GPLD1*** | ***44*** | ***856*** | ***0.029*** | ***8*** | ***1*** |
| GPM6B | 45 | 328 | 0.000 | 0 | 0 |
| GREM1 | 44 | 186 | 0.005 | 1 | 0 |
| ***HOXA11*** | ***29*** | ***310*** | ***0.085*** | ***8*** | ***1*** |
| ***HOXB4*** | ***41*** | ***56*** | ***0.070*** | ***3*** | ***1*** |
| HOXD11 | 44 | 284 | 0.033 | 0 | 0 |
| ***HSD17B2*** | ***40*** | ***388*** | ***0.029*** | ***2*** | ***1*** |
| ***IAPP*** | ***45*** | ***136*** | ***0.016*** | ***1*** | ***1*** |
| IFITM5 | 16 | 151 | 0.068 | 0 | 0 |
| ***IGF1*** | ***45*** | ***153*** | ***0.007*** | ***1*** | ***1*** |
| IHH | 22 | 312 | 0.041 | 0 | 0 |
| ***IL6*** | ***42*** | ***169*** | ***0.034*** | ***2*** | ***1*** |
| IL7 | 44 | 145 | 0.029 | 0 | 0 |
| INPP5D | 23 | 1227 | 0.008 | 0 | 0 |
| KLF10 | 42 | 485 | 0.011 | 0 | 0 |
| LRP6 | 45 | 1618 | 0.000 | 0 | 0 |
| ***LRRC17*** | ***45*** | ***442*** | ***0.006*** | ***2*** | ***1*** |
| MC4R | 45 | 331 | 0.003 | 0 | 0 |
| MEF2A | 44 | 527 | 0.019 | 0 | 0 |
| ***MEF2C*** | ***43*** | ***478*** | ***0.028*** | ***8*** | ***1*** |
| ***MEPE*** | ***35*** | ***82*** | ***0.144*** | ***7*** | ***1*** |
| ***MGP*** | ***44*** | ***104*** | ***0.070*** | ***3*** | ***1*** |
| MITF | 45 | 468 | 0.013 | 0 | 0 |
| MMP2 | 43 | 672 | 0.000 | 0 | 0 |
| MSX1 | 31 | 294 | 0.104 | 0 | 0 |
| ***NBR1*** | ***41*** | ***1081*** | ***0.031*** | ***26*** | ***1*** |
| ***NCDN*** | ***44*** | ***772*** | ***0.038*** | ***6*** | ***1*** |
| NF1 | 41 | 2834 | 0.000 | 0 | 0 |
| ***NOX4*** | ***41*** | ***597*** | ***0.005*** | ***2*** | ***1*** |
| ***OSR2*** | ***27*** | ***316*** | ***0.026*** | ***4*** | ***1*** |
| ***P2RX7*** | ***11*** | ***598*** | ***0.155*** | ***22*** | ***1*** |
| PAPSS2 | 42 | 626 | 0.001 | 0 | 0 |
| ***PKDCC*** | ***40*** | ***296*** | ***0.035*** | ***5*** | ***1*** |
| PLA2G4A | 37 | 772 | 0.005 | 0 | 0 |
| ***PLXNB1*** | ***42*** | ***2250*** | ***0.011*** | ***10*** | ***1*** |
| ***PTGER4*** | ***43*** | ***478*** | ***0.014*** | ***1*** | ***1*** |
| PTH | 45 | 119 | 0.010 | 0 | 0 |
| PTK2B | 14 | 1127 | 0.059 | 0 | 0 |
| PTN | 43 | 165 | 0.007 | 0 | 0 |
| SBDS | 45 | 172 | 0.000 | 0 | 0 |
| SFRP1 | 9 | 314 | 0.010 | 0 | 0 |
| SFRP2 | 22 | 313 | 0.037 | 0 | 0 |
| SH3PXD2B | 41 | 927 | 0.048 | 1 | 0 |
| SPP2 | 44 | 193 | 0.043 | 0 | 0 |
| ***SRD5A1*** | ***42*** | ***177*** | ***0.041*** | ***2*** | ***1*** |
| SRGN | 44 | 148 | 0.022 | 0 | 0 |
| ***SULF1*** | ***40*** | ***892*** | ***0.009*** | ***6*** | ***1*** |
| SULF2 | 45 | 906 | 0.002 | 1 | 0 |
| ***SYK*** | ***36*** | ***655*** | ***0.009*** | ***3*** | ***1*** |
| ***TCF7L2*** | ***45*** | ***511*** | ***0.031*** | ***3*** | ***1*** |
| ***TFRC*** | ***45*** | ***791*** | ***0.071*** | ***33*** | ***1*** |
| TGFB3 | 45 | 296 | 0.003 | 0 | 0 |
| ***TNFAIP3*** | ***44*** | ***833*** | ***0.013*** | ***7*** | ***1*** |
| TPH1 | 43 | 444 | 0.021 | 0 | 0 |
| ***TPP1*** | ***21*** | ***480*** | ***0.314*** | ***95*** | ***1*** |
| TRAF6 | 44 | 545 | 0.000 | 0 | 0 |
| TUFT1 | 38 | 394 | 0.000 | 0 | 0 |
| ***VEGFA*** | ***44*** | ***239*** | ***0.061*** | ***8*** | ***1*** |
