# Supplementary material for: Bone-associated gene evolution and the origin of flight in birds
Source: BMC Genomics. 2016 May 18;17:371. doi: 10.1186/s12864-016-2681-7 (PMC4870793; doi:10.1186/s12864-016-2681-7)
Supplement: Additional file 6: Table S4. — Positively selected sites of bone-associated genes in Reptilian dataset after multiple testing correction. The alignment length is on Amino acids (aa). Bold represents statistical significance (p < 0.05). Q-value estimations for multiple testing are represented as positive selected (1) and negative selected (0). (DOC 158 kb) [file 12864_2016_2681_MOESM6_ESM.doc]

# Additional file 6: Table S4 – Positively selected sites of bone-associated genes in Reptilian dataset after multiple testing correction. The alignment length is on Amino acids (aa). Bold represents statistical significance (p<0.05). Q-value estimations for multiple testing are represented as positive selected (1) and negative selected (0).

| Genes | Sequences  Number | Alignment Length (aa) | Model 0  (*lnL*) | Omega (ω) | Model 1 (*lnL*) | Model 2 (*lnL*) | 2∆L | p-value | q-value |
| --- | --- | --- | --- | --- | --- | --- | --- | --- | --- |
| *ACVR2A* | 8 | 522 | -3132.02 | 0.02834 | -3129.25 | -3129.25 | 0.00 | 1.00 | 0 |
| ***ACVR2B*** | **8** | **628** | **-5969.82** | **0.1119** | **-5794.83** | **-5788.7** | **12.25** | **1.00** | **1** |
| ***ADAM8*** | **5** | **1161** | **-8876.42** | **0.2817** | **-8762.88** | **-8681.27** | **163.21** | **1.00** | **1** |
| ***AHSG*** | **5** | **384** | **-4188.35** | **0.49727** | **-4144.94** | **-4139.99** | **9.89** | **1.00** | **1** |
| *ANKH* | 8 | 495 | -4073.78 | 0.03151 | -4063.7 | -4063.7 | 0.00 | 1.00 | 0 |
| *AQP1* | 7 | 271 | -2957.95 | 0.07548 | -2917.92 | -2917.92 | 0.00 | 1.00 | 0 |
| *ASPN* | 8 | 383 | -3847.76 | 0.12594 | -3818.89 | -3818.89 | 0.00 | 1.00 | 0 |
| *BCOR* | 7 | 1866 | -22475.7 | 0.17843 | -22273.3 | -22273.3 | 0.00 | 1.00 | 0 |
| *BMP2* | 12 | 400 | -4671.38 | 0.1259 | -4654.09 | -4654.09 | 0.00 | 1.00 | 0 |
| *BMP7* | 7 | 815 | -6238.17 | 0.16286 | -6137.83 | -6137.73 | 0.20 | 1.00 | 0 |
| ***BMPR1A*** | **8** | **536** | **-4980.97** | **2.4755** | **-5017.62** | **-4968.89** | **97.46** | **1.00** | **1** |
| *CA2* | 8 | 266 | -3304.43 | 0.27874 | -3239.45 | -3239.45 | 0.00 | 1.00 | 0 |
| ***CARM1*** | **8** | **685** | **-7043.78** | **0.07318** | **-6813.02** | **-6804.18** | **17.68** | **1.00** | **1** |
| *CBS* | 7 | 594 | -7161.99 | 0.18867 | -6974.43 | -6974.43 | 0.00 | 1.00 | 0 |
| *CD38* | 8 | 361 | -4331.51 | 0.25497 | -4268.77 | -4268.77 | 0.00 | 1.00 | 0 |
| *CDX1* | 8 | 384 | -5959.32 | 0.14693 | -5883.42 | -5883.42 | 0.00 | 1.00 | 0 |
| *CER1* | 8 | 284 | -4434.96 | 0.44558 | -4371.04 | -4371.04 | 0.00 | 1.00 | 0 |
| *CITED2* | 8 | 256 | -2638.6 | 0.092 | -2599.26 | -2599.26 | 0.00 | 1.00 | 0 |
| *COL2A1* | 8 | 1920 | -17736.3 | 0.1307 | -17606.9 | -17606.9 | 0.00 | 1.00 | 0 |
| *CREB3L1* | 8 | 466 | -4517.51 | 0.14326 | -4460.68 | -4460.68 | 0.00 | 1.00 | 0 |
| ***CTHRC1*** | **8** | **463** | **-4539.8** | **0.27087** | **-4280.87** | **-4243.07** | **75.61** | **1.00** | **1** |
| *CTSK* | 5 | 336 | -2949.59 | 0.05615 | -2921.84 | -2921.84 | 0.00 | 1.00 | 0 |
| *DLX5* | 8 | 305 | -3399.93 | 0.11174 | -3377.11 | -3377.11 | 0.00 | 1.00 | 0 |
| ***DUOX2*** | **8** | **1550** | **-19016.5** | **0.15126** | **-18610.4** | **-18602.4** | **15.87** | **1.00** | **1** |
| *DYM* | 8 | 673 | -5623.61 | 0.0673 | -5607.57 | -5607.57 | 0.00 | 1.00 | 0 |
| *EIF2AK3* | 9 | 1411 | -14360.6 | 0.16437 | -14188 | -14188 | 0.00 | 1.00 | 0 |
| ***FBXL15*** | **8** | **307** | **-3829.15** | **0.13541** | **-3746.23** | **-3722.06** | **48.34** | **1.00** | **1** |
| *FGF23* | 7 | 256 | -2935.26 | 0.17442 | -2904.01 | -2904.01 | 0.00 | 1.00 | 0 |
| *FGF8* | 8 | 219 | -2119.79 | 0.06086 | -2084.84 | -2084.84 | 0.00 | 1.00 | 0 |
| ***GAS6*** | **8** | **709** | **-8627.08** | **0.26301** | **-8422.49** | **-8294.65** | **255.68** | **1.00** | **1** |
| *GHR* | 14 | 629 | -9202.52 | 0.32056 | -9082.73 | -9082.73 | 0.00 | 1.00 | 0 |
| *GPLD1* | 8 | 840 | -9851.38 | 0.26727 | -9775.27 | -9775.27 | 0.00 | 1.00 | 0 |
| *GPM6B* | 8 | 300 | -2999.38 | 0.10741 | -2975.19 | -2975.19 | 0.00 | 1.00 | 0 |
| *GREM1* | 8 | 186 | -1943.33 | 0.11647 | -1917.06 | -1917.06 | 0.00 | 1.00 | 0 |
| *HOXA11* | 8 | 358 | -3070.89 | 0.1148 | -3041.57 | -3041.57 | 0.00 | 1.00 | 0 |
| *HOXB4* | 8 | 261 | -2400.51 | 0.1197 | -2384.97 | -2384.97 | 0.00 | 1.00 | 0 |
| *HOXD11* | 8 | 316 | -3412.58 | 0.17217 | -3365.02 | -3365.02 | 0.00 | 1.00 | 0 |
| *HSD17B2* | 6 | 394 | -4446.54 | 0.32252 | -4387.04 | -4387.04 | 0.00 | 1.00 | 0 |
| *IAPP* | 7 | 138 | -1387.18 | 0.33618 | -1360.04 | -1359.65 | 0.78 | 1.00 | 0 |
| *IFITM5* | 8 | 148 | -1653.57 | 0.16805 | -1653.31 | -1653.31 | 0.00 | 1.00 | 0 |
| *IGF* | 7 | 138 | -1192.01 | 0.15573 | -1184.7 | -1184.7 | 0.00 | 1.00 | 0 |
| *IHH* | 4 | 412 | -3380.09 | 0.10193 | -3345.08 | -3345.08 | 0.00 | 1.00 | 0 |
| ***IL6*** | **8** | **342** | **-4413.28** | **0.41056** | **-4354.58** | **-4309.37** | **90.43** | **1.00** | **1** |
| ***IL7*** | **3** | **200** | **-1298.55** | **0.83795** | **-1287.79** | **-1275.13** | **25.33** | **1.00** | **1** |
| *INPP5D* | 8 | 1224 | -13564.9 | 0.21157 | -13363.9 | -13363.9 | 0.00 | 1.00 | 0 |
| ***KLF10*** | **8** | **613** | **-6993.99** | **0.31306** | **-6849.78** | **-6844.75** | **10.06** | **1.00** | **1** |
| ***LRP6*** | **8** | **1623** | **-15598.9** | **0.06071** | **-15502.8** | **-15472** | **61.65** | **1.00** | **1** |
| *LRRC17* | 9 | 442 | -5441.1 | 0.17674 | -5391.16 | -5390.41 | 1.49 | 1.00 | 0 |
| *MC4R* | 8 | 334 | -3401.71 | 0.06888 | -3381.61 | -3381.61 | 0.00 | 1.00 | 0 |
| *MEF2A* | 8 | 539 | -4518.68 | 0.06365 | -4507.61 | -4507.61 | 0.00 | 1.00 | 0 |
| *MEF2C* | 8 | 495 | -3427.73 | 0.08718 | -3365.31 | -3363.69 | 3.25 | 1.00 | 0 |
| *MEPE* | 1 | 1013 | # | # | # | # | # | # | # |
| *MGP* | 8 | 106 | -1333.18 | 0.25092 | -1308.47 | -1308.47 | 0.00 | 1.00 | 0 |
| ***MITF*** | **8** | **684** | **-8344.85** | **0.22057** | **-7953.96** | **-7823.26** | **261.40** | **1.00** | **1** |
| *MMP2* | 7 | 682 | -5992.16 | 0.08075 | -5958.68 | -5958.68 | 0.00 | 1.00 | 0 |
| *MSX1* | 6 | 313 | -3055.72 | 0.09735 | -3026.64 | -3026.64 | 0.00 | 1.00 | 0 |
| *NBR1* | 8 | 1053 | -13291.1 | 0.28548 | -13169.7 | -13169.7 | 0.00 | 1.00 | 0 |
| *NCDN* | 8 | 724 | -9385.05 | 0.21917 | -9288.97 | -9288.97 | 0.00 | 1.00 | 0 |
| ***NF1*** | **8** | **2791** | **-20611.4** | **0.03561** | **-20553.5** | **-20545.6** | **15.81** | **1.00** | **1** |
| *NOX4* | 7 | 474 | -4473.47 | 0.16332 | -4447.81 | -4447.81 | 0.00 | 1.00 | 0 |
| *OSR2* | 7 | 354 | -2974.04 | 0.06859 | -2949.05 | -2949.05 | 0.00 | 1.00 | 0 |
| *P2RX7* | 7 | 625 | -6925.81 | 0.24868 | -6833.48 | -6833.48 | 0.00 | 1.00 | 0 |
| ***PAPSS2*** | **7** | **653** | **-5887.61** | **0.10549** | **-5798.68** | **-5781.56** | **34.24** | **1.00** | **1** |
| *PKDCC* | 8 | 544 | -6160.02 | 0.2163 | -6068 | -6067.22 | 1.57 | 1.00 | 0 |
| *PLA2G4A* | 8 | 999 | -9441.11 | 0.09882 | -9374.26 | -9374.26 | 0.00 | 1.00 | 0 |
| *PLXNB1* | 8 | 1896 | -20070.8 | 0.14978 | -19918.7 | -19918.7 | 0.00 | 1.00 | 0 |
| *PTGER4* | 9 | 476 | -5655.77 | 0.09063 | -5537.75 | -5534.34 | 6.81 | 1.00 | 0 |
| *PTH* | 6 | 129 | -1227.83 | 0.25245 | -1226.64 | -1226.64 | 0.00 | 1.00 | 0 |
| *PTK2B* | 8 | 1020 | -10231.8 | 0.05955 | -10182.3 | -10182.3 | 0.00 | 1.00 | 0 |
| *PTN* | 7 | 166 | -1483 | 0.12399 | -1478.28 | -1478.28 | 0.00 | 1.00 | 0 |
| *SBDS* | 7 | 251 | -2231.47 | 0.06189 | -2214.24 | -2214.24 | 0.00 | 1.00 | 0 |
| *SFRP1* | 9 | 348 | -4234.05 | 0.06652 | -4120.09 | -4120.09 | 0.00 | 1.00 | 0 |
| *SFRP2* | 8 | 338 | -2940.23 | 0.07075 | -2922.7 | -2922.7 | 0.00 | 1.00 | 0 |
| *SH3PXD2B* | 8 | 978 | -10839.8 | 0.20149 | -10738.7 | -10738.7 | 0.00 | 1.00 | 0 |
| *SPP2* | 4 | 193 | -1236.17 | 0.46943 | -1231.14 | -1227.86 | 6.55 | 1.00 | 0 |
| ***SRD5A1*** | **7** | **290** | **-3516.7** | **0.46147** | **-3428.99** | **-3417.08** | **23.82** | **1.00** | **1** |
| *SRGN* | 8 | 154 | -2102.17 | 0.4595 | -2089.77 | -2089.71 | 0.11 | 1.00 | 0 |
| *SULF1* | 8 | 890 | -7360.44 | 0.0871 | -7287.9 | -7287.9 | 0.00 | 1.00 | 0 |
| *SULF2* | 7 | 895 | -7346.82 | 0.0691 | -7284.16 | -7284.16 | 0.00 | 1.00 | 0 |
| ***SYK*** | **9** | **658** | **-8070.84** | **0.1646** | **-7888.45** | **-7878.35** | **20.21** | **1.00** | **1** |
| *TCF7L2* | 8 | 636 | -4552.25 | 0.07776 | -4515.75 | -4515.75 | 0.00 | 1.00 | 0 |
| ***TFRC*** | **6** | **1104** | **-11379.8** | **0.42277** | **-11202.6** | **-11180.3** | **44.53** | **1.00** | **1** |
| *TGFB3* | 8 | 420 | -4377.43 | 0.0802 | -4353.08 | -4353.08 | 0.00 | 1.00 | 0 |
| *TNFAIP3* | 8 | 819 | -9901.49 | 0.22989 | -9811.71 | -9811.71 | 0.00 | 1.00 | 0 |
| *TPH1* | 7 | 495 | -4527.83 | 0.10665 | -4492.34 | -4492.34 | 0.00 | 1.00 | 0 |
| *TPP1* | 8 | 600 | -7445.56 | 0.13874 | -7255.63 | -7255.63 | 0.00 | 1.00 | 0 |
| *TRAF6* | 8 | 550 | -5800.74 | 0.16633 | -5723.12 | -5723.12 | 0.00 | 1.00 | 0 |
| ***TUFT1*** | **8** | **461** | **-5990.37** | **0.15883** | **-5863.78** | **-5835.93** | **55.69** | **1.00** | **1** |
| *VEGFA* | 8 | 223 | -1977.64 | 0.24717 | -1962.27 | -1962.17 | 0.21 | 1.00 | 0 |
