# Supplementary material for: Bone-associated gene evolution and the origin of flight in birds
Source: BMC Genomics. 2016 May 18;17:371. doi: 10.1186/s12864-016-2681-7 (PMC4870793; doi:10.1186/s12864-016-2681-7)
Supplement: Additional file 7: Figure S3. — Venn diagrams of positively-selected bone-associated genes. The intersection between the positively selected genes in the three clades, birds, mammals and reptiles. (DOC 149 kb) [file 12864_2016_2681_MOESM7_ESM.doc]

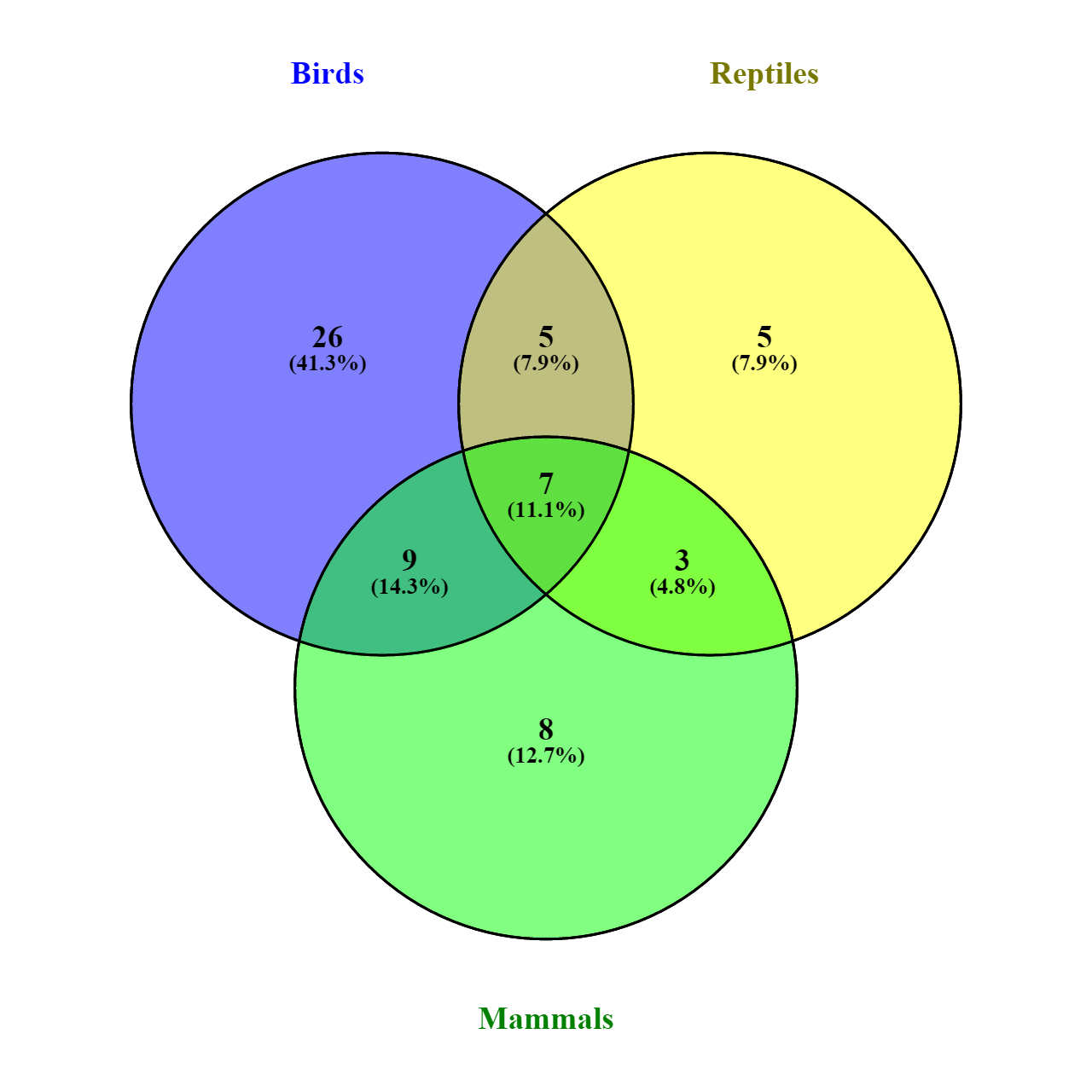


# Additional file 7: Figure S3 - Venn diagrams of positively-selected bone-associated genes. The intersection between the positively selected genes in the three clades, birds, mammals and reptiles.
