# Supplementary material for: Bone-associated gene evolution and the origin of flight in birds
Source: BMC Genomics. 2016 May 18;17:371. doi: 10.1186/s12864-016-2681-7 (PMC4870793; doi:10.1186/s12864-016-2681-7)
Supplement: Additional file 8: Table S5. — Branch-site model for birds. Genes were the alternate model was preferred relatively to the null model are highlighted as italic and underlined positively selected. (DOC 141 kb) [file 12864_2016_2681_MOESM8_ESM.doc]

# Additional file 8: Table S5 - Branch-site model for birds. Genes were the alternate model was preferred relatively to the null model are highlighted as italic and underlined positively selected.

| **Gene** | ***Lnl Alternate Model*** | ***Lnl Null Model*** | **LRT** | **p-value** | **q-value** | **Background (2a/2b)** | **Foreground (2a/2b)** | **Sites** |
| --- | --- | --- | --- | --- | --- | --- | --- | --- |
| ***ACVR2A*** | -11466.7 | -11466.7 | 0.00 | 1.00 | 0 | 0.00472/1.00000 | 1.00000/1.00000 | 37(0.719) 38(0.867) 52(0.592) |
| ***ACVR2B*** | *-8537.79* | *-8549.76* | *23.95* | *0.00* | *1* | *0.00761/1.00000* | *9.43172/9.43172* | *9(0.977) 10(0.991) 11(1.000) 12(0.989) 13(0.696)* |
| ***ADAM8*** | -18545.4 | -18545.4 | 0.00 | 1.00 | 0 | 0.09300/1.00000 | 1.00000/1.00000 | 205(0.880) 208(0.672) 215(0.588) 229(1.000) 230(1.000) |
| ***AHSG*** | *-13597.1* | *-13698* | *201.62* | *0.00* | *1* | *0.09594/1.00000* | *4.52672/4.52672* | *372(0.987) 408(0.965) 409(0.979) 410(1.000) 415(0.965)*  *416(0.879) 417(0.903) 418(1.000) 422(1.000) 424(0.871)*  *471(1.000) 481(1.000) 521(0.547) 522(0.761) 523(0.764)*  *524(0.871) 526(0.990) 527(0.994) 544(0.999) 577(1.000)* |
| ***ANKH*** | *-7804.21* | *-7813.08* | *17.75* | *0.00* | *1* | *0.00520/1.00000* | *8.41713/8.41713* | *24(1.000) 27(0.528) 30(0.960) 287(0.903)* |
| ***AQP1*** | -5702.63 | -5702.63 | 0.00 | 1.00 | 0 | 0.01721/1.00000 | 1.00000/1.00000 | 129(0.716) |
| ***ASPN*** | -14507.1 | -14507.1 | 0.00 | 1.00 | 0 | 0.04814/1.00000 | 1.00000/1.00000 | 371(1.000) 372(1.000) 373(0.998) 374(1.000) 491(0.982) |
| ***BCOR*** | -32214.2 | -32214.2 | 0.00 | 1.00 | 0 | 0.05424/1.00000 | 1.00000/1.00000 | 136(0.776) 301(0.729) 338(0.526) 357(0.702) 363(0.590)  428(0.642) 433(0.737) 435(0.645) 470(0.539) 598(0.522)  645(0.591) 682(0.671) 844(0.784) 909(0.560) 972(0.887)  979(0.842) 1019(0.744) 1174(0.522) 1493(0.535) 1494(0.518)  1501(0.884) 1524(0.603) 1538(0.671) 1574(0.766) 1591(0.549)  1615(0.678) |
| ***BMP2*** | *-3520.53* | *-3536.47* | *31.88* | *0.00* | *1* | *0.01599/1.00000* | *999.00000/999.00000* | *47(0.930) 48(0.906) 49(0.930) 51(0.891) 52(0.756) 99(0.872)*  *116(0.721) 120(0.874) 122(0.968)* |
| ***BMP7*** | -6478.92 | -6478.92 | 0.00 | 1.00 | 0 | 0.01303/1.00000 | 1.00000/1.00000 |  |
| ***BMPR1A*** | *-9069.35* | *-9105.89* | *73.08* | *0.00* | *1* | *0.01171/1.00000* | *7.83816/7.83816* | *16(1.000) 20(1.000) 21(0.748) 22(0.937) 23(1.000) 24(0.997)*  *29(0.943)* |
| ***CA2*** | -6449.54 | -6449.54 | 0.00 | 1.00 | 0 | 0.04152/1.00000 | 1.00000/1.00000 | 185(0.758) 186(0.841) 187(0.771) 189(0.521) |
| ***CARM1*** | -6548.46 | -6548.46 | 0.00 | 1.00 | 0 | 0.08848/1.00000 | 1.00000/1.00000 | 353(0.697) 365(0.514) |
| ***CBS*** | -2714.43 | -2714.43 | 0.00 | 1.00 | 0 | 0.08847/1.00000 | 1.00000/1.00000 |  |
| ***CD38*** | -8492.06 | -8492.06 | 0.00 | 1.00 | 0 | 0.07701/1.00000 | 1.00000/1.00000 | 52(0.807) 54(0.989) 80(0.929) |
| ***CDX1*** | -4516.18 | -4516.18 | 0.00 | 1.00 | 0 | 0.05379/1.00000 | 6.60339/6.60339 |  |
| ***CER1*** | *-9797.38* | *-9803.05* | *11.34* | *0.00* | *1* | *0.14337/1.00000* | *2.77020/2.77020* | *45(0.624) 58(0.700) 127(0.527) 146(0.988) 197(0.768) 273(0.729)*  *277(0.589)* |
| ***CITED2*** | -2500.25 | -2500.25 | 0.00 | 1.00 | 0 | 0.06211/1.00000 | 1.00000/1.00000 | 178(0.536) 182(0.577) 183(0.647) 186(0.737) 187(0.591) 189(0.793)  213(0.937) 214(0.946) 215(0.671) 216(0.956) |
| ***COL2A1*** | -5058.69 | -5061.7 | 0.00 | 1.00 | 0 | 0.00890/1.00000 | 3.15776/3.15776 | 1414(0.947) 1417(0.630) 1418(0.797) 1419(0.980) 1421(0.983) |
| ***CREB3L1*** | *-9588.33* | *-9626.66* | *76.66* | *0.00* | *1* | *0.04219/1.00000* | *5.35426/5.35426* | *24(0.621) 25(0.595) 28(0.722) 30(1.000) 31(0.965) 32(0.987) 33(1.000)*  *34(1.000) 36(0.997) 45(0.970) 47(0.904) 122(0.729) 524(0.729)* |
| ***CTHRC1*** | -5041.66 | -5041.66 | 0.00 | 1.00 | 0 | 0.00634/1.00000 | 1.00000/1.00000 |  |
| ***CTSK*** | -2973.05 | -2973.05 | 0.00 | 1.00 | 0 | 0.02778/1.00000 | 1.00000/1.00000 |  |
| ***DLX5*** | *-4990.85* | *-5063.66* | *145.62* | *0.00* | *1* | *0.03903/1.00000* | *7.50722/7.50722* | *146(0.981) 147(1.000) 148(0.997) 149(0.998) 151(1.000) 152(0.920)*  *154(0.992) 155(0.997) 157(0.997) 158(0.973) 159(0.988) 160(0.984)*  *161(0.997) 162(1.000) 163(0.961) 165(0.882) 166(0.503) 167(0.958)*  *168(0.956) 169(0.892) 170(0.654) 172(0.994) 173(0.972) 174(1.000)*  *175(0.727) 177(1.000) 179(0.988) 180(0.916) 181(1.000) 182(0.998)*  *183(0.999) 184(0.997) 185(0.998) 186(0.996) 194(0.996) 196(0.505)*  *197(1.000) 203(0.971)* |
| ***DUOX2*** | -52149.7 | -52149.7 | 0.00 | 1.00 | 0 | 0.06437/1.00000 | 1.00000/1.00000 | 282(1.000) 319(0.886) 376(0.686) 791(0.992) 1016(0.980)  1017(1.000)  1018(1.000) 1019(1.000) 1029(1.000) 1066(0.965) 1076(0.528)  1119(1.000) |
| ***DYM*** | -12133.3 | -12127.1 | 0.00 | 1.00 | 0 | 0.03220/1.00000 | 9.10760/9.10760 | 683(0.552) 691(0.571) |
| ***EIF2AK3*** | -23482.9 | -23482.9 | 0.00 | 1.00 | 0 | 0.04858/1.00000 | 1.00000/1.00000 |  |
| ***FBXL15*** | *-6847.77* | *-6859.32* | *23.09* | *0.00* | *1* | *0.03721/1.00000* | *8.12445/8.12445* |  |
| ***FGF23*** | *-7815.47* | *-7830.56* | *30.18* | *0.00* | *1* | *0.06872/1.00000* | *6.30871/6.30871* | *11(0.979) 12(0.858) 14(1.000) 15(0.996) 16(0.989) 17(0.967)*  *18(0.945) 20(0.847) 230(0.990)* |
| ***FGF8*** | -3523.45 | -3523.45 | 0.00 | 1.00 | 0 | 0.03162/1.00000 | 1.00000/1.00000 | 3(0.550) 4(0.753) 6(0.845) 11(0.856) |
| ***GAS6*** | -16401.1 | -16401.1 | 0.00 | 1.00 | 0 | 0.06549/1.00000 | 1.00000/1.00000 |  |
| ***GHR*** | *-15534* | *-15552.6* | *37.21* | *0.00* | *1* | *0.08702/1.00000* | *4.73808/4.73808* | *33(1.000) 34(1.000) 294(0.985) 498(1.000) 516(0.907) 565(0.821)* |
| ***GPLD1*** | -25587.4 | -25587.4 | 0.00 | 1.00 | 0 | 0.08697/1.00000 | 1.00000/1.00000 | 81(0.985) 85(0.744) 335(0.754) 375(0.924) 641(0.992) 684(1.000)  830(0.994) |
| ***GPM6B*** | -4067.28 | -4066.95 | 0.00 | 1.00 | 0 | 0.01406/1.00000 | 16.794351/6.79435 |  |
| ***GREM1*** | -3827.51 | -3827.51 | 0.00 | 1.00 | 0 | 0.01340/1.00000 | 1.00000/1.00000 |  |
| ***HOXA11*** | *-3674.04* | *-3704.1* | *60.10* | *0.00* | *1* | *0.04082/1.00000* | *6.21140/6.21140* | *176(0.873) 178(0.996) 179(0.858) 180(0.743) 181(0.991) 182(0.982)*  *183(0.998) 184(0.998) 185(0.777) 186(0.948) 215(0.785) 216(0.564)*  *217(0.712) 218(0.957) 219(0.740) 220(0.973) 221(0.893) 223(0.931)*  *224(0.941) 225(0.998) 226(0.818) 227(0.983) 228(1.000) 229(0.993)*  *230(0.998) 231(0.988) 232(0.999) 233(0.999) 234(0.886) 235(1.000)*  *236(1.000) 237(0.995) 238(0.960) 240(0.930) 241(0.942) 242(0.593)*  *243(0.991) 244(0.944) 246(0.885) 247(0.807) 248(0.991) 251(0.995)*  *252(0.849) 253(0.988) 255(0.906) 256(0.999) 257(0.997) 260(0.752)*  *261(0.972) 262(0.536) 263(0.838)* |
| ***HOXB4*** | -627.896 | -627.896 | 0.00 | 1.00 | 0 | 0.03335/1.00000 | 1.00000/1.00000 | 97(0.833) 100(0.822) |
| ***HOXD11*** | -3730.09 | -3730.09 | 0.00 | 1.00 | 0 | 0.04878/1.00000 | 1.00000/1.00000 |  |
| ***HSD17B2*** | *-10542.3* | *-10547.7* | *10.86* | *0.00* | *1* | *0.08753/1.00000* | *2.90265/2.90265* | *319(0.946) 322(0.715) 333(0.598) 357(0.997)* |
| ***IAPP*** | -3739.34 | -3739.34 | 0.00 | 1.00 | 0 | 0.13650/1.00000 | 1.00000/1.00000 | 42(0.592) 53(0.614) 61(0.697) 69(0.689) 73(0.721) 130(0.561) |
| ***IFITM5*** | -3346.17 | -3346.17 | 0.00 | 1.00 | 0 | 0.06518/1.00000 | 1.00000/1.00000 | 16(0.811) 21(0.773) 23(0.534) 66(0.599) 73(0.692) 75(0.988)  76(0.903) 77(0.653) 78(0.998) 79(0.790) 81(0.995) 82(0.912)  83(0.962) 84(0.892) 85(0.968) 86(0.992) 87(0.778) 88(1.000)  89(0.965) 90(0.885) 91(0.985) 92(0.694) 93(0.991) 95(0.796)  96(0.814) 97(0.779) 99(0.971) 101(0.975) 114(0.957) 115(0.712)  119(0.947) 151(0.976) |
| ***IGF1*** | -1450.58 | -1450.58 | 0.00 | 1.00 | 0 | 0.03761/1.00000 | 1.00000/1.00000 |  |
| ***IHH*** | -6412.46 | -6412.46 | 0.00 | 1.00 | 0 | 0.05739/1.00000 | 1.00000/1.00000 | 309(0.939) 316(0.874) 349(0.880) 357(0.928) 360(0.805)  362(0.567) 368(0.889) 373(0.606) 377(0.537) 379(0.855)  390(0.701) 457(0.766) |
| ***IL6*** | *-5560.4* | *-5568.99* | *17.18* | *0.00* | *1* | *0.11161/1.00000* | *4.13380/4.13380* | *168(0.846) 170(0.956) 171(0.796) 172(0.605) 173(0.902)* |
| ***IL7*** | -3401.38 | -3401.38 | 0.00 | 1.00 | 0 | 0.14012/1.00000 | 1.00000/1.00000 |  |
| ***INPP5D*** | -18486.7 | -18486.7 | 0.00 | 1.00 | 0 | 0.03505/1.00000 | 1.00000/1.00000 | 156(0.762) 158(0.650) 276(0.552) 624(0.507) 974(0.537)  1125(0.552) |
| ***KLF10*** | -17544.5 | -17544.5 | 0.00 | 1.00 | 0 | 0.07517/1.00000 | 1.00000/1.00000 | 233(0.777) 285(0.798) |
| ***LRP6*** | -30326.36 | -30326.36 | 0.00 | 1.00 | 0 | 0.01665/1.00000 | 1.00000/1.00000 |  |
| ***LRRC17*** | *-9904.79* | *-9912.48* | *15.38* | *0.00* | *1* | *0.07225/1.00000* | *7.87680/7.87680* |  |
| ***MC4R*** | -6077.29 | -6077.29 | 0.00 | 1.00 | 0 | 0.01817/1.00000 | 1.00000/1.00000 | 12(0.703) 17(0.706) 21(0.671) 31(0.799) 32(0.696) 333(0.678) |
| ***MEF2A*** | -10652 | -10652 | 0.00 | 1.00 | 0 | 0.02686/1.00000 | 1.00000/1.00000 | 247(0.541) 274(0.895) 305(0.727) 415(0.578) 498(0.523)  503(0.546) 522(0.715) 523(0.767) 524(0.677) |
| ***MEF2C*** | -5937.12 | -5937.12 | 0.00 | 1.00 | 0 | 0.02542/1.00000 | 1.00000/1.00000 | 367(0.665) 368(0.700) 371(0.671) 372(0.620) |
| ***MEPE*** | *-2666.22* | *-2697.69* | *62.92* | *0.00* | *1* | *0.12887/1.00000* | *4.20510/4.20510* | *192(0.939) 193(1.000) 194(0.793) 196(0.999) 197(1.000) 199(1.000)*  *200(0.951) 202(1.000) 203(0.956) 411(0.501)* |
| ***MGP*** | -3809.07 | -3809.07 | 0.00 | 1.00 | 0 | 0.08042/1.00000 | 1.00000/1.00000 |  |
| ***MITF*** | -7998.65 | -7998.61 | 0.00 | 1.00 | 0 | 0.00970/1.00000 | 1.00000/1.00000 |  |
| ***MMP2*** | -12828 | -12828 | 0.00 | 1.00 | 0 | 0.01811/1.00000 | 1.00000/1.00000 | 20(0.617) 22(0.675) 25(0.941) 169(0.868) 389(0.666) 391(0.957)  392(0.860) 639(0.727) 685(0.745) |
| ***MSX1*** | -4591.94 | -4591.94 | 0.00 | 1.00 | 0 | 0.00901/1.00000 | 1.00000/1.00000 | 133(0.688) 135(0.645) 146(0.909) 171(0.991) |
| ***NBR1*** | *-35659.9* | *-36156.8* | *993.82* | *0.00* | *1* | *0.08810/1.00000* | *9.86858/9.86858* | *120(0.730) 123(0.977) 124(1.000) 125(1.000) 126(1.000) 131(0.994)*  *132(1.000) 133(0.999) 134(1.000) 135(1.000) 137(1.000) 138(1.000)*  *139(1.000) 140(0.999) 151(1.000) 152(1.000) 153(1.000) 154(1.000)*  *155(1.000) 156(0.998) 157(1.000) 158(1.000) 297(1.000) 691(0.521)*  *692(0.636) 693(0.987) 694(1.000) 696(0.982) 697(0.935) 698(1.000)*  *699(1.000) 700(1.000) 701(0.999) 702(0.999) 986(0.516) 1139(0.999)* |
| ***NCDN*** | -26537.3 | -26537.3 | 0.00 | 1.00 | 0 | 0.08181/1.00000 | 1.00000/1.00000 | 39(0.758) 178(0.860) 244(0.943) 266(0.841) 377(0.928) 577(0.847)  678(0.848) 679(0.757) 700(0.843) 732(0.515) 739(0.978) |
| ***NF1*** | *-53438.9* | *-53487.3* | *96.86* | *0.00* | *1* | *0.00993/1.00000* | *9.09129/9.09129* | *1131(0.702) 1132(0.698) 1143(0.912) 1144(0.583) 1150(0.710)*  *1153(0.666) 1156(0.742) 1158(0.762) 2828(0.590) 2830(0.880)*  *2831(0.850) 2833(0.864) 2836(0.794) 2838(0.669) 2841(0.685)* |
| ***NOX4*** | -10981.3 | -10981.9 | 1.23 | 0.54 | 0 | 0.08283/1.00000 | 1.00000/1.00000 | 79(0.574) 285(0.693) 292(0.687) 541(0.630) 621(0.526) |
| ***OSR2*** | -4976.52 | -4976.52 | 0.00 | 1.00 | 0 | 0.02883/1.00000 | 1.00000/1.00000 |  |
| ***P2RX7*** | *-9704.65* | *-9772.76* | *136.23* | *0.00* | *1* | *0.09007/1.00000* | *5.14334/5.14334* | *41(0.954) 48(0.583) 67(0.957) 113(0.614) 131(0.569) 132(0.980)*  *305(0.883) 319(0.791) 348(0.736) 366(1.000) 367(0.940) 368(0.996)*  *370(0.997) 371(0.950) 372(0.999) 374(1.000) 375(0.997) 376(0.958)*  *382(0.997) 383(1.000) 392(0.792) 395(0.800) 396(0.678) 398(0.918)*  *399(0.578) 401(0.969) 402(0.932) 415(0.873) 428(0.657) 430(0.696)*  *431(0.914) 433(0.660) 434(0.702) 435(0.591) 474(0.755) 477(0.936)*  *480(0.959) 482(0.842) 483(0.996) 487(0.971) 491(0.546) 514(0.992)*  *634(0.644)* |
| ***PAPSS2*** | -17330.5 | -17330.5 | 0.00 | 1.00 | 0 | 0.05265/1.00000 | 1.00000/1.00000 |  |
| ***PKDCC*** | *-7100.92* | *-7133.46* | *65.08* | *0.00* | *1* | *0.03818/1.00000* | *5.87328/5.87328* | *394(0.791) 397(0.971) 399(0.904) 400(0.731) 401(1.000)* |
| ***PLA2G4A*** | -15401.2 | -15401.2 | 0.00 | 1.00 | 0 | 0.02689/1.00000 | 1.00000/1.00000 | 149(0.861) 152(0.887) 157(0.920) 173(0.729) 181(0.919) 187(0.922)  196(0.597) 459(0.795) 467(0.667) 468(0.918) 544(0.809) |
| ***PLXNB1*** | *-49618.2* | *-49667* | *97.59* | *0.00* | *1* | *0.03075/1.00000* | *5.92760/5.92760* | *707(0.640) 713(0.991) 715(0.952) 716(0.909) 717(0.926) 718(0.909)*  *719(0.665) 720(0.994) 721(0.963) 723(0.509) 724(0.768) 725(0.608)*  *739(1.000) 773(1.000) 792(0.611) 856(0.920) 879(1.000) 887(1.000)*  *888(0.988) 912(0.936) 913(0.711) 949(0.808)* |
| ***PTGER4*** | -10094.5 | -10094.5 | 0.00 | 1.00 | 0 | 0.03359/1.00000 | 1.00000/1.00000 | 461(0.888) |
| ***PTH*** | -3009.92 | -3009.92 | 0.00 | 1.00 | 0 | 0.08916/1.00000 | 1.00000/1.00000 |  |
| ***PTK2B*** | -15501.7 | -15501.7 | 0.00 | 1.00 | 0 | 0.04399/1.00000 | 1.00000/1.00000 | 132(0.546) 139(0.514) 140(0.919) 141(0.988) 142(0.984) 143(0.990)  144(0.980) 145(0.972) 146(0.942) 147(0.981) 149(0.913) 150(0.995)  151(0.979) 152(0.990) 153(0.986) 154(0.985) 155(0.826) 156(0.993)  157(0.959) 158(0.995) 159(0.989) 160(0.982) 161(0.996) 163(0.965)  164(0.985) 165(0.987) 166(0.974) 167(0.913) 168(0.990) 169(0.986)  170(0.988) 171(0.991) 172(0.997) 173(0.996) 174(0.983) 175(0.988)  176(0.947) 187(0.991) 189(0.658) 190(0.759) 191(0.989) 192(0.979)  194(0.964) 195(0.974) 196(0.990) 197(0.976) 198(0.984) 199(0.989)  200(0.984) 201(0.981) 202(0.964) 203(0.988) 204(0.941) 205(0.984)  206(0.971) 207(0.956) 208(0.979) 258(0.875) 279(0.700) 280(0.532)  285(0.939) 291(0.914) 392(0.990) 399(0.974) 404(0.946) 405(0.944)  406(0.950) 407(0.983) 413(0.822) 415(0.541) 433(0.722) 435(0.902)  443(0.635) 453(0.722) 455(0.506) 460(0.514) 465(0.951) 469(0.987)  470(0.996) 472(0.875) 473(0.973) 474(0.785) 476(0.769) 477(0.938)  480(0.689) 483(0.699) 490(0.831) 494(0.934) 495(0.984) 547(0.715)  627(0.750) 667(0.842) 817(0.575) 834(0.810) 844(0.690) 848(0.775)  888(0.648) 890(0.643) 893(0.633) 895(0.707) 898(0.673) 902(0.817)  904(0.936) 922(0.984) 934(0.964) 941(0.891) 942(0.952) 948(0.564)  949(0.844) 950(0.980) 951(0.982) 952(0.982) 956(0.741) 962(0.983)  995(0.869) 1069(0.697) 1073(0.543) |
| ***PTN*** | -2982.67 | -2982.8 | 0.26 | 0.88 | 0 | 0.02435/1.00000 | 1.00000/1.00000 |  |
| ***SBDS*** | -4464.97 | -4464.97 | 0.00 | 1.00 | 0 | 0.02893/1.00000 | 1.00000/1.00000 |  |
| ***SFRP1*** | -2174.38 | -2174.38 | 0.00 | 1.00 | 0 | 0.01370/1.00000 | 1.00000/1.00000 |  |
| ***SFRP2*** | -3930.95 | -3930.95 | 0.00 | 1.00 | 0 | 0.00875/1.00000 | 1.00000/1.00000 | 145(0.570) 171(0.666) |
| ***SH3PXD2B*** | -23738 | -23738 | 0.00 | 1.00 | 0 | 0.05515/1.00000 | 1.00000/1.00000 | 326(0.530) 332(0.904) 338(0.593) 339(0.645) 595(0.684) 814(0.513)  875(0.555) |
| ***SPP2*** | -7247.24 | -7247.24 | 0.00 | 1.00 | 0 | 0.13666/1.00000 | 1.00000/1.00000 |  |
| ***SRD5A1*** | *-4169.8* | *-4176.87* | *14.14* | *0.00* | *1* | *0.06051/1.00000* | *3.79435/3.79435* | *97(0.874) 132(0.603) 167(0.877) 257(0.986)* |
| ***SRGN*** | -3728.26 | -3728.26 | 0.00 | 1.00 | 0 | 0.09562/1.00000 | 1.00000/1.00000 | 128(0.853) |
| ***SULF1*** | *-16867.5* | *-16874.8* | *14.70* | *0.00* | *1* | *0.02090/1.00000* | *3.33994/3.33994* | *295(0.663) 296(0.727) 298(0.522) 299(0.750) 301(0.616) 303(0.989)*  *304(0.995) 313(0.539) 422(0.766) 610(0.675) 611(0.829) 614(0.956)*  *623(0.930)* |
| ***SULF2*** | -18844.2 | -18844.2 | 0.00 | 1.00 | 0 | 0.03326/1.00000 | 1.00000/1.00000 | 526(0.724) 647(0.610) |
| ***SYK*** | -8537.4 | -8538.31 | 1.80 | 0.41 | 0 | 0.03790/1.00000 | 3.58215/3.58215 |  |
| ***TCF7L2*** | *-5705.48* | *-5739.01* | *67.06* | *0.00* | *1* | *0.02609/1.00000* | *9.11472/9.11472* | *322(0.998) 323(0.991) 324(0.955) 325(0.647) 326(0.642) 327(0.942)*  *328(0.927) 329(0.987) 330(1.000) 683(0.835) 686(0.742) 689(1.000)*  *690(0.938) 691(0.747) 692(0.743) 693(0.599) 697(0.650)* |
| ***TFRC*** | *-30503.7* | *-30637.4* | *267.46* | *0.00* | *1* | *0.07512/1.00000* | *3.80699/3.80699* | *127(0.532) 144(0.999) 147(1.000) 151(0.989) 155(0.999) 156(1.000)*  *157(0.840) 169(0.976) 182(0.999) 183(0.956) 192(0.954) 195(1.000)*  *226(1.000) 233(0.628) 238(0.800) 246(0.604) 250(0.969) 260(0.975)*  *270(1.000) 310(1.000) 317(1.000) 364(1.000) 380(0.981) 382(0.996)*  *383(1.000) 397(0.994) 411(0.998) 557(1.000) 618(0.995) 627(0.999)*  *675(0.998) 677(0.975) 678(0.976) 771(0.886) 773(0.631)* |
| ***TGFB3*** | -5395.58 | -5395.54 | 0.00 | 1.00 | 0 | 0.01396/1.00000 | 1.00000/1.00000 |  |
| ***TNFAIP3*** | *-22869.8* | *-22900.1* | *60.69* | *0.00* | *1* | *0.06947/1.00000* | *3.64070/3.64070* | *296(0.534) 523(0.812) 532(1.000) 606(0.957) 613(0.909) 648(0.998)*  *674(0.687) 685(1.000) 686(0.912) 688(0.972) 691(0.999) 794(0.966)*  *798(0.895)* |
| ***TPH1*** | -8895.92 | -8895.92 | 0.00 | 1.00 | 0 | 0.05227/1.00000 | 1.00000/1.00000 | 144(0.754) 145(0.805) 146(0.504) 404(0.537) |
| ***TPP1*** | *-16173.4* | *-16374.8* | *402.76* | *0.00* | *1* | *0.04226/1.00000* | *43.722394/3.72239* | *147(0.631) 148(0.968) 149(0.995) 151(0.798) 153(0.945) 162(0.540)*  *165(0.816) 180(0.541) 186(0.938) 187(0.988) 188(0.982) 195(0.985)*  *201(0.948) 203(0.938) 204(0.773) 206(1.000) 207(0.997) 210(0.747)*  *211(0.914) 212(0.987) 214(1.000) 215(0.847) 218(0.868) 221(1.000)*  *226(0.553) 232(0.771) 239(0.935) 240(0.976) 246(0.993) 252(1.000)*  *253(0.976) 254(0.971) 255(1.000) 256(1.000) 257(1.000) 258(1.000)*  *259(0.938) 260(0.998) 261(1.000) 262(0.928) 263(0.828) 264(0.557)*  *267(1.000) 268(1.000) 269(1.000) 270(0.512) 271(0.997) 272(0.999)*  *274(0.961) 275(0.998) 276(1.000) 277(0.987) 280(0.995) 282(0.848)*  *283(0.990) 285(0.994) 286(1.000) 287(0.994) 288(1.000) 289(1.000)*  *290(0.869) 292(1.000) 296(0.660) 297(0.997) 298(0.977) 299(0.976)*  *302(0.997) 304(1.000) 313(1.000) 314(0.998) 315(0.925) 317(0.979)*  *318(0.986) 319(1.000) 320(0.995) 321(0.950) 322(1.000) 323(0.662)*  *324(0.959) 325(0.783) 327(1.000) 328(0.998) 329(0.888) 332(0.582)*  *333(0.984) 334(0.964) 335(0.999) 336(1.000) 337(0.827) 338(0.999)*  *341(0.993) 342(0.858) 343(0.691) 344(0.811) 345(0.712) 346(0.744)*  *349(0.700) 350(0.649) 351(0.512)* |
| ***TRAF6*** | -13864.9 | -13864.9 | 0.00 | 1.00 | 0 | 0.02859/1.00000 | 1.00000/1.00000 | 20(0.867) 21(0.909) 39(0.915) 44(0.659) 156(0.945) 200(0.515)  299(0.766) 312(0.742) 313(0.731) 513(0.627) 547(0.760) |
| ***TUFT1*** | -4508.75 | -4508.75 | 0.00 | 1.00 | 0 | 0.03711/1.00000 | 1.00000/1.00000 | 407(0.590) 408(0.604) |
| ***VEGFA*** | *-1952.84* | *-2032.37* | *159.06* | *0.00* | *1* | *0.04775/1.00000* | *999.00000/999.00000* | *241(0.949) 242(0.707) 243(0.987) 244(0.926) 245(1.000) 246(1.000)*  *247(1.000) 249(1.000) 250(0.997) 251(0.734) 252(0.957) 253(0.972)*  *254(0.993) 255(0.798) 256(0.991) 257(0.993) 258(0.747) 259(0.784)*  *260(0.990) 261(0.594) 262(0.978) 263(0.970) 264(0.962) 265(0.899)*  *266(0.871) 267(0.982) 268(0.801) 270(0.701) 273(0.960) 274(0.836)*  *275(0.992) 276(0.988)* |
