# Supplementary material for: Bone-associated gene evolution and the origin of flight in birds
Source: BMC Genomics. 2016 May 18;17:371. doi: 10.1186/s12864-016-2681-7 (PMC4870793; doi:10.1186/s12864-016-2681-7)
Supplement: Additional file 9: Table S6. — Branch-site model for mammals. Genes without flying mammals present in the alignment are marked (###). (DOC 138 kb) [file 12864_2016_2681_MOESM9_ESM.doc]

# Additional file 9: Table S6 - Branch-site model for mammals. Genes without flying mammals present in the alignment are marked (###).

| **Gene** | ***Lnl Alternate Model*** | ***Lnl Null Model*** | **LRT** | **p-value** | **q-value** | **Background (2a/2b)** | **Foreground (2a/2b)** | **Sites** |
| --- | --- | --- | --- | --- | --- | --- | --- | --- |
| ***ACVR2A*** | -9506.75 | -9506.75 | 0 | 1.00 | 0 | 0.02012/1.00000 | 1.00000/1.00000 | 422(0.523) |
| ***ACVR2B*** | *-9510.61* | *-9519.78* | *18.35* | *0.00* | *1* | *0.02013/1.00000* | *3.64849/3.64849* | *364(0.589) 366(0.980) 367(0.860) 368(0.900) 369(0.895) 370(0.977) 371(0.967) 373(0.987) 375(0.993) 376(0.986) 377(0.999) 378(0.908) 380(0.805) 389(0.840) 390(0.987) 391(0.986) 393(0.983)* |
| ***ADAM8*** | -38264 | -38264 | 0 | 1.00 | 0 | 0.14549/1.00000 | 1.00000/1.00000 |  |
| ***AHSG*** | *-23380.2* | *-23389.2* | *17.92* | *0.00* | *1* | *0.15743/1.00000* | *5.53856/5.53856* | *403(0.562) 422(0.867) 424(0.676) 426(0.971) 477(0.953) 479(0.724) 495(0.779) 496(0.854) 497(0.724) 505(0.994) 516(0.726) 519(0.827) 525(0.551) 528(0.542) 549(0.645) 562(0.522) 567(0.727) 574(0.766) 585(0.520) 591(0.937) 593(0.918) 594(0.839) 605(0.562)* |
| ***ANKH*** | -11803.8 | -11804.2 | 0.97 | 0.62 | 0 | 0.02250/1.00000 | 3.37177/3.37177 | 28(0.780) 30(0.692) 47(0.681) 239(0.940) 383(0.972) 384(0.897) |
| ***AQP1*** | -6463.87 | -6463.87 | 0 | 1.00 | 0 | 0.03580/1.00000 | 1.00000/1.00000 | 246(0.526) |
| ***ASPN*** | -10731.2 | -10731.3 | 0.09 | 0.96 | 0 | 0.07012/1.00000 | 1.57668/1.57668 | 365(0.966) |
| ***BCOR*** | *-54418.1* | *-54426.6* | *17.04* | *0.00* | *1* | *0.07962/1.00000* | *458.65148/458.65148* | *686(0.896) 689(0.914) 690(0.884) 691(0.940) 697(0.844) 698(0.877)* |
| ***BMP2*** | -10495.7 | -10495.7 | 0 | 1.00 | 0 | 0.06972/1.00000 | 1.00000/1.00000 |  |
| ***BMP7*** | -9437.09 | -9439.96 | 5.74 | 0.06 | 0 | 0.02402/1.00000 | 3.76986/3.76986 | 3(0.929) 10(0.946) 13(0.999) 14(0.952) 15(0.853) 24(0.958) 342(0.558) |
| ***BMPR1A*** | -10830.4 | -10830.4 | 0 | 1.00 | 0 | 0.02911/1.00000 | 1.00000/1.00000 |  |
| ***CA2*** | -10283.4 | -10283.4 | 0 | 1.00 | 0 | 0.11855/1.00000 | 1.00000/1.00000 | 228(0.906) 236(0.676) 255(0.643) |
| ***CARM1*** | ### | ### | ### | ### | 0 | ### | ### | ### |
| ***CBS*** | *-17981.1* | *-17989.5* | *16.89* | *0.00* | *1* | *0.06003/1.00000* | *999.00000/999.00000* | *7(0.599) 16(0.993) 17(0.989) 31(0.978) 36(0.515) 42(0.735) 43(0.845) 47(0.976) 229(0.765) 230(0.990) 544(0.523)* |
| ***CD38*** | -15893.2 | -15893.2 | 0 | 1.00 | 0 | 0.16063/1.00000 | 1.00000/1.00000 | 278(0.617) |
| ***CDX1*** | *-6520.91* | *-6529.42* | *17.02* | *0.00* | *1* | *0.08742/1.00000* | *3.33950/3.33950* | *60(0.546) 63(0.988) 64(0.990) 68(0.569) 71(0.997) 72(0.984) 73(0.988) 74(0.983) 77(0.575) 78(0.708) 79(0.971) 80(0.988) 82(0.991) 83(0.546) 84(0.623) 91(0.960) 96(0.841) 97(0.992) 98(0.919) 99(0.644) 100(0.981) 101(0.643) 102(0.974) 106(0.566) 108(0.658) 109(0.673) 111(0.994) 116(0.594) 117(0.850) 118(0.863) 119(0.996) 120(0.555) 122(0.618) 124(0.502) 125(0.979) 129(0.673) 130(0.746) 131(0.892) 132(0.777) 133(0.639) 134(0.985) 136(0.997) 137(0.989) 139(0.981) 140(0.983) 141(0.826) 143(0.993) 144(0.965) 145(0.550) 146(0.699) 147(0.635) 150(0.609) 151(0.964) 236(0.519) 256(0.536)* |
| ***CER1*** | -10679.9 | -10679.9 | 0 | 1.00 | 0 | 0.13131/1.00000 | 1.00000/1.00000 | 84(0.605) 86(0.811) |
| ***CITED2*** | *-3320.97* | *-3349.38* | *56.82* | *0.00* | *1* | *0.04912/1.00000* | *105.53600/105.53600* | *2(0.944) 3(0.997) 4(0.998) 5(0.805) 6(1.000) 7(0.999) 8(0.819) 10(0.736) 11(0.988) 12(0.999) 17(0.759) 18(0.998) 20(0.999) 21(0.998) 22(0.999) 23(0.998) 144(0.813) 175(0.759) 179(1.000) 191(0.607) 192(0.533)* |
| ***COL2A1*** | -33431 | -33431 | 0 | 1.00 | 0 | 0.04250/1.00000 | 1.00000/1.00000 |  |
| ***CREB3L1*** | -13027.6 | -13029.6 | 3.84 | 0.15 | 0 | 0.07561/1.00000 | 5.29594/5.29594 | 161(0.853) 173(0.954) 174(0.958) |
| ***CTHRC1*** | -6592.33 | --6592.33 | 0 | 1.0 | 0 | 0.02137/1.00000 | 1.00000/1.00000 |  |
| ***CTSK*** | -9144.52 | -9144.52 | 0 | 1.00 | 0 | 0.06755/1.00000 | 1.00000/1.00000 | 359(0.554) |
| ***DLX5*** | -5227.87 | -5227.87 | 0 | 1.00 | 0 | 0.05722/1.00000 | 1.00000/1.00000 |  |
| ***DUOX2*** | *-52460.3* | *-52468.7* | *16.78* | *0.00* | *1* | *0.07672/1.00000* | *9.36166/9.36166* | *209(0.935) 640(0.840) 787(0.562) 834(1.000) 868(0.973) 1046(0.971)* |
| ***DYM*** | -16297.8 | -16297.8 | 0 | 1.00 | 0 | 0.06204/1.00000 | 1.00000/1.00000 |  |
| ***EIF2AK3*** | -36504.8 | -36505.4 | 1.11 | 0.57 | 0 | 0.06088/1.00000 | 2.24029/2.24029 | 217(0.512) 302(0.540) 433(0.894) 500(0.562) 517(0.513) 519(0.975) 552(0.512) 565(0.523) 644(0.517) 651(0.561) 806(0.518) 888(0.601) 945(0.514) 956(0.508) 1072(0.977) |
| ***FBXL15*** | *-9253.63* | *-9267.64* | *28.03* | *0.00* | *1* | *0.05583/1.00000* | *999.00000/999.00000* | *96(0.785) 99(0.582) 101(0.653) 103(0.825) 161(0.726) 162(0.908) 163(0.998)* |
| ***FGF23*** | -9240.09 | -9243.14 | 6.1 | 0.05 | 0 | 0.09221/1.00000 | 1.94221/1.94221 | 25(0.938) 29(0.966) 60(0.755) 94(0.985) 129(0.685) 134(0.985) 137(0.937) 142(0.515) 143(0.963) 147(0.996) 149(0.989) 150(0.934) 151(0.958) 152(0.945) 153(0.775) 155(0.992) 163(0.595) 164(0.775) 166(0.900) 220(0.980) 225(0.973) 229(0.909) 250(0.992) 259(0.994) 267(0.682) 274(0.940) 277(0.759) 278(0.990) 279(0.967) |
| ***FGF8*** | -4471.59 | -4475.29 | 7.4 | 0.02 | 0 | 0.06874/1.00000 | 34.488073/4.48807 |  |
| ***GAS6*** | -26799.7 | -26799.7 | 0 | 1.00 | 0 | 0.09386/1.00000 | 1.00000/1.00000 | 656(0.843) |
| ***GHR*** | *-21376.4* | *-21390.7* | *28.5* | *0.00* | *1* | *0.10853/1.00000* | *6.79765/6.79765* | *42(0.988) 44(0.613) 46(0.647) 81(0.652) 98(0.604) 112(0.599) 248(0.995) 249(0.667) 253(0.959) 254(0.525) 260(0.992) 267(0.954) 268(0.989) 269(0.778) 271(0.953) 272(0.682) 273(0.801) 274(0.946) 275(0.622) 277(0.597) 278(0.802) 279(0.839) 283(0.506) 303(0.941) 420(0.571) 476(0.566) 485(0.802) 532(0.824)* |
| ***GPLD1*** | -32378.9 | -32378.9 | 0 | 1.00 | 0 | 0.11067/1.00000 | 1.00000/1.00000 |  |
| ***GPM6B*** | -7944.09 | -7947.39 | 6.59 | 0.04 | 0 | 0.04368/1.00000 | 4.43663/4.43663 | 126(0.555) 278(0.897) 281(0.882) 283(0.991) 285(0.993) 286(0.770) 287(0.881) 290(0.745) 292(0.995) 293(0.818) 294(0.996) |
| ***GREM1*** | -3767.5 | -3771 | 7.0 | 0.03 | 0 | 0.01275/1.00000 | 999.00000/999.00000 | 49(0.529) 50(0.992) |
| ***HOXA11*** | *-6640.21* | *-6645.28* | *10.15* | *0.01* | *1* | *0.03561/1.00000* | *13.909591/3.90959* | *104(0.917) 145(0.636) 152(0.606) 158(0.938) 159(0.544) 160(0.563) 165(0.995) 168(0.536) 192(0.615) 213(0.520) 214(0.780) 219(0.997) 237(0.551) 239(0.655) 241(0.728)* |
| ***HOXB4*** | -5244.76 | -5244.76 | 0 | 1.00 | 0 | 0.08706/1.00000 | 1.00000/1.00000 |  |
| ***HOXD11*** | -7329.58 | -7332.46 | 5.76 | 0.06 | 0 | 0.03976/1.00000 | 2.52941/2.52941 | 15(0.914) 16(0.924) 20(0.984) 23(0.976) 48(0.580) 135(0.910) 200(0.669) 222(0.913) 227(0.720) 229(0.544) 230(0.724) 235(0.924) 237(0.522) 238(0.887) 243(0.776) 251(0.950) 265(0.945) 298(0.985) |
| ***HSD17B2*** | -18634.6 | -18635.8 | 2.36 | 0.31 | 0 | 0.16656/1.00000 | 8.94531/8.94531 | 380(0.689) 387(0.752) |
| ***IAPP*** | -3344.99 | -3344.99 | 0 | 1.00 | 0 | 0.29829/1.00000 | 1.00000/1.00000 | 8(0.529) 78(0.530) 89(0.632) 90(0.510) 94(0.559) |
| ***IFITM5*** | ### | ### | ### | ### | 0 | ### | ### | ### |
| ***IGF1*** | -4649.72 | -4649.91 | 0.4 | 0.82 | 0 | 0.05968/1.00000 | 3.41979/3.41979 | 50(0.620) 56(0.805) 78(0.602) 115(0.526) 120(0.673) 162(0.855) 195(0.625) 223(0.523) |
| ***IHH*** | -10370.6 | -10370.6 | 0 | 1.00 | 0 | 0.04909/1.00000 | 1.00000/1.00000 |  |
| ***IL6*** | *-12718.7* | *-12736.7* | *36.0* | *0.00* | *1* | *0.31565/1.00000* | *1.00000/1.00000* |  |
| ***IL7*** | -3170.05 | -3170.05 | 0 | 1.00 | 0 | 0.15595/1.00000 | 1.00000/1.00000 |  |
| ***INPP5D*** | -39443.4 | -39444.3 | 1.82 | 0.40 | 0 | 0.08216/1.00000 | 2.64634/2.64634 | 5(0.862) 335(0.668) 774(0.538) 927(0.526) 990(0.870) |
| ***KLF10*** | -14264.5 | -14267 | 4.93 | 0.08 | 0 | 0.09000/1.00000 | 5.57220/5.57220 | 3(0.673) 5(0.988) |
| ***LRP6*** | -33466.3 | -33466.3 | 0 | 1.00 | 0 | 0.03107/1.00000 | 1.00000/1.00000 |  |
| ***LRRC17*** | -14431.9 | -14431.9 | 0 | 1.00 | 0 | 0.10008/1.00000 | 1.00000/1.00000 |  |
| ***MC4R*** | -8105.53 | -8105.53 | 0 | 1.00 | 0 | 0.03404/1.00000 | 1.00000/1.00000 | 21(0.633) 23(0.521) 39(0.954) 44(0.577) 48(0.980) 49(0.604) 106(0.703) 120(0.961) 123(0.654) 126(0.711) 127(0.566) 150(0.643) 178(0.868) 188(0.604) 197(0.504) 200(0.697) 274(0.576) 278(0.597) 281(0.638) 319(0.582) 329(0.591) 330(0.667) 331(0.584) 332(0.535) |
| ***MEF2A*** | -15241.1 | -15241.1 | 0 | 1.00 | 0 | 0.03979/1.00000 | 1.00000/1.00000 |  |
| ***MEF2C*** | -8227.45 | -8231.91 | 8.92 | 0.01 | 0 | 0.02326/1.00000 | 5.29382/5.29382 | 3(0.856) 4(0.533) 314(0.635) 317(0.593) 331(0.993) 332(0.834) 333(0.997) 334(0.888) 349(0.626) |
| ***MEPE/OC116*** | -27643.6 | -27643.6 | 0 | 1.00 | 0 | 0.30693/1.00000 | 1.00000/1.00000 |  |
| ***MGP*** | -3755.85 | -3757.05 | 2.38 | 0.30 | 0 | 0.08626/1.00000 | 4.89639/4.89639 | 122(0.978) |
| ***MITF*** | -11834.6 | -11837.3 | 5.44 | 0.07 | 0 | 0.04321/1.00000 | 502.09046/502.09046 | 379(0.841) |
| ***MMP2*** | -18877.6 | -18877.6 | 0 | 1.00 | 0 | 0.04956/1.00000 | 1.00000/1.00000 | 74(0.909) 96(0.925) 99(0.659) 261(0.577) 277(0.861) 354(0.957) 397(0.965) 398(0.928) 419(0.937) 426(0.893) 439(0.615) 501(0.947) 564(0.773) 614(0.530) 628(0.942) 649(0.511) 650(0.865) 678(0.922) |
| ***MSX1*** | -7270.76 | -7270.76 | 0 | 1.00 | 0 | 0.05439/1.00000 | 1.00000/1.00000 |  |
| ***NBR1*** | *-28589.6* | *-28604.1* | *29.02* | *0.00* | *1* | *0.08566/1.00000* | *124.63669/124.63669* | *614(0.966) 655(0.582) 824(0.533) 828(0.578) 896(0.636) 897(0.813) 898(0.770) 899(0.558) 900(0.987) 901(0.698) 931(0.504) 1118(0.808)* |
| ***NCDN*** | -15801.3 | -15801.8 | 1.0 | 0.60 | 0 | 0.04019/1.00000 | 6.39670/6.39670 |  |
| ***NF1*** | *-52203.1* | *-52211.1* | *16.0* | *0.00* | *1* | *0.03061/1.00000* | *6.51546/6.51546* | *4(0.612) 5(0.705) 6(0.562) 8(0.546) 10(0.568) 11(0.692) 15(0.682) 639(0.853) 953(0.956) 2090(0.949) 2392(0.977) 2394(0.946)* |
| ***NOX4*** | *-14871.4* | *-14892.2* | *41.5* | *0.00* | *1* | *0.06205/1.00000* | *137.40348/137.40348* | *65(0.993) 166(0.998) 167(0.999) 168(0.996) 169(0.989) 172(1.000) 173(0.700) 174(0.994) 176(0.994) 177(0.999) 178(0.554) 180(0.999) 182(0.680) 183(0.608) 187(0.565) 189(0.684) 191(0.945) 195(0.988) 646(0.722)* |
| ***OSR2*** | -5704.76 | -5704.76 | 0 | 1.00 | 0 | 0.01552/1.00000 | 1.00000/1.00000 |  |
| ***P2RX7*** | -18661.2 | -18661.2 | 0 | 1.00 | 0 | 0.08733/1.00000 | 1.00000/1.00000 |  |
| ***PAPSS2*** | -20069.6 | -20069.6 | 0 | 1.00 | 0 | 0.05372/1.00000 | 1.00000/1.00000 | 469(0.503) |
| ***PKDCC*** | *-9669.33* | *-9681.17* | *23.7* | *0.00* | *1* | *0.05209/1.00000* | *999.00000/999.00000* | *260(0.724) 412(0.839) 413(0.752) 414(0.988) 428(0.567) 429(0.998) 430(0.703) 435(0.867) 436(0.990) 437(0.880)* |
| ***PLA2G4A*** | -19445.8 | -19445.8 | 0 | 1.00 | 0 | 0.04338/1.00000 | 1.00000/1.00000 | 43(0.585) 116(0.533) 169(0.979) 185(0.505) 190(0.514) 298(0.523) 319(0.534) 395(0.503) 527(0.540) 665(0.513) |
| ***PLXNB1*** | -63680.3 | -63681.8 | 2.93 | 0.23 | 0 | 0.07287/1.00000 | 506.51457/506.51457 | 942(0.954) 1201(0.524) 1251(0.533) |
| ***PTGER4*** | -14524 | -14524 | 0.02 | 0.99 | 0 | 0.04784/1.00000 | 1.19031/1.19031 | 262(0.854) |
| ***PTH*** | -4253.08 | -4253.08 | 0 | 1.00 | 0 | 0.14664/1.00000 | 1.00000/1.00000 |  |
| ***PTK2B*** | -26164.9 | -26164.9 | 0 | 1.00 | 0 | 0.04765/1.00000 | 1.00000/1.00000 | 1060(0.955) |
| ***PTN*** | -5855.11 | -5859.67 | 9.12 | 0.01 | 0 | 0.03270/1.00000 | 346.92052/346.92052 | 59(0.559) 243(0.809) 247(0.992) 248(0.972) 256(0.954) |
| ***SBDS*** | -4284.04 | -4284.04 | 0 | 1.00 | 0 | 0.03142/1.00000 | 1.00000/1.00000 |  |
| ***SFRP1*** | -5877.33 | -5880.17 | 5.68 | 0.06 | 0 | 0.03053/1.00000 | 4.92320/4.92320 | 212(0.963) 214(0.950) 215(0.999) 216(0.846) |
| ***SFRP2*** | ### | ### | ### | ### | 0 | ### | ### | ### |
| ***SH3PXD2B*** | -25329 | -25329.1 | 0.10 | 0.95 | 0 | 0.06675/1.00000 | 2.00704/2.00704 | 598(0.534) 637(0.514) 667(0.551) 769(0.517) 818(0.579) 870(0.551) 901(0.934) 924(0.509) |
| ***SPP2*** | -10057 | -10057 | 0 | 1.00 | 0 | 0.20124/1.00000 | 1.00000/1.00000 |  |
| ***SRD5A1*** | -10679.4 | -10679.9 | 1.06 | 0.59 | 0 | 0.10498/1.00000 | 3.42141/3.42141 | 75(0.626) 76(0.513) 81(0.546) 144(0.567) 177(0.520) 191(0.543) |
| ***SRGN*** | -7774.55 | -7775.17 | 1.23 | 0.54 | 0 | 0.25597/1.00000 | 4.07129/4.07129 | 169(0.734) |
| ***SULF1*** | -27376 | -27376.3 | 0.58 | 0.75 | 0 | 0.04984/1.00000 | 6.39198/6.39198 |  |
| ***SULF2*** | -21440.5 | -21440.5 | 0 | 1.00 | 0 | 0.03403/1.00000 | 1.00000/1.00000 |  |
| ***SYK*** | -19147 | -19147 | 0 | 1.00 | 0 | 0.03487/1.00000 | 1.00000/1.00000 | 59(0.744) |
| ***TCF7L2*** | -14367.8 | -14367.8 | 0 | 1.00 | 0 | 0.05425/1.00000 | 1.00000/1.00000 |  |
| ***TFRC*** | -31558.6 | -31559.2 | 1.28 | 0.53 | 0 | 0.10969/1.00000 | 1.98032/1.98032 | 181(0.729) 293(0.775) 512(0.830) 515(0.800) 652(0.610) |
| ***TGFB3*** | -8997.91 | -8997.91 | 0 | 1.00 | 0 | 0.05127/1.00000 | 1.00000/1.00000 |  |
| ***TNFAIP3*** | -27045 | -27045 | 0 | 1.00 | 0 | 0.06405/1.00000 | 1.00000/1.00000 | 295(0.929) 518(0.893) |
| ***TPH1*** | -10673 | -10673 | 0 | 1.00 | 0 | 0.07311/1.00000 | 1.00000/1.00000 |  |
| ***TPP1*** | -15016.5 | -15018 | 2.90 | 0.23 | 0 | 0.07383/1.00000 | 13.676471/3.67647 |  |
| ***TRAF6*** | -16125.7 | -16127.3 | 3.36 | 0.19 | 0 | 0.07328/1.00000 | 6.92432/6.92432 | 174(0.771) 474(0.999) |
| ***TUFT1*** | -12505.2 | -12505.2 | 0.06 | 0.97 | 0 | 0.10948/1.00000 | 1.35057/1.35057 | 182(0.586) 306(0.528) 389(0.532) |
| ***VEGFA*** | *-9976.74* | *-9985.92* | *18.36* | *0.00* | *1* | *0.18652/1.00000* | *29.958952/9.95895* | *366(0.746) 407(0.999) 427(0.505)* |
