# Supplementary material for: Bone-associated gene evolution and the origin of flight in birds
Source: BMC Genomics. 2016 May 18;17:371. doi: 10.1186/s12864-016-2681-7 (PMC4870793; doi:10.1186/s12864-016-2681-7)
Supplement: Additional file 10: Table S7. — Results from the nested models (M0, M1a, M2a) likelihood ratio test results PAML from Avian dataset excluding flightless birds. The alignment length is on Amino acids (aa). Bold represents statistical significance (p < 0.05). Q-value estimations for multiple testing are represented as positive selected (1) and negative selected (0). (DOC 162 kb) [file 12864_2016_2681_MOESM10_ESM.doc]

# Additional file 10: Table S7 - Results from the nested models (M0, M1a, M2a) likelihood ratio test results PAML from Avian dataset excluding flightless birds. The alignment length is on Amino acids (aa). Bold represents statistical significance (p<0.05). Q-value estimations for multiple testing are represented as positive selected (1) and negative selected (0).

| Genes | Sequences  Number | Alignment Length (aa) | Model 0  (*lnL*) | Omega (ω) | Model 1 (*lnL*) | Model 2 (*lnL*) | 2∆L | p-value | q-value |
| --- | --- | --- | --- | --- | --- | --- | --- | --- | --- |
| *ACVR2A* | 38 | 513 | -10645.07 | 0.013 | -10507.79 | -10506.09 | 3.40 | 0.18 | 0 |
| ***ACVR2B*** | ***38*** | ***516*** | ***-7886.85*** | ***0.030*** | ***-7737.09*** | ***-7729.68*** | ***14.82*** | ***0.00*** | 1 |
| ***ADAM8*** | ***30*** | ***531*** | ***-18074.60*** | ***0.168*** | ***-17574.14*** | ***-17528.88*** | ***90.52*** | ***0.00*** | 1 |
| ***AHSG*** | ***38*** | ***384*** | ***-12807.46*** | ***0.518*** | ***-12224.69*** | ***-12109.60*** | ***230.17*** | ***0.00*** | 1 |
| ***ANKH*** | ***38*** | ***509*** | ***-7200.72*** | ***0.073*** | ***-6890.71*** | ***-6863.91*** | ***53.60*** | ***0.00*** | 1 |
| *AQP1* | 38 | 271 | -5025.04 | 0.059 | -4946.37 | -4946.37 | 0.00 | 1.00 | 0 |
| ***ASPN*** | ***34*** | ***641*** | ***-12598.08*** | ***0.161*** | ***-12167.47*** | ***-12106.63*** | ***121.67*** | ***0.00*** | 1 |
| ***BCOR*** | ***23*** | ***1798*** | ***-24734.76*** | ***0.133*** | ***-24306.66*** | ***-24302.37*** | ***8.58*** | ***0.01*** | 1 |
| ***BMP2*** | ***39*** | ***367*** | ***-3313.79*** | ***0.110*** | ***-3259.47*** | ***-3241.27*** | ***36.39*** | ***0.00*** | 1 |
| ***BMP7*** | ***38*** | ***425*** | ***-5790.48*** | ***0.030*** | ***-5744.58*** | ***-5744.58*** | ***0.00*** | ***0.00*** | 0 |
| ***BMPR1A*** | ***37*** | ***541*** | ***-8489.84*** | ***0.044*** | ***-8225.11*** | ***-8174.56*** | ***101.11*** | ***0.00*** | 1 |
| *CA2* | 39 | 271 | -5783.76 | 0.134 | -5621.07 | -5621.07 | 0.00 | 1.00 | 0 |
| *CARM1* | 15 | 412 | -5895.17 | 0.237 | -5791.35 | -5791.35 | 0.00 | 1.00 | 0 |
| *CBS* | 37 | 94 | -2499.90 | 0.286 | -2410.53 | -2407.69 | 5.67 | 0.06 | 0 |
| ***CD38*** | ***38*** | ***312*** | ***-7559.83*** | ***0.260*** | ***-7314.81*** | ***-7303.23*** | ***23.16*** | ***0.00*** | 1 |
| *CDX1* | 26 | 255 | -4110.95 | 0.064 | -4097.26 | -4097.26 | 0.00 | 1.00 | 0 |
| ***CER1*** | ***39*** | ***273*** | ***-8638.37*** | ***0.432*** | ***-8481.35*** | ***-8466.34*** | ***30.02*** | ***0.00*** | 1 |
| ***CITED2*** | ***14*** | ***293*** | ***-2200.92*** | ***0.140*** | ***-2160.52*** | ***-2151.21*** | ***18.60*** | ***0.00*** | 1 |
| *COL2A1* | 18 | 268 | -4306.20 | 0.048 | -4196.80 | -4196.06 | 1.48 | 0.48 | 0 |
| ***CREB3L1*** | ***38*** | ***520*** | ***-8970.77*** | ***0.199*** | ***-8589.54*** | ***-8516.43*** | ***146.22*** | ***0.00*** | 1 |
| *CTHRC1* | 39 | 244 | -4264.84 | 0.033 | -4175.09 | -4174.83 | 0.52 | 0.77 | 0 |
| *CTSK* | 17 | 132 | -2672.97 | 0.081 | -2520.12 | -2520.12 | 0.00 | 1.00 | 0 |
| ***DLX5*** | ***12*** | ***316*** | ***-4396.56*** | ***0.339*** | ***-3989.25*** | ***-3910.57*** | ***157.36*** | ***0.00*** | 1 |
| *DUOX2* | 37 | 1623 | -46751.43 | 0.156 | -45080.82 | -44963.51 | ***234.62*** | ***0.00*** | 1 |
| DYM | 18 | 680 | -8561.68 | 0.050 | -8511.40 | -8511.40 | 0.00 | 0.00 | 0 |
| *EIF2AK3* | 38 | 1012 | -21155.01 | 0.097 | -20848.30 | -20848.30 | 0.00 | 1.00 | 0 |
| ***FBXL15*** | ***38*** | ***297*** | ***-6062.12*** | ***0.077*** | ***-5942.52*** | ***-5929.56*** | ***25.93*** | ***0.00*** | 1 |
| ***FGF23*** | ***39*** | ***275*** | ***-6924.95*** | ***0.159*** | ***-6693.98*** | ***-6671.00*** | ***45.97*** | ***0.00*** | 1 |
| FGF8 | 31 | 229 | -3215.11 | 0.068 | -3150.91 | -3147.76 | 6.29 | 0.04 | 0 |
| ***GAS6*** | ***38*** | ***686*** | ***-14814.30*** | ***0.175*** | ***-14462.98*** | ***-14453.59*** | ***18.78*** | ***0.00*** | 1 |
| ***GHR*** | ***37*** | ***619*** | ***-13884.60*** | ***0.255*** | ***-13518.56*** | ***-13496.49*** | ***44.14*** | ***0.00*** | 1 |
| ***GPLD1*** | ***38*** | ***856*** | ***-23036.12*** | ***0.293*** | ***-22317.90*** | ***-22281.27*** | ***73.26*** | ***0.00*** | 1 |
| *GPM6B* | 39 | 328 | -3588.73 | 0.032 | -3550.38 | -3550.38 | 0.00 | 1.00 | 0 |
| *GREM1* | 38 | 186 | -3497.92 | 0.062 | -3401.58 | -3399.91 | 3.34 | 0.19 | 0 |
| ***HOXA11*** | ***26*** | ***310*** | ***-3553.72*** | ***0.252*** | ***-3380.23*** | ***-3362.06*** | ***36.35*** | ***0.00*** | 1 |
| ***HOXB4*** | ***35*** | ***56*** | ***-489.56*** | ***0.314*** | ***-464.63*** | ***-456.34*** | ***16.58*** | ***0.00*** | 1 |
| *HOXD11* | 39 | 284 | -3356.26 | 0.105 | -3325.57 | -3325.57 | 0.00 | 1.00 | 0 |
| ***HSD17B2*** | ***34*** | ***388*** | ***-8926.81*** | ***0.280*** | ***-8639.76*** | ***-8631.62*** | ***16.29*** | ***0.00*** | 1 |
| IAPP | 39 | 136 | -3409.81 | 0.292 | -3356.62 | -3353.02 | 7.19 | 0.03 | 0 |
| *IFITM5* | 12 | 151 | -2738.80 | 0.140 | -2680.62 | -2680.62 | 0.00 | 1.00 | 0 |
| ***IGF1*** | ***39*** | ***153*** | ***-1347.77*** | ***0.068*** | ***-1333.42*** | ***-1329.18*** | ***8.49*** | ***0.01*** | 1 |
| *IHH* | 19 | 312 | -5628.73 | 0.109 | -5513.09 | -5513.09 | 0.00 | 1.00 | 0 |
| ***IL6*** | ***38*** | ***169*** | ***-5156.56*** | ***0.339*** | ***-4962.24*** | ***-4954.85*** | ***14.77*** | ***0.00*** | 1 |
| *IL7* | 38 | 145 | -2853.93 | 0.322 | -2806.64 | -2806.64 | 0.00 | 1.00 | 0 |
| *INPP5D* | 18 | 1227 | -15643.65 | 0.139 | -15295.14 | -15292.65 | 4.99 | 0.08 | 0 |
| *KLF10* | 37 | 485 | -16008.66 | 0.237 | -15483.03 | -15483.03 | 0.00 | 1.00 | 0 |
| LRP6 | 39 | 1618 | -26989.33 | 0.025 | -26905.85 | -26905.85 | 0.00 | 0.00 | 0 |
| *LRRC17* | 39 | 442 | -8823.69 | 0.151 | -8673.44 | -8673.44 | 0.00 | 1.00 | 0 |
| *MC4R* | 39 | 331 | -5548.66 | 0.066 | -5439.00 | -5438.41 | 1.19 | 0.55 | 0 |
| *MEF2A* | 38 | 527 | -9871.77 | 0.093 | -9659.34 | -9657.33 | 4.03 | 0.13 | 0 |
| ***MEF2C*** | ***38*** | ***478*** | ***-5521.81*** | ***0.088*** | ***-5435.49*** | ***-5428.69*** | ***13.61*** | ***0.00*** | 1 |
| ***MEPE/OC116*** | ***33*** | ***82*** | ***-2822.23*** | ***0.403*** | ***-2679.89*** | ***-2646.83*** | ***66.11*** | ***0.00*** | 1 |
| ***MGP*** | ***38*** | ***104*** | ***-3439.48*** | ***0.338*** | ***-3336.14*** | ***-3331.85*** | ***8.58*** | ***0.01*** | 1 |
| *MITF* | 39 | 468 | -7137.95 | 0.029 | -7059.15 | -7059.04 | 0.22 | 0.90 | 0 |
| MMP2 | 37 | 672 | -11227.27 | 0.050 | -11012.07 | -11012.07 | 0.00 | 0.00 | 0 |
| *MSX1* | 26 | 294 | -3553.94 | 0.025 | -3496.73 | -3496.73 | 0.00 | 1.00 | 0 |
| ***NBR1*** | ***35*** | ***1081*** | ***-32486.96*** | ***0.351*** | ***-31039.73*** | ***-30549.15*** | ***981.16*** | ***0.00*** | 1 |
| ***NCDN*** | ***38*** | ***772*** | ***-24004.48*** | ***0.238*** | ***-23144.19*** | ***-23111.28*** | ***65.83*** | ***0.00*** | 1 |
| NF1 | 35 | 2834 | -48366.89 | 0.018 | -48098.43 | -48098.43 | 0.00 | 0.00 | 0 |
| ***NOX4*** | ***35*** | ***597*** | ***-9632.89*** | ***0.197*** | ***-9457.39*** | ***-9444.93*** | ***24.93*** | ***0.00*** | 1 |
| ***OSR2*** | ***23*** | ***316*** | ***-4457.98*** | ***0.089*** | ***-4300.34*** | ***-4293.68*** | ***13.31*** | ***0.00*** | 1 |
| ***P2RX7*** | ***8*** | ***598*** | ***-7642.22*** | ***0.738*** | ***-7458.05*** | ***-7392.41*** | ***131.27*** | ***0.00*** | 1 |
| *PAPSS2* | 37 | 626 | -15890.61 | 0.090 | -15610.47 | -15610.47 | 0.00 | 1.00 | 0 |
| ***PKDCC*** | ***34*** | ***296*** | ***-6475.57*** | ***0.184*** | ***-6121.68*** | ***-6072.20*** | ***98.97*** | ***0.00*** | 1 |
| PLA2G4A | 32 | 772 | -14215.26 | 0.061 | -14024.26 | -14024.26 | 0.00 | 0.00 | 0 |
| ***PLXNB1*** | ***36*** | ***2250*** | ***-42242.42*** | ***0.123*** | ***-40837.71*** | ***-40782.23*** | ***110.96*** | ***0.00*** | 1 |
| *PTGER4* | 37 | 478 | -8545.10 | 0.081 | -8185.77 | -8183.49 | 4.57 | 0.10 | 0 |
| *PTH* | 39 | 119 | -2638.89 | 0.182 | -2611.25 | -2611.25 | 0.00 | 1.00 | 0 |
| ***PTK2B*** | ***11*** | ***1127*** | ***-13278.19*** | ***0.114*** | ***-12857.83*** | ***-12846.63*** | ***22.40*** | ***0.00*** | 1 |
| *PTN* | 37 | 165 | -2565.05 | 0.085 | -2488.19 | -2488.14 | 0.09 | 0.96 | 0 |
| *SBDS* | 39 | 172 | -4076.74 | 0.043 | -4056.90 | -4056.90 | 0.00 | 1.00 | 0 |
| *SFRP1* | 5 | 314 | -1702.67 | 0.011 | -1702.67 | -1702.67 | 0.00 | 1.00 | 0 |
| *SFRP2* | 17 | 313 | -3371.82 | 0.075 | -3275.66 | -3275.66 | 0.00 | 1.00 | 0 |
| ***SH3PXD2B*** | ***35*** | ***927*** | ***-21070.61*** | ***0.160*** | ***-20403.38*** | ***-20397.61*** | ***11.54*** | ***0.00*** | 1 |
| *SPP2* | 38 | 193 | -6373.82 | 0.291 | -6225.41 | -6225.41 | 0.00 | 1.00 | 0 |
| ***SRD5A1*** | ***37*** | ***177*** | ***-3925.57*** | ***0.232*** | ***-3782.99*** | ***-3771.86*** | ***22.26*** | ***0.00*** | 1 |
| *SRGN* | 39 | 148 | -3117.31 | 0.211 | -3017.33 | -3014.43 | 5.81 | 0.05 | 0 |
| ***SULF1*** | ***34*** | ***892*** | ***-15263.03*** | ***0.084*** | ***-14681.72*** | ***-14655.89*** | ***51.67*** | ***0.00*** | 1 |
| *SULF2* | 39 | 906 | -17032.61 | 0.063 | -16799.55 | -16799.55 | 0.00 | 1.00 | 0 |
| SYK | 30 | 655 | -6438.86 | 0.071 | -6284.15 | -6280.63 | 7.05 | 0.03 | 0 |
| ***TCF7L2*** | ***39*** | ***511*** | ***-4619.15*** | ***0.116*** | ***-4490.53*** | ***-4459.37*** | ***62.30*** | ***0.00*** | 1 |
| ***TFRC*** | ***39*** | ***791*** | ***-27647.61*** | ***0.468*** | ***-26281.77*** | ***-26063.92*** | ***435.71*** | ***0.00*** | 1 |
| *TGFB3* | 39 | 296 | -4796.88 | 0.019 | -4766.66 | -4764.22 | 4.89 | 0.09 | 0 |
| ***TNFAIP3*** | ***39*** | ***833*** | ***-20438.86*** | ***0.180*** | ***-19784.69*** | ***-19741.28*** | ***86.83*** | ***0.00*** | 1 |
| TPH1 | 38 | 444 | -8032.17 | 0.094 | -7958.03 | -7958.18 | 0.00 | 0.00 | 0 |
| ***TPP1*** | ***18*** | ***480*** | ***-15758.18*** | ***0.414*** | ***-14224.01*** | ***-14029.19*** | ***389.64*** | ***0.00*** | 1 |
| *TRAF6* | 39 | 545 | -12724.70 | 0.068 | -12489.76 | -12489.70 | 0.12 | 0.94 | 0 |
| *TUFT1* | 38 | 394 | -3302.73 | 0.054 | -3286.35 | -3286.35 | 0.00 | 1.00 | 0 |
| ***VEGFA*** | ***39*** | ***239*** | ***-1816.00*** | ***0.212*** | ***-1781.23*** | ***-1755.33*** | ***51.79*** | ***0.00*** | 1 |
