# Supplementary material for: Bone-associated gene evolution and the origin of flight in birds
Source: BMC Genomics. 2016 May 18;17:371. doi: 10.1186/s12864-016-2681-7 (PMC4870793; doi:10.1186/s12864-016-2681-7)
Supplement: Additional file 11: Table S8. — Results from the nested models (M0, M1a, M2a) likelihood ratio test results PAML from Mammalian dataset excluding bats. The alignment length is on Amino acids (aa). Bold represents statistical significance (p < 0.05). Q-value estimations for multiple testing are represented as positive selected (1) and negative selected (0). (DOC 162 kb) [file 12864_2016_2681_MOESM11_ESM.doc]

# Additional file 11: Table S8 - Results from the nested models (M0, M1a, M2a) likelihood ratio test results PAML from Mammalian dataset excluding bats. The alignment length is on Amino acids (aa). Bold represents statistical significance (p<0.05). Q-value estimations for multiple testing are represented as positive selected (1) and negative selected (0).

| Genes | Sequences  Number | Alignment Length (aa) | Model 0  (*lnL*) | Omega (ω) | Model 1 (*lnL*) | Model 2 (*lnL*) | 2∆L | p-value | q-value |
| --- | --- | --- | --- | --- | --- | --- | --- | --- | --- |
| *ACVR2A* | 35 | 581 | -9406.48 | 0.051 | -9202.95 | -9202.89 | 0.13 | 0.94 | 0 |
| *ACVR2B* | 30 | 493 | -8754.43 | 0.021 | -8753.81 | -8753.81 | 0.00 | 1.00 | 0 |
| ***ADAM8*** | ***27*** | ***941*** | ***-36178.67*** | ***0.270*** | ***-35313.46*** | ***-35302.65*** | ***21.61*** | ***0.00*** | ***1*** |
| ***AHSG*** | ***35*** | ***662*** | ***-22343.38*** | ***0.473*** | ***-21707.20*** | ***-21654.40*** | ***105.59*** | ***0.00*** | ***1*** |
| *ANKH* | 34 | 492 | -11199.84 | 0.029 | -11172.51 | -11172.51 | 0.00 | 1.00 | 0 |
| ***AQP1*** | ***26*** | ***304*** | ***-6197.30*** | ***0.055*** | ***-6060.45*** | ***-6054.81*** | ***11.27*** | ***0.00*** | ***1*** |
| *ASPN* | 35 | 390 | -10426.24 | 0.107 | -10311.74 | -10311.74 | 0.00 | 1.00 | 0 |
| *BCOR* | 35 | 1859 | -51738.97 | 0.118 | -51167.67 | -51167.67 | 0.00 | 1.00 | 0 |
| *BMP2* | 34 | 400 | -10201.07 | 0.103 | -10082.75 | -10082.75 | 0.00 | 1.00 | 0 |
| *BMP7* | 33 | 438 | -8948.24 | 0.026 | -8935.29 | -8935.29 | 0.00 | 1.00 | 0 |
| *BMPR1A* | 28 | 544 | -10633.37 | 0.042 | -10584.02 | -10584.02 | 0.00 | 1.00 | 0 |
| ***CA2*** | ***34*** | ***274*** | ***-9592.49*** | ***0.266*** | ***-9288.79*** | ***-9260.89*** | ***55.80*** | ***0.00*** | ***1*** |
| *CARM1* | 13 | 658 | -7523.57 | 0.089 | -7421.46 | -7421.46 | 0.00 | 1.00 | 0 |
| *CBS* | 28 | 609 | -17014.11 | 0.098 | -16661.18 | -16661.16 | 0.05 | 0.98 | 0 |
| ***CD38*** | ***35*** | ***321*** | ***-15150.34*** | ***0.527*** | ***-14745.45*** | ***-14724.34*** | ***42.23*** | ***0.00*** | ***1*** |
| *CDX1* | 22 | 296 | -5490.87 | 0.136 | -5437.12 | -5437.12 | 0.00 | 1.00 | 0 |
| *CER1* | 31 | 293 | -10300.35 | 0.337 | -10024.30 | -10024.30 | 0.00 | 1.00 | 0 |
| *CITED2* | 20 | 276 | -3041.40 | 0.066 | -3023.71 | -3023.71 | 0.00 | 1.00 | 0 |
| ***COL2A1*** | ***32*** | ***1521*** | ***-32665.34*** | ***0.117*** | ***-31910.27*** | ***-31752.44*** | ***315.65*** | ***0.00*** | ***1*** |
| *CREB3L1* | 34 | 547 | -12425.08 | 0.080 | -12380.69 | -12379.19 | 3.02 | 0.22 | 0 |
| *CTHRC1* | 34 | 256 | -6573.79 | 0.097 | -6308.99 | -6308.99 | 0.00 | 1.00 | 0 |
| *CTSK* | 35 | 353 | -8886.67 | 0.126 | -8673.63 | -8673.63 | 0.00 | 1.00 | 0 |
| *DLX5* | 33 | 299 | -5009.57 | 0.064 | -4998.50 | -4998.50 | 0.00 | 1.00 | 0 |
| ***DUOX2*** | ***34*** | ***1634*** | ***-51245.05*** | ***0.156*** | ***-49632.39*** | ***-49601.87*** | ***61.05*** | ***0.00*** | ***1*** |
| *DYM* | 34 | 686 | -15587.57 | 0.079 | -15472.43 | -15472.43 | 0.00 | 1.00 | 0 |
| *EIF2AK3* | 37 | 1150 | -35416.83 | 0.129 | -34564.84 | -34564.84 | 0.00 | 1.00 | 0 |
| ***FBXL15*** | ***28*** | ***393*** | ***-9233.78*** | ***0.130*** | ***-8956.86*** | ***-8928.31*** | ***57.09*** | ***0.00*** | ***1*** |
| *FGF23* | 30 | 263 | -8453.28 | 0.123 | -8312.52 | -8312.52 | 0.00 | 1.00 | 0 |
| *FGF8* | 25 | 211 | -4393.42 | 0.093 | -4334.63 | -4334.63 | 0.00 | 1.00 | 0 |
| *GAS6* | 30 | 791 | -26013.48 | 0.154 | -25384.71 | -25384.71 | 0.00 | 1.00 | 0 |
| *GHR* | 35 | 694 | -20685.30 | 0.298 | -20150.01 | -20150.01 | 0.00 | 1.00 | 0 |
| *GPLD1* | 35 | 881 | -32273.19 | 0.231 | -31507.88 | -31507.88 | 0.00 | 1.00 | 0 |
| ***GPM6B*** | ***33*** | ***378*** | ***-7761.47*** | ***0.104*** | ***-7453.51*** | ***-7446.87*** | ***13.28*** | ***0.00*** | ***1*** |
| *GREM1* | 30 | 240 | -3536.47 | 0.021 | -3511.95 | -3511.67 | 0.56 | 0.75 | 0 |
| *HOXA11* | 31 | 346 | -6090.40 | 0.141 | -5851.54 | -5851.54 | 0.00 | 1.00 | 0 |
| *HOXB4* | 25 | 294 | -4724.34 | 0.121 | -4654.95 | -4654.95 | 0.00 | 1.00 | 0 |
| *HOXD11* | 16 | 367 | -6241.17 | 0.166 | -6119.74 | -6119.74 | 0.00 | 1.00 | 0 |
| ***HSD17B2*** | ***33*** | ***403*** | ***-17929.66*** | ***0.370*** | ***-17414.53*** | ***-17306.08*** | ***216.90*** | ***0.00*** | ***1*** |
| *IAPP* | 27 | 98 | -3152.16 | 0.381 | -3140.84 | -3140.84 | 0.00 | 1.00 | 0 |
| *IFITM5* | 24 | 135 | -3392.62 | 0.102 | -3378.30 | -3378.30 | 0.00 | 1.00 | 0 |
| ***IGF1*** | ***26*** | ***241*** | ***-4495.67*** | ***0.193*** | ***-4298.48*** | ***-4281.81*** | ***33.34*** | ***0.00*** | ***1*** |
| *IHH* | 31 | 461 | -9882.09 | 0.068 | -9751.33 | -9751.33 | 0.00 | 1.00 | 0 |
| ***IL6*** | ***33*** | ***317*** | ***-11924.37*** | ***0.701*** | ***-11800.30*** | ***-11789.39*** | ***21.83*** | ***0.00*** | ***1*** |
| ***IL7*** | ***13*** | ***185*** | ***-2981.27*** | ***0.607*** | ***-2919.37*** | ***-2906.72*** | ***25.31*** | ***0.00*** | ***1*** |
| ***INPP5D*** | ***31*** | ***1262*** | ***-37720.71*** | ***0.133*** | ***-36775.65*** | ***-36753.92*** | ***43.46*** | ***0.00*** | ***1*** |
| *KLF10* | 30 | 512 | -13507.43 | 0.150 | -13292.20 | -13290.68 | 3.03 | 0.22 | 0 |
| *LRP6* | 36 | 1639 | -32626.01 | 0.042 | -32383.93 | -32383.93 | 0.00 | 1.00 | 0 |
| *LRRC17* | 35 | 458 | -13924.29 | 0.141 | -13755.90 | -13753.39 | 5.02 | 0.08 | 0 |
| *MC4R* | 30 | 341 | -7536.21 | 0.048 | -7445.67 | -7445.67 | 0.00 | 1.00 | 0 |
| ***MEF2A*** | ***36*** | ***574*** | ***-14341.12*** | ***0.135*** | ***-13649.53*** | ***-13567.73*** | ***163.60*** | ***0.00*** | ***1*** |
| ***MEF2C*** | ***32*** | ***494*** | ***-7988.24*** | ***0.138*** | ***-7472.33*** | ***-7204.98*** | ***534.70*** | ***0.00*** | ***1*** |
| *MEPE/OC116* | 33 | 637 | -26465.69 | 0.503 | -26182.41 | -26182.41 | 0.00 | 1.00 | 0 |
| *MGP* | 33 | 129 | -3632.99 | 0.196 | -3548.87 | -3548.87 | 0.00 | 1.00 | 0 |
| *MITF* | 34 | 533 | -11031.26 | 0.080 | -10883.46 | -10883.46 | 0.00 | 1.00 | 0 |
| *MMP2* | 34 | 687 | -17693.44 | 0.069 | -17436.69 | -17436.69 | 0.00 | 1.00 | 0 |
| *MSX1* | 30 | 320 | -6660.59 | 0.071 | -6609.90 | -6609.90 | 0.00 | 1.00 | 0 |
| ***NBR1*** | ***30*** | ***1118*** | ***-25849.32*** | ***0.260*** | ***-25122.29*** | ***-25077.78*** | ***89.02*** | ***0.00*** | ***1*** |
| *NCDN* | 26 | 764 | -15109.36 | 0.049 | -15042.14 | -15042.14 | 0.00 | 1.00 | 0 |
| *NF1* | 34 | 2854 | -50058.78 | 0.036 | -49924.97 | -49924.97 | 0.00 | 1.00 | 0 |
| *NOX4* | 33 | 661 | -14346.30 | 0.165 | -13994.92 | -13994.92 | 0.00 | 1.00 | 0 |
| *OSR2* | 30 | 312 | -5452.18 | 0.071 | -5203.29 | -5201.96 | 2.67 | 0.26 | 0 |
| *P2RX7* | 33 | 613 | -18694.44 | 0.206 | -18115.33 | -18115.33 | 0.00 | 1.00 | 0 |
| ***PAPSS2*** | ***37*** | ***653*** | ***-19747.86*** | ***0.098*** | ***-19174.31*** | ***-19139.23*** | ***70.16*** | ***0.00*** | ***1*** |
| *PKDCC* | 32 | 504 | -9250.45 | 0.082 | -9096.71 | -9096.71 | 0.00 | 1.00 | 0 |
| *PLA2G4A* | 36 | 764 | -18777.67 | 0.068 | -18540.50 | -18540.50 | 0.00 | 1.00 | 0 |
| *PLXNB1* | 33 | 2247 | -63278.28 | 0.142 | -61794.93 | -61794.93 | 0.00 | 1.00 | 0 |
| ***PTGER4*** | ***31*** | ***546*** | ***-13486.92*** | ***0.097*** | ***-13116.88*** | ***-13105.46*** | ***22.84*** | ***0.00*** | ***1*** |
| *PTH* | 33 | 126 | -4261.61 | 0.317 | -4166.15 | -4166.15 | 0.00 | 1.00 | 0 |
| *PTK2B* | 35 | 1036 | -25059.53 | 0.056 | -24874.18 | -24874.18 | 0.00 | 1.00 | 0 |
| ***PTN*** | ***33*** | ***257*** | ***-5644.27*** | ***0.173*** | ***-5379.21*** | ***-5344.97*** | ***68.48*** | ***0.00*** | ***1*** |
| *SBDS* | 20 | 255 | -4190.49 | 0.043 | -4156.43 | -4156.43 | 0.00 | 1.00 | 0 |
| *SFRP1* | 29 | 329 | -5655.21 | 0.045 | -5587.14 | -5587.14 | 0.00 | 1.00 | 0 |
| *SFRP2* | 18 | 304 | -4128.58 | 0.037 | -4070.90 | -4070.90 | 0.00 | 1.00 | 0 |
| *SH3PXD2B* | 28 | 939 | -24992.00 | 0.126 | -24375.06 | -24375.06 | 0.00 | 1.00 | 0 |
| *SPP2* | 31 | 224 | -9585.94 | 0.355 | -9438.75 | -9438.75 | 0.00 | 1.00 | 0 |
| *SRD5A1* | 31 | 266 | -10233.89 | 0.283 | -9882.94 | -9882.94 | 0.00 | 1.00 | 0 |
| *SRGN* | 31 | 212 | -7304.86 | 0.408 | -7208.28 | -7208.28 | 0.00 | 1.00 | 0 |
| *SULF1* | 35 | 1169 | -26723.49 | 0.094 | -26233.24 | -26231.02 | 4.45 | 0.11 | 0 |
| *SULF2* | 29 | 929 | -20950.11 | 0.051 | -20593.56 | -20593.56 | 0.00 | 1.00 | 0 |
| *SYK* | 34 | 665 | -18425.08 | 0.071 | -17946.38 | -17946.38 | 0.00 | 1.00 | 0 |
| *TCF7L2* | 35 | 697 | -13135.96 | 0.137 | -12745.36 | -12745.36 | 0.00 | 1.00 | 0 |
| ***TFRC*** | ***35*** | ***807*** | ***-30582.97*** | ***0.322*** | ***-29381.32*** | ***-29308.63*** | ***145.39*** | ***0.00*** | ***1*** |
| *TGFB3* | 33 | 490 | -8290.63 | 0.055 | -8247.88 | -8247.88 | 0.00 | 1.00 | 0 |
| *TNFAIP3* | 36 | 809 | -26341.05 | 0.109 | -25820.67 | -25820.67 | 0.00 | 1.00 | 0 |
| ***TPH1*** | ***28*** | ***535*** | ***-10428.58*** | ***0.125*** | ***-10184.70*** | ***-10138.97*** | ***91.45*** | ***0.00*** | ***1*** |
| *TPP1* | 31 | 589 | -14602.98 | 0.197 | -14271.47 | -14271.47 | 0.00 | 1.00 | 0 |
| *TRAF6* | 34 | 559 | -15631.06 | 0.127 | -15328.36 | -15328.36 | 0.00 | 1.00 | 0 |
| *TUFT1* | 33 | 422 | -12104.74 | 0.212 | -11819.73 | -11819.73 | 0.00 | 1.00 | 0 |
| *VEGFA* | 30 | 484 | -9537.62 | 0.345 | -9415.48 | -9415.48 | 0.00 | 1.00 | 0 |
