# Supplementary material for: Bone-associated gene evolution and the origin of flight in birds
Source: BMC Genomics. 2016 May 18;17:371. doi: 10.1186/s12864-016-2681-7 (PMC4870793; doi:10.1186/s12864-016-2681-7)
Supplement: Additional file 12: Table S9. — Branch model for birds. Bold represents statistical significance (p < 0.05). Q-value estimations for multiple testing are represented as positive selected (1) and negative selected (0). (DOC 114 kb) [file 12864_2016_2681_MOESM12_ESM.doc]

# Additional file 12: Table S9 - Branch model for birds. Bold represents statistical significance (p<0.05). Q-value estimations for multiple testing are represented as positive selected (1) and negative selected (0).

| Gene | Model One-Ratio | Model Two-Ratio | Background Branch | Foreground Branch | LRT | p-value | q-value |
| --- | --- | --- | --- | --- | --- | --- | --- |
| ***ACVR2A*** | ***-11702.76*** | ***-11690.93*** | ***0.0138*** | ***0.0539*** | ***23.66*** | ***0.000*** | ***1*** |
| ***ACVR2B*** | ***-8605.05*** | ***-8602.24*** | ***0.0300*** | ***0.0114*** | ***5.62*** | ***0.018*** | ***1*** |
| *ADAM8* | -18973.37 | -18973.30 | 0.1696 | 0.1807 | 0.13 | 0.724 | 0 |
| *AHSG* | -14302.16 | -14300.20 | 0.5127 | 0.6867 | 3.92 | 0.048 | 0 |
| ***ANKH*** | ***-8167.20*** | ***-8163.34*** | ***0.0741*** | ***0.0344*** | ***7.72*** | ***0.005*** | ***1*** |
| *AQP1* | -5813.30 | -5812.02 | 0.0589 | 0.0859 | 2.56 | 0.109 | 0 |
| *ASPN* | -14968.79 | -14968.67 | 0.1618 | 0.1713 | 0.24 | 0.621 | 0 |
| *BCOR* | -32581.66 | -32580.31 | 0.1273 | 0.1420 | 2.70 | 0.100 | 0 |
| *BMP2* | -3609.81 | -3609.47 | 0.1132 | 0.0906 | 0.68 | 0.411 | 0 |
| *BMP7* | -6531.04 | -6529.23 | 0.0304 | 0.0159 | 3.63 | 0.057 | 0 |
| *BMPR1A* | -9405.23 | -9405.10 | 0.0441 | 0.0372 | 0.26 | 0.609 | 0 |
| *CA2* | -6611.39 | -6611.26 | 0.1340 | 0.1208 | 0.27 | 0.602 | 0 |
| *CARM1* | -6668.17 | -6666.78 | 0.2381 | 0.1713 | 2.78 | 0.096 | 0 |
| *CBS* | -2649.25 | -2775.36 | 0.2777 | 0.3444 | 0.00 | 1.000 | 0 |
| ***CD38*** | ***-8767.54*** | ***-8764.59*** | ***0.2612*** | ***0.3862*** | ***5.90*** | ***0.015*** | ***1*** |
| *CDX1* | -4528.40 | -4528.09 | 0.0627 | 0.0494 | 0.61 | 0.436 | 0 |
| *CER1* | -9925.34 | -9924.79 | 0.4320 | 0.3718 | 1.11 | 0.292 | 0 |
| *CITED2* | -2556.44 | -2556.39 | 0.1409 | 0.1276 | 0.11 | 0.745 | 0 |
| ***COL2A1*** | ***-5208.96*** | ***-5204.13*** | ***0.0471*** | ***0.0089*** | ***9.67*** | ***0.002*** | ***1*** |
| *CREB3L1* | -10058.35 | -10058.29 | 0.1971 | 0.2092 | 0.12 | 0.733 | 0 |
| *CTHRC1* | -5164.86 | -5163.06 | 0.0399 | 0.0704 | 3.59 | 0.058 | 0 |
| ***CTSK*** | ***-3109.43*** | ***-3106.93*** | ***0.0864*** | ***0.0189*** | ***5.00*** | ***0.025*** | ***1*** |
| ***DLX5*** | ***-5587.19*** | ***-5583.43*** | ***0.3343*** | ***0.2134*** | ***7.53*** | ***0.006*** | ***1*** |
| *DUOX2* | -53940.85 | -53939.64 | 0.1566 | 0.1729 | 2.42 | 0.120 | 0 |
| *DYM* | -12203.96 | -12201.72 | 0.0486 | 0.0307 | 4.48 | 0.034 | 0 |
| *EIF2AK3* | -23818.04 | -23817.76 | 0.0978 | 0.0896 | 0.56 | 0.456 | 0 |
| *FBXL15* | -7031.28 | -7030.55 | 0.0761 | 0.0978 | 1.48 | 0.224 | 0 |
| *FGF23* | -7909.50 | -7908.63 | 0.1602 | 0.2003 | 1.74 | 0.187 | 0 |
| *FGF8* | -3605.86 | -3604.32 | 0.0658 | 0.0389 | 3.07 | 0.080 | 0 |
| *GAS6* | -16805.46 | -16805.41 | 0.1758 | 0.1691 | 0.09 | 0.769 | 0 |
| *GHR* | -15525.26 | -15524.21 | 0.2534 | 0.2048 | 2.11 | 0.146 | 0 |
| *GPLD1* | -26406.69 | -26406.33 | 0.2923 | 0.3148 | 0.70 | 0.402 | 0 |
| *GPM6B* | -4105.11 | -4105.11 | 0.0314 | 0.0312 | 0.00 | 0.985 | 0 |
| *GREM1* | -3942.12 | -3941.87 | 0.0622 | 0.0493 | 0.50 | 0.481 | 0 |
| *HOXA11* | -3932.45 | -3930.47 | 0.2544 | 0.1452 | 3.96 | 0.047 | 0 |
| *HOXB4* | -657.23 | -656.50 | 0.2870 | 0.1413 | 1.46 | 0.227 | 0 |
| ***HOXD11*** | ***-3775.63*** | ***-3771.56*** | ***0.1028*** | ***0.2277*** | ***8.14*** | ***0.004*** | ***1*** |
| *HSD17B2* | -10641.72 | -10639.81 | 0.2839 | 0.2171 | 3.83 | 0.050 | 0 |
| *IAPP* | -3801.58 | -3800.18 | 0.2923 | 0.4559 | 2.81 | 0.094 | 0 |
| *IFITM5* | -3307.72 | -3306.17 | 0.1379 | 0.0895 | 3.11 | 0.078 | 0 |
| *IGF1* | -1467.27 | -1467.26 | 0.0657 | 0.0723 | 0.02 | 0.888 | 0 |
| *IHH* | -6585.46 | -6585.30 | 0.1053 | 0.1188 | 0.31 | 0.575 | 0 |
| *IL6* | -5727.51 | -5726.01 | 0.3211 | 0.1841 | 3.01 | 0.083 | 0 |
| *IL7* | -3418.75 | -3417.13 | 0.3272 | 0.5000 | 3.23 | 0.072 | 0 |
| ***INPP5D*** | ***-18906.06*** | ***-18903.35*** | ***0.1393*** | ***0.1091*** | ***5.42*** | ***0.020*** | ***1*** |
| *KLF10* | -18012.24 | -18011.57 | 0.2383 | 0.2056 | 1.32 | 0.250 | 0 |
| *LRP6* | -30462.53 | -30462.47 | 0.0253 | 0.0240 | 0.11 | 0.742 | 0 |
| *LRRC17* | -10115.64 | -10115.14 | 0.1516 | 0.1770 | 1.01 | 0.316 | 0 |
| ***MC4R*** | ***-6203.29*** | ***-6200.26*** | ***0.0664*** | ***0.0313*** | ***6.06*** | ***0.014*** | ***1*** |
| ***MEF2A*** | ***-11043.54*** | ***-11037.65*** | ***0.0932*** | ***0.0475*** | ***11.78*** | ***0.001*** | ***1*** |
| *MEF2C* | -6133.89 | -6133.23 | 0.0893 | 0.1216 | 1.32 | 0.250 | 0 |
| *MEPE* | -2877.16 | -2876.95 | 0.4067 | 0.2463 | 0.42 | 0.515 | 0 |
| *MGP* | -3947.00 | -3946.10 | 0.3378 | 0.4584 | 1.79 | 0.180 | 0 |
| *MITF* | -8087.20 | -8086.98 | 0.0292 | 0.0240 | 0.44 | 0.509 | 0 |
| *MMP2* | -13067.17 | -13066.76 | 0.0500 | 0.0429 | 0.82 | 0.365 | 0 |
| *MSX1* | -4678.14 | -4677.98 | 0.0256 | 0.0355 | 0.32 | 0.574 | 0 |
| *NBR1* | -37167.42 | -37167.29 | 0.3541 | 0.3682 | 0.26 | 0.609 | 0 |
| *NCDN* | -27557.63 | -27557.59 | 0.2400 | 0.2462 | 0.08 | 0.771 | 0 |
| *NF1* | -53801.41 | -53800.99 | 0.0182 | 0.0158 | 0.85 | 0.357 | 0 |
| *NOX4* | -11228.10 | -11227.84 | 0.1964 | 0.2184 | 0.51 | 0.475 | 0 |
| *OSR2* | -5164.50 | -5164.10 | 0.0870 | 0.1076 | 0.79 | 0.373 | 0 |
| ***P2RX7*** | ***-9939.44*** | ***-9935.59*** | ***0.7816*** | ***0.5415*** | ***7.70*** | ***0.006*** | ***1*** |
| *PAPSS2* | -17640.91 | -17640.87 | 0.0921 | 0.0957 | 0.07 | 0.789 | 0 |
| *PKDCC* | -7580.94 | -7579.52 | 0.1814 | 0.2385 | 2.86 | 0.091 | 0 |
| *PLA2G4A* | -15650.53 | -15650.01 | 0.0616 | 0.0512 | 1.04 | 0.309 | 0 |
| ***PLXNB1*** | ***-49382.17*** | ***-49379.55*** | ***0.1223*** | ***0.1432*** | ***5.24*** | ***0.022*** | ***1*** |
| ***PTGER4*** | ***-10433.82*** | ***-10417.49*** | ***0.0772*** | ***0.1860*** | ***32.65*** | ***0.000*** | ***1*** |
| *PTH* | -3004.34 | -3004.34 | 0.1816 | 0.1844 | 0.00 | 0.954 | 0 |
| ***PTK2B*** | ***-16055.71*** | ***-16045.31*** | ***0.1129*** | ***0.0641*** | ***20.81*** | ***0.000*** | ***1*** |
| ***PTN*** | ***-3060.44*** | ***-3053.29*** | ***0.0820*** | ***0.2302*** | ***14.29*** | ***0.000*** | ***1*** |
| *SBDS* | -4495.00 | -4494.70 | 0.0438 | 0.0323 | 0.59 | 0.443 | 0 |
| *SFRP1* | -2190.51 | -2190.42 | 0.0177 | 0.0224 | 0.19 | 0.659 | 0 |
| *SFRP2* | -4193.65 | -4193.62 | 0.0743 | 0.0780 | 0.06 | 0.814 | 0 |
| ***SH3PXD2B*** | ***-24447.81*** | ***-24443.46*** | ***0.1603*** | ***0.1183*** | ***8.71*** | ***0.003*** | ***1*** |
| ***SPP2*** | ***-7264.52*** | ***-7261.86*** | ***0.2918*** | ***0.4520*** | ***5.32*** | ***0.021*** | ***1*** |
| *SRD5A1* | -4345.07 | -4345.01 | 0.2304 | 0.2098 | 0.12 | 0.732 | 0 |
| *SRGN* | -3843.52 | -3841.46 | 0.2102 | 0.1212 | 4.13 | 0.042 | 0 |
| *SULF1* | -17554.42 | -17553.24 | 0.0833 | 0.0678 | 2.36 | 0.125 | 0 |
| ***SULF2*** | ***-19135.59*** | ***-19132.50*** | ***0.0630*** | ***0.0430*** | ***6.18*** | ***0.013*** | ***1*** |
| ***SYK*** | ***-8616.47*** | ***-8583.54*** | ***0.0651*** | ***0.2070*** | ***65.86*** | ***0.000*** | ***1*** |
| ***TCF7L2*** | ***-5655.00*** | ***-5646.31*** | ***0.1123*** | ***0.2547*** | ***17.38*** | ***0.000*** | ***1*** |
| *TFRC* | -32110.43 | -32109.79 | 0.4691 | 0.5141 | 1.28 | 0.257 | 0 |
| *TGFB3* | -5431.84 | -5429.82 | 0.0188 | 0.0291 | 4.04 | 0.044 | 0 |
| ***TNFAIP3*** | ***-23443.12*** | ***-23436.23*** | ***0.1779*** | ***0.2582*** | ***13.77*** | ***0.000*** | ***1*** |
| *TPH1* | -8989.13 | -8988.72 | 0.0935 | 0.0781 | 0.82 | 0.366 | 0 |
| *TPP1* | -17754.24 | -17754.24 | 0.4131 | 0.4204 | 0.02 | 0.897 | 0 |
| *TRAF6* | -14122.88 | -14121.85 | 0.0688 | 0.0548 | 2.07 | 0.150 | 0 |
| *TUFT1* | -4587.75 | -4587.43 | 0.0545 | 0.0428 | 0.63 | 0.426 | 0 |
| *VEGFA* | -1916.55 | -1916.47 | 0.2165 | 0.1721 | 0.16 | 0.691 | 0 |
