# Supplementary material for: Bone-associated gene evolution and the origin of flight in birds
Source: BMC Genomics. 2016 May 18;17:371. doi: 10.1186/s12864-016-2681-7 (PMC4870793; doi:10.1186/s12864-016-2681-7)
Supplement: Additional file 13: Table S10. — Branch model for mammals. Genes without flying mammals present in the alignment are marked (###). Bold represents statistical significance (p < 0.05). Q-value estimations for multiple testing are represented as positive selected (1) and negative selected (0). (DOC 114 kb) [file 12864_2016_2681_MOESM13_ESM.doc]

# Additional file 13: Table S10 - Branch model for mammals. Genes without flying mammals present in the alignment are marked (###). Bold represents statistical significance (p<0.05). Q-value estimations for multiple testing are represented as positive selected (1) and negative selected (0).

| Gene | Model One-Ratio | Model Two-Ratio | Background Branch | Foreground Branch | LRT | p-value | q-value |
| --- | --- | --- | --- | --- | --- | --- | --- |
| *ACVR2A* | -9750.79 | -9749.55 | 0.0507 | 0.0854 | 2.47 | 0.1157 | 0 |
| ***ACVR2B*** | ***-9584.95*** | ***-9564.22*** | ***0.0212*** | ***0.0797*** | ***41.46*** | ***0*** | ***1*** |
| *ADAM8* | -38815.55 | -38815.54 | 0.2689 | 0.2724 | 0.02 | 0.8927 | 0 |
| ***AHSG*** | ***-23765.4*** | ***-23760.95*** | ***0.4706*** | ***0.6933*** | ***8.9*** | ***0.0029*** | ***1*** |
| *ANKH* | -11855.21 | -11854.34 | 0.0286 | 0.0401 | 1.74 | 0.1871 | 0 |
| ***AQP1*** | ***-6611.57*** | ***-6609.08*** | ***0.0544*** | ***0.0957*** | ***4.99*** | ***0.0255*** | ***1*** |
| *ASPN* | -10844.52 | -10843.25 | 0.1075 | 0.1611 | 2.54 | 0.1109 | 0 |
| *BCOR* | -54967.63 | -54966.19 | 0.1179 | 0.102 | 2.88 | 0.0896 | 0 |
| ***BMP2*** | ***-10613.16*** | ***-10610.29*** | ***0.103*** | ***0.0518*** | ***5.74*** | ***0.0165*** | ***1*** |
| ***BMP7*** | ***-9473.43*** | ***-9470.18*** | ***0.0262*** | ***0.0483*** | ***6.5*** | ***0.0108*** | ***1*** |
| ***BMPR1A*** | ***-10887.29*** | ***-10884.97*** | ***0.042*** | ***0.0918*** | ***4.64*** | ***0.0312*** | ***1*** |
| ***CA2*** | ***-10580.57*** | ***-10578.57*** | ***0.2658*** | ***0.1947*** | ***3.99*** | ***0.0457*** | ***1*** |
| *CARM1* | ### |  |  |  |  |  |  |
| *CBS* | -18230.99 | -18230.11 | 0.0974 | 0.1175 | 1.76 | 0.1843 | 0 |
| *CD38* | -16217.98 | -16217.76 | 0.532 | 0.5905 | 0.46 | 0.4985 | 0 |
| *CDX1* | -6379.04 | -6378.62 | 0.1376 | 0.1684 | 0.84 | 0.3587 | 0 |
| *CER1* | -10914.24 | -10914.23 | 0.3394 | 0.3323 | 0.01 | 0.91 | 0 |
| ***CITED2*** | ***-3334.56*** | ***-3322.38*** | ***0.0653*** | ***0.3004*** | ***24.36*** | ***0*** | ***1*** |
| ***COL2A1*** | ***-34176.27*** | ***-34172.39*** | ***0.1171*** | ***0.0711*** | ***7.78*** | ***0.0053*** | ***1*** |
| *CREB3L1* | -12925.83 | -12924 | 0.0796 | 0.0487 | 3.67 | 0.0553 | 0 |
| *CTHRC1* | -6754 | -6753.97 | 0.0969 | 0.106 | 0.05 | 0.815 | 0 |
| *CTSK* | -9294.53 | -9293.95 | 0.1266 | 0.0967 | 1.16 | 0.2822 | 0 |
| *DLX5* | -5210.04 | -5210.01 | 0.0642 | 0.0579 | 0.06 | 0.8083 | 0 |
| ***DUOX2*** | ***-54148.58*** | ***-54143.35*** | ***0.1567*** | ***0.21*** | ***10.45*** | ***0.0012*** | ***1*** |
| *DYM* | -16342.33 | -16341.81 | 0.0791 | 0.0979 | 1.04 | 0.3081 | 0 |
| *EIF2AK3* | -36979.36 | -36968.58 | 0.1287 | 0.2287 | 21.57 | 0 | 1 |
| *FBXL15* | -9545.87 | -9545.81 | 0.13 | 0.1435 | 0.13 | 0.7214 | 0 |
| ***FGF23*** | ***-9396.6*** | ***-9389.34*** | ***0.1229*** | ***0.2314*** | ***14.52*** | ***0.0001*** | ***1*** |
| *FGF8* | -4540.68 | -4540.05 | 0.0923 | 0.0549 | 1.27 | 0.2592 | 0 |
| *GAS6* | -27414.98 | -27413.37 | 0.1544 | 0.1962 | 3.22 | 0.0727 | 0 |
| *GHR* | -21977.59 | -21977.53 | 0.2962 | 0.3089 | 0.11 | 0.7427 | 0 |
| *GPLD1* | -33064.51 | -33063.73 | 0.2315 | 0.1879 | 1.55 | 0.2129 | 0 |
| *GPM6B* | -8218.49 | -8216.76 | 0.1033 | 0.1641 | 3.46 | 0.0628 | 0 |
| ***GREM1*** | ***-3819.46*** | ***-3812.43*** | ***0.0214*** | ***0.0926*** | ***14.07*** | ***0.0002*** | ***1*** |
| ***HOXA11*** | ***-6697.5*** | ***-6695.28*** | ***0.1398*** | ***0.2135*** | ***4.42*** | ***0.0355*** | ***1*** |
| *HOXB4* | -4825.18 | -4823.73 | 0.12 | 0.037 | 2.9 | 0.0884 | 0 |
| ***HOXD11*** | ***-7337.44*** | ***-7332.39*** | ***0.1666*** | ***0.2914*** | ***10.11*** | ***0.0015*** | ***1*** |
| *HSD17B2* | -18921.52 | -18920.57 | 0.3718 | 0.4607 | 1.9 | 0.1684 | 0 |
| *IAPP* | -3292.77 | -3291.33 | 0.3845 | 0.7228 | 2.88 | 0.0896 | 0 |
| *IFITM5* | ### |  |  |  |  |  |  |
| *IGF1* | -4716.72 | -4716.18 | 0.1939 | 0.2777 | 1.08 | 0.2988 | 0 |
| *IHH* | -10287.35 | -10287.35 | 0.0679 | 0.0694 | 0.01 | 0.9383 | 0 |
| *IL6* | -12481.92 | -12481.91 | 0.7025 | 0.6792 | 0.03 | 0.8729 | 0 |
| *IL7* | -3236.87 | -3236.84 | 0.6032 | 0.6488 | 0.05 | 0.8277 | 0 |
| ***INPP5D*** | ***-40139.73*** | ***-40135.63*** | ***0.1332*** | ***0.1775*** | ***8.19*** | ***0.0042*** | ***1*** |
| ***KLF10*** | ***-14207.6*** | ***-14203.22*** | ***0.1496*** | ***0.2606*** | ***8.76*** | ***0.0031*** | ***1*** |
| *LRP6* | -33752.84 | -33752.18 | 0.0424 | 0.0536 | 1.33 | 0.2485 | 0 |
| *LRRC17* | -14573.29 | -14573.27 | 0.1411 | 0.1353 | 0.04 | 0.8327 | 0 |
| ***MC4R*** | ***-8135.01*** | ***-8122.71*** | ***0.0475*** | ***0.1377*** | ***24.59*** | ***0*** | ***1*** |
| ***MEF2A*** | ***-15390.23*** | ***-15374.17*** | ***0.1364*** | ***0.0564*** | ***32.11*** | ***0*** | ***1*** |
| *MEF2C* | -8439.71 | -8439.41 | 0.1372 | 0.1659 | 0.59 | 0.4418 | 0 |
| ***MEPE*** | ***-27855.67*** | ***-27851.25*** | ***0.5039*** | ***0.3441*** | ***8.84*** | ***0.003*** | ***1*** |
| *MGP* | -3828.38 | -3826.95 | 0.1956 | 0.3446 | 2.86 | 0.091 | 0 |
| *MITF* | -11836.16 | -11834.61 | 0.0794 | 0.1092 | 3.1 | 0.0785 | 0 |
| ***MMP2*** | ***-19106.74*** | ***-19085.03*** | ***0.0682*** | ***0.1614*** | ***43.42*** | ***0*** | ***1*** |
| *MSX1* | -6840.9 | -6840.89 | 0.0709 | 0.0671 | 0.02 | 0.8957 | 0 |
| *NBR1* | -26784.06 | -26783.07 | 0.2599 | 0.2079 | 1.97 | 0.1608 | 0 |
| ***NCDN*** | ***-15754.48*** | ***-15750.93*** | ***0.0494*** | ***0.0205*** | ***7.1*** | ***0.0077*** | ***1*** |
| ***NF1*** | ***-52208*** | ***-52203.46*** | ***0.0365*** | ***0.0574*** | ***9.1*** | ***0.0026*** | ***1*** |
| ***NOX4*** | ***-15140.69*** | ***-15136.28*** | ***0.1645*** | ***0.2698*** | ***8.82*** | ***0.003*** | ***1*** |
| ***OSR2*** | ***-5900.05*** | ***-5894.82*** | ***0.0703*** | ***0.1542*** | ***10.46*** | ***0.0012*** | ***1*** |
| *P2RX7* | -19177.78 | -19177.6 | 0.2063 | 0.1818 | 0.37 | 0.5442 | 0 |
| *PAPSS2* | -20583.03 | -20582.94 | 0.0988 | 0.0915 | 0.17 | 0.6758 | 0 |
| ***PKDCC*** | ***-9721.1*** | ***-9719.19*** | ***0.0817*** | ***0.1285*** | ***3.82*** | ***0.0507*** | ***1*** |
| ***PLA2G4A*** | ***-19685.71*** | ***-19679.11*** | ***0.068*** | ***0.1292*** | ***13.2*** | ***0.0003*** | ***1*** |
| *PLXNB1* | -64864.6 | -64863.03 | 0.1416 | 0.1746 | 3.13 | 0.0767 | 0 |
| *PTGER4* | -14372.1 | -14371.13 | 0.0966 | 0.1221 | 1.96 | 0.162 | 0 |
| *PTH* | -4372.73 | -4371.71 | 0.3174 | 0.6133 | 2.03 | 0.1537 | 0 |
| ***PTK2B*** | ***-26169.58*** | ***-26166.87*** | ***0.0557*** | ***0.0818*** | ***5.43*** | ***0.0198*** | ***1*** |
| *PTN* | -6069.17 | -6069.07 | 0.1715 | 0.1924 | 0.21 | 0.6453 | 0 |
| *SBDS* | -4311.49 | -4310.07 | 0.0436 | 0.0109 | 2.84 | 0.0922 | 0 |
| *SFRP1* | -5941.62 | -5941.57 | 0.0452 | 0.0405 | 0.1 | 0.7528 | 0 |
| *SFRP2* | ### |  |  |  |  |  |  |
| *SH3PXD2B* | -25719.88 | -25719.68 | 0.1262 | 0.1411 | 0.39 | 0.532 | 0 |
| *SPP2* | -10155.77 | -10155.42 | 0.3564 | 0.4205 | 0.71 | 0.4007 | 0 |
| *SRD5A1* | -11004.89 | -11004.8 | 0.2857 | 0.2658 | 0.18 | 0.671 | 0 |
| *SRGN* | -7677.94 | -7677.94 | 0.4112 | 0.4167 | 0 | 0.9575 | 0 |
| *SULF1* | -27848.1 | -27846.83 | 0.0935 | 0.0727 | 2.55 | 0.11 | 0 |
| *SULF2* | -21842.44 | -21842.1 | 0.0512 | 0.0598 | 0.69 | 0.4074 | 0 |
| ***SYK*** | ***-19535.2*** | ***-19531.78*** | ***0.0709*** | ***0.0461*** | ***6.83*** | ***0.009*** | ***1*** |
| ***TCF7L2*** | ***-13560.88*** | ***-13553.89*** | ***0.1371*** | ***0.042*** | ***13.98*** | ***0.0002*** | ***1*** |
| ***TFRC*** | ***-32317.76*** | ***-32307.11*** | ***0.3226*** | ***0.5521*** | ***21.31*** | ***0*** | ***1*** |
| ***TGFB3*** | ***-8801.84*** | ***-8797.75*** | ***0.0546*** | ***0.1086*** | ***8.18*** | ***0.0042*** | ***1*** |
| *TNFAIP3* | -27479.46 | -27478.07 | 0.1088 | 0.1397 | 2.77 | 0.0958 | 0 |
| *TPH1* | -10926.03 | -10925.17 | 0.1262 | 0.1707 | 1.72 | 0.1899 | 0 |
| *TPP1* | -15351.68 | -15351.25 | 0.1976 | 0.2346 | 0.85 | 0.3556 | 0 |
| *TRAF6* | -16310.82 | -16309.82 | 0.1266 | 0.1647 | 2 | 0.1568 | 0 |
| *TUFT1* | -12809.61 | -12809.5 | 0.2115 | 0.2299 | 0.22 | 0.6399 | 0 |
| *VEGFA* | -9874.3 | -9873.3 | 0.3447 | 0.2335 | 2 | 0.157 | 0 |
