# Supplementary material for: Bone-associated gene evolution and the origin of flight in birds
Source: BMC Genomics. 2016 May 18;17:371. doi: 10.1186/s12864-016-2681-7 (PMC4870793; doi:10.1186/s12864-016-2681-7)
Supplement: Additional file 14: Figure S4. — Body mass association with ω (dN/dS). Avian cladogram showing from CoEvol, the labels are the estimated ω (minimum maximum) for each branch on top and the estimated weight (minimum maximum). (DOC 423 kb) [file 12864_2016_2681_MOESM14_ESM.doc]

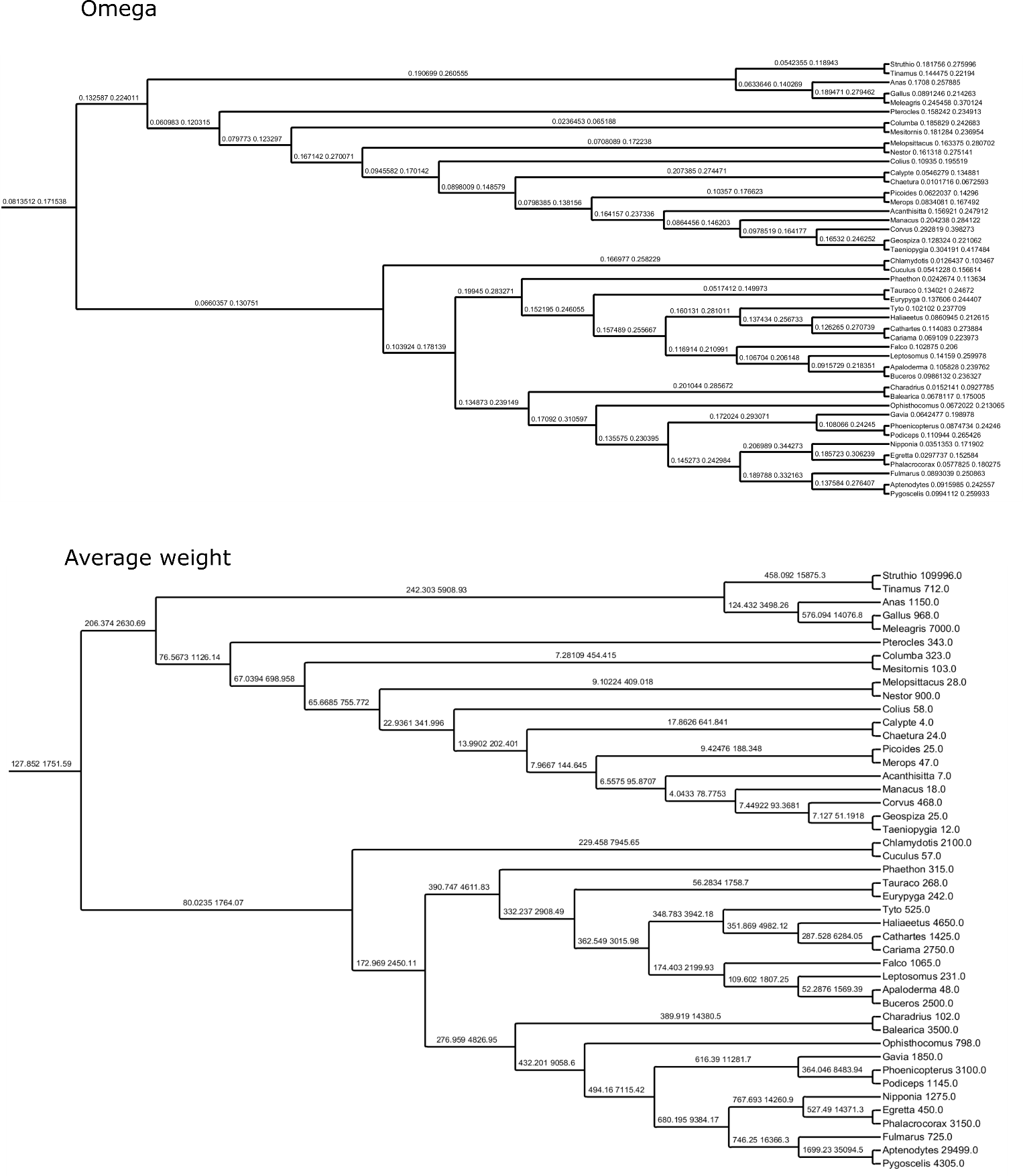


# Additional file 14: Figure S4 - Body mass association with ω (d_N_/d_S_). Avian cladogram showing from CoEvol, the labels are the estimated ω (minimum maximum) for each branch on top and the estimated weight (minimum maximum).
