# Supplementary material for: Bone-associated gene evolution and the origin of flight in birds
Source: BMC Genomics. 2016 May 18;17:371. doi: 10.1186/s12864-016-2681-7 (PMC4870793; doi:10.1186/s12864-016-2681-7)
Supplement: Additional file 15: Table S11. — Covariance between dS, ω (dN/dS), gc content, and the three body mass measures (minimum, maximum and average) in 45 bird genomes using gene-based tree. The upper triangle shows the values obtained for all birds and the lower triangle excluding flightless birds. Each cell represent the covariance values and posterior probability are the bracketed values, posterior probability (** - < = 0.025 or > =0.975; * - < =0.05 or > =0.95) are highlighted in bold for the statistically significant correlations. (DOC 35 kb) [file 12864_2016_2681_MOESM15_ESM.doc]

# Additional file 15: Table S11 - Covariance between dS, ω (d_N_/d_S_), gc content, and the three body mass measures (minimum, maximum and average) in 45 bird genomes using gene-based tree. The upper triangle shows the values obtained for all birds and the lower triangle excluding flightless birds. Each cell represent the covariance values and posterior probability are the bracketed values, posterior probability (** - <= 0.025 or >=0.975; * - <=0.05 or >=0.95) are highlighted in bold for the statistically significant correlations.

|  | Avian dataset | | | | | |
| --- | --- | --- | --- | --- | --- | --- |
|  | d_S_ | ω | gc | Minimum weight | Maximum weight | Average weight |
| d_S_ | - | -0.2025  (0.28) | 0.2535  (0.75) | -0.407  (0.0655) | -0.411  (0.057) | -0.408  (0.0615) |
| ω | **-0.5135**  **(0.042)*** | - | **-0.8655**  **(0.001075)**** | **0.5325**  **(0.97)*** | **0.4875**  **(0.95)*** | **0.5065**  **(0.955)*** |
| gc | 0.519  (0.945) | **-0.81**  **(0.004)**** | - | **-0.576**  **(0.0285)*** | -0.531  (0.051) | **-0.55**  **(0.043)*** |
| Minimum  weight | -0.3725  (0.075) | 0.209  (0.745) | -0.2155  (0.275) | - | **0.9905**  **(1)**** | **0.9955**  **(1)**** |
| Maximum  weight | -0.3675  (0.0795) | 0.148  (0.68) | -0.1575  (0.335) | **0.9845**  **(1)**** | - | **0.9965**  **(1)**** |
| Average  weight | -0.3735  (0.076) | 0.1735  (0.705) | -0.183  (0.305) | **0.9935**  **(1)**** | **0.998**  **(1)**** | - |
