# Supplementary material for: Bone-associated gene evolution and the origin of flight in birds
Source: BMC Genomics. 2016 May 18;17:371. doi: 10.1186/s12864-016-2681-7 (PMC4870793; doi:10.1186/s12864-016-2681-7)
Supplement: Additional file 16: Table S12. — Covariance between dS, ω (dN/dS), gc content, and the three body mass measures (minimum, maximum and average) in 39 mammalian genomes using gene-based tree. The upper triangle shows the values obtained for all mammals and the lower triangle excluding bats. Each cell represent the covariance values and posterior probability are the bracketed values, posterior probability (** - < = 0.025 or > =0.975; * - < =0.05 or > =0.95) are highlighted in bold for the statistically significant correlations. (DOC 35 kb) [file 12864_2016_2681_MOESM16_ESM.doc]

# Additional file 16: Table S12 - Covariance between dS, ω (d_N_/d_S_), gc content, and the three body mass measures (minimum, maximum and average) in 39 mammalian genomes using gene-based tree. The upper triangle shows the values obtained for all mammals and the lower triangle excluding bats. Each cell represent the covariance values and posterior probability are the bracketed values, posterior probability (** - <= 0.025 or >=0.975; * - <=0.05 or >=0.95) are highlighted in bold for the statistically significant correlations.

|  | Mammalian dataset | | | | | |
| --- | --- | --- | --- | --- | --- | --- |
|  | d_S_ | ω | gc | Minimum weight | Maximum weight | Average weight |
| d_S_ | - | **-0.5315**  **(0.0245)**** | 0.331  (0.935) | **-0.579**  **(0.00655)**** | **-0.533**  **(0.0094)**** | **-0.545**  **(0.00865)**** |
| ω | **-0.532**  **(0.0195)**** | - | **-0.481**  **(0.00785)**** | **0.5455**  **(0.99)**** | **0.529**  **(0.985)**** | **0.539**  **(0.99)**** |
| gc | 0.294  (0.915) | **-0.4565**  **(0.009275)**** | - | **-0.49**  **(0.017)**** | **-0.521**  **(0.0135)**** | **-0.527**  **(0.012)**** |
| Minimum  weight | **-0.561**  **(0.00555)**** | **0.578**  **(0.99)**** | **-0.4235**  **(0.046)*** | - | **0.9655**  **(1)**** | **0.9755**  **(1)**** |
| Maximum  weight | **-0.5185**  **(0.0113)**** | **0.566**  **(0.99)**** | **-0.4795**  **(0.0275)*** | **0.959**  **(1)**** | - | **0.999**  **(1)**** |
| Average  weight | **-0.531**  **(0.0085)**** | **0.5765**  **(0.99)**** | **-0.481**  **(0.024)**** | **0.971**  **(1)**** | **0.998**  **(1)**** | - |
