# Supplementary figures and images for: Bone-associated gene evolution and the origin of flight in birds
Source: BMC Genomics. 2016 May 18;17:371. doi: 10.1186/s12864-016-2681-7 (PMC4870793; doi:10.1186/s12864-016-2681-7)

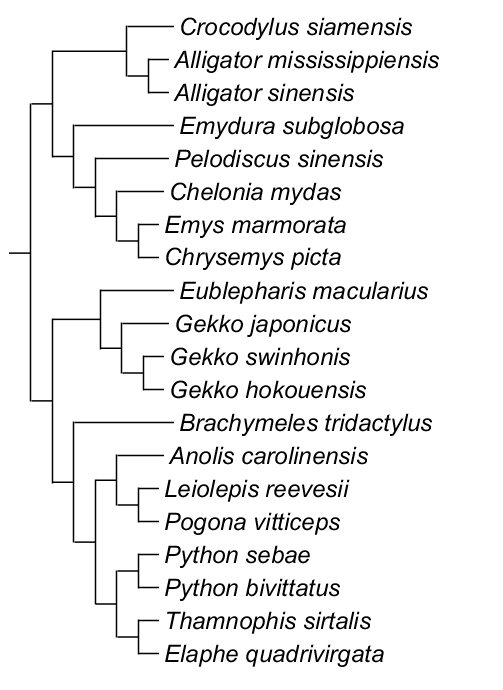


# Additional file 17: Figure S6 - Phylogenetic trees of reptiles used in CODEML analysis.

Supplement: Additional file 17: Figure S6. — Phylogenetic trees of reptiles used in CODEML analysis. (DOC 90 kb) [file 12864_2016_2681_MOESM17_ESM.doc]
