# Supplementary material for: Bone-associated gene evolution and the origin of flight in birds
Source: BMC Genomics. 2016 May 18;17:371. doi: 10.1186/s12864-016-2681-7 (PMC4870793; doi:10.1186/s12864-016-2681-7)
Supplement: Additional file 18: Figure S5. — Avian and Mammalian phylogenetic trees used in CODEML analysis. Lineages of flightless birds are highlighted in red, while flying mammals are highlighted in blue. (DOC 339 kb) [file 12864_2016_2681_MOESM18_ESM.doc]

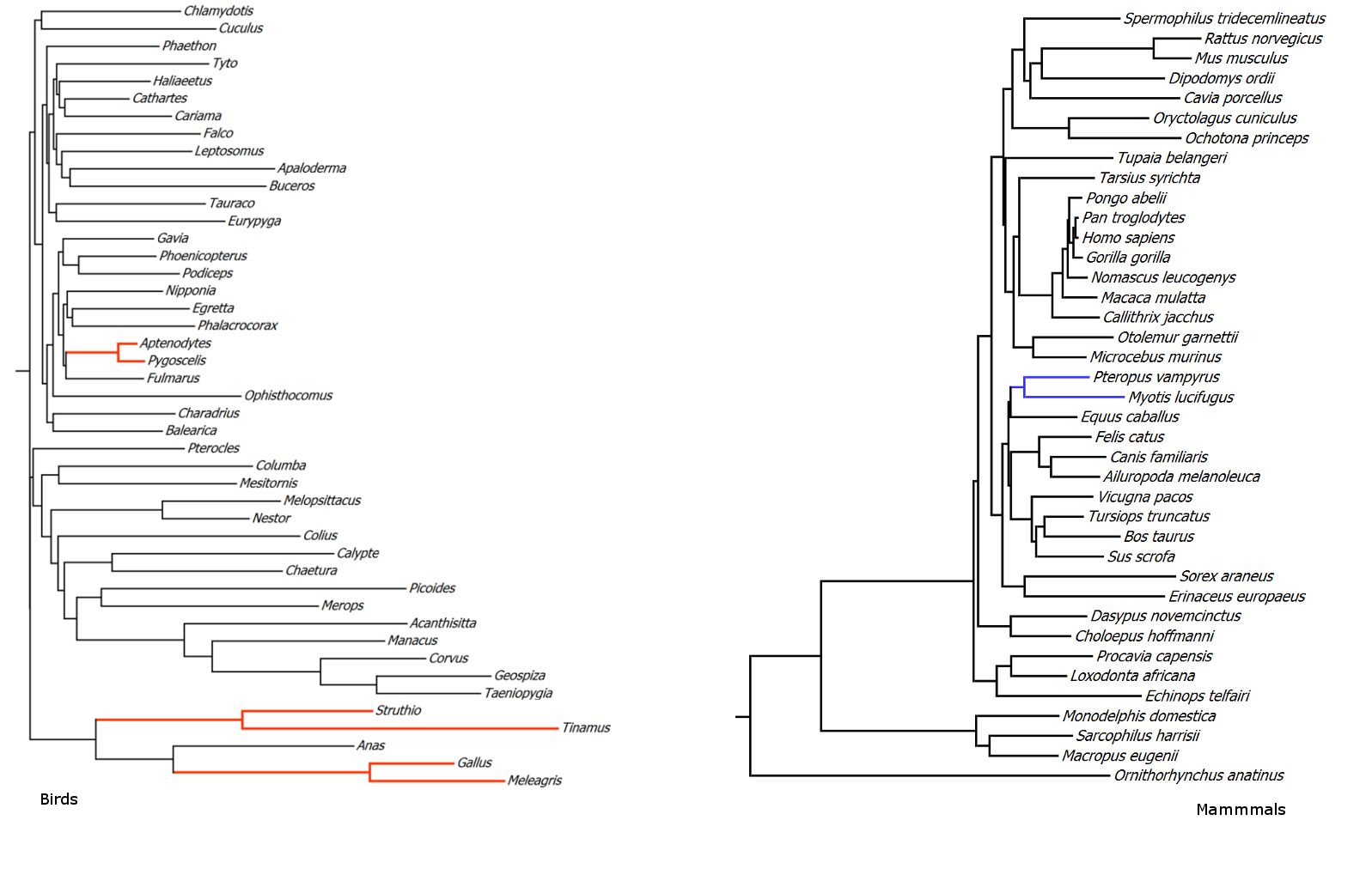


Additional file 18: Figure S5 – Avian and Mammalian phylogenetic trees used in CODEML analysis. Lineages of flightless birds are highlighted in red, while flying mammals are highlighted in blue.
