# Supplementary material for: Bone-associated gene evolution and the origin of flight in birds
Source: BMC Genomics. 2016 May 18;17:371. doi: 10.1186/s12864-016-2681-7 (PMC4870793; doi:10.1186/s12864-016-2681-7)
Supplement: Additional file 19: Table S13. — Estimation of dN and dS for each branch under Model 0. For each branch, average of dN and dS and the corresponding standard deviation. (DOC 165 kb) [file 12864_2016_2681_MOESM19_ESM.doc]

# Additional file 19: Table S14 – Estimation of d_N_ and d_S_ for each branch under Model 0. For each branch, average of d_N_ and d_S_ and the corresponding standard deviation.

|  | Birds | | | | Reptiles | | | | Mammals | | | |  |
| --- | --- | --- | --- | --- | --- | --- | --- | --- | --- | --- | --- | --- | --- |
| Gene | dN | standard  deviation | dS | standard  deviation | dN | standard  deviation | dS | standard  deviation | dN | standard  deviation | dS | standard  deviation |  |
| *ACVR2A* | 0.00145 | 0.00192 | 0.86930 | 0.11626 | 0.00313 | 0.00241 | 0.22310 | 0.08526 | 0.00388 | 0.00551 | 0.79640 | 0.10604 |  |
| *ACVR2B* | 0.00116 | 0.00118 | 0.21030 | 0.04242 | 0.01672 | 0.01889 | 0.63820 | 0.16885 | 0.00346 | 0.00394 | 0.63970 | 0.15237 |  |
| *ADAM8* | 0.04717 | 0.11439 | 3.15170 | 0.67213 | 0.04401 | 0.03171 | 0.27040 | 0.11256 | 0.06039 | 0.04979 | 0.62590 | 0.18497 |  |
| *AHSG* | 0.02630 | 0.02515 | 0.22740 | 0.04769 | 0.14830 | 0.13017 | 0.79880 | 0.26179 | 0.06366 | 0.05230 | 0.43520 | 0.10847 |  |
| *ANKH* | 0.00227 | 0.00209 | 0.13600 | 0.03077 | 0.00365 | 0.00277 | 0.24440 | 0.08818 | 0.00377 | 0.00384 | 0.82290 | 0.13050 |  |
| *AQP1* | 0.00323 | 0.00290 | 0.18840 | 0.04598 | 0.01988 | 0.01611 | 0.62610 | 0.21348 | 0.01132 | 0.01073 | 0.92900 | 0.18839 |  |
| *ASPN* | 0.00946 | 0.00811 | 0.19900 | 0.04966 | 0.01809 | 0.01381 | 0.33020 | 0.10960 | 0.01146 | 0.01285 | 0.63790 | 0.11759 |  |
| *BCOR* | 0.00813 | 0.00839 | 0.30460 | 0.06350 | 0.15346 | 0.31722 | 5.54370 | 1.77774 | 0.01331 | 0.01308 | 0.48080 | 0.11211 |  |
| *BMP2* | 0.00156 | 0.00188 | 0.09030 | 0.01717 | 0.01498 | 0.01234 | 0.35950 | 0.09797 | 0.00939 | 0.01069 | 0.63060 | 0.10649 |  |
| *BMP7* | 0.00151 | 0.00149 | 0.25880 | 0.05327 | 0.02914 | 0.03828 | 0.80850 | 0.23502 | 0.00500 | 0.00617 | 1.28870 | 0.22470 |  |
| *BMPR1A* | 0.00201 | 0.00198 | 0.18200 | 0.04539 | 0.04739 | 0.04056 | 0.05200 | 0.01638 | 0.00553 | 0.00803 | 0.90440 | 0.18655 |  |
| *CA2* | 0.00708 | 0.00674 | 0.20940 | 0.05105 | 0.03512 | 0.01681 | 0.21020 | 0.06026 | 0.03348 | 0.04027 | 0.91110 | 0.15655 |  |
| *CARM1* | 0.01978 | 0.04500 | 1.13730 | 0.19730 | 0.04100 | 0.09992 | 5.06030 | 1.36540 | 0.01478 | 0.01610 | 0.65850 | 0.18186 |  |
| *CBS* | 0.01425 | 0.01533 | 0.21280 | 0.04974 | 0.04805 | 0.05636 | 0.77780 | 0.29866 | 0.01940 | 0.01680 | 0.63750 | 0.17005 |  |
| *CD38* | 0.01386 | 0.01522 | 0.39300 | 0.05526 | 0.04854 | 0.04245 | 0.59010 | 0.16644 | 0.06763 | 0.06459 | 0.50760 | 0.12054 |  |
| *CDX1* | 0.00710 | 0.00971 | 1.11720 | 0.15865 | 0.12818 | 0.14527 | 2.48100 | 0.98880 | 0.02217 | 0.03352 | 1.41980 | 0.23845 |  |
| *CER1* | 0.02008 | 0.01889 | 0.19170 | 0.04464 | 0.08163 | 0.05454 | 0.35890 | 0.12238 | 0.03640 | 0.03406 | 0.41750 | 0.10051 |  |
| *CITED2* | 0.00975 | 0.01337 | 0.32300 | 0.09717 | 0.01924 | 0.02225 | 0.83670 | 0.24177 | 0.00704 | 0.00812 | 0.40640 | 0.10211 |  |
| *COL2A1* | 0.04285 | 0.14126 | 6.58220 | 1.38857 | 0.04430 | 0.09840 | 2.80010 | 0.75284 | 0.00702 | 0.00699 | 0.25910 | 0.06090 |  |
| *CREB3L1* | 0.00603 | 0.00562 | 0.11190 | 0.02829 | 0.01639 | 0.01133 | 0.26680 | 0.07909 | 0.01088 | 0.01317 | 1.00060 | 0.16882 |  |
| *CTHRC1* | 0.00331 | 0.00398 | 0.60620 | 0.08601 | 0.05066 | 0.03920 | 0.51630 | 0.14469 | 0.01045 | 0.01380 | 0.94050 | 0.14199 |  |
| *CTSK* | 0.04712 | 0.07496 | 4.41980 | 0.91420 | 0.02867 | 0.01298 | 0.83680 | 0.23103 | 0.01205 | 0.01369 | 0.58040 | 0.10961 |  |
| *DLX5* | 0.03315 | 0.03942 | 0.43210 | 0.13335 | 0.02944 | 0.03265 | 0.97470 | 0.29215 | 0.00454 | 0.00552 | 0.46860 | 0.08648 |  |
| *DUOX2* | 0.01410 | 0.01143 | 0.26390 | 0.07206 | 0.03586 | 0.02531 | 0.54260 | 0.16732 | 0.01982 | 0.01774 | 0.44020 | 0.11128 |  |
| *DYM* | 0.01098 | 0.04910 | 7.23050 | 1.08110 | 0.00770 | 0.00541 | 0.25380 | 0.08017 | 0.00726 | 0.00799 | 0.53730 | 0.09997 |  |
| *EIF2AK3* | 0.00563 | 0.00863 | 0.73200 | 0.08921 | 0.02322 | 0.01650 | 0.30480 | 0.10041 | 0.01426 | 0.01413 | 0.53530 | 0.10691 |  |
| *FBXL15* | 0.00528 | 0.00648 | 0.56240 | 0.08213 | 0.03439 | 0.02587 | 0.48930 | 0.19086 | 0.01808 | 0.01982 | 0.58020 | 0.15187 |  |
| *FGF23* | 0.01083 | 0.00977 | 0.26480 | 0.05919 | 0.05338 | 0.05842 | 1.08880 | 0.33502 | 0.02884 | 0.03262 | 1.12790 | 0.24909 |  |
| *FGF8* | 0.00437 | 0.00667 | 0.81730 | 0.10750 | 0.01348 | 0.01127 | 0.54670 | 0.18516 | 0.01467 | 0.03103 | 2.06090 | 0.34274 |  |
| *GAS6* | 0.00838 | 0.00743 | 0.20780 | 0.04246 | 0.03805 | 0.02751 | 0.36430 | 0.10459 | 0.02900 | 0.02646 | 0.98260 | 0.16926 |  |
| *GHR* | 0.01041 | 0.00927 | 0.13730 | 0.03731 | 0.03228 | 0.02943 | 0.35050 | 0.09177 | 0.02333 | 0.02447 | 0.38230 | 0.08240 |  |
| *GPLD1* | 0.01479 | 0.01545 | 0.24570 | 0.05233 | 0.05448 | 0.07326 | 1.01620 | 0.27411 | 0.02992 | 0.03099 | 0.75170 | 0.13457 |  |
| *GPM6B* | 0.00078 | 0.00102 | 0.21440 | 0.03237 | 0.04093 | 0.11501 | 3.92360 | 1.07082 | 0.01001 | 0.01245 | 0.47930 | 0.11719 |  |
| *GREM1* | 0.00322 | 0.00391 | 0.39910 | 0.06476 | 0.02011 | 0.01938 | 0.58640 | 0.16641 | 0.00273 | 0.00248 | 0.35740 | 0.09745 |  |
| *HOXA11* | 0.00829 | 0.01870 | 0.45380 | 0.07902 | 0.01592 | 0.01595 | 0.46960 | 0.13893 | 0.01132 | 0.01820 | 0.58910 | 0.12408 |  |
| *HOXB4* | 0.00811 | 0.04497 | 1.59030 | 0.18723 | 0.01495 | 0.01533 | 0.44730 | 0.12809 | 0.01258 | 0.02523 | 1.40530 | 0.21434 |  |
| *HOXD11* | 0.00249 | 0.00349 | 0.14380 | 0.02945 | 0.03581 | 0.03196 | 0.56370 | 0.18556 | 0.04173 | 0.07709 | 1.64880 | 0.42340 |  |
| *HSD17B2* | 0.01755 | 0.01602 | 0.24540 | 0.05884 | 0.06729 | 0.05412 | 0.41400 | 0.16780 | 0.05169 | 0.05267 | 0.79520 | 0.14004 |  |
| *IAPP* | 0.01211 | 0.01163 | 0.14330 | 0.03790 | 0.05322 | 0.06146 | 0.57740 | 0.18284 | 0.04549 | 0.04751 | 0.60310 | 0.11950 |  |
| *IFITM5* | 0.04628 | 0.05847 | 1.98050 | 0.46717 | 0.03027 | 0.02344 | 0.45110 | 0.13948 | 0.01965 | 0.02561 | 1.47250 | 0.25172 |  |
| *IGF1* | 0.00095 | 0.00111 | 0.07130 | 0.01667 | 0.01576 | 0.01513 | 0.33160 | 0.09727 | 0.01744 | 0.01978 | 0.47030 | 0.10026 |  |
| *IHH* | 0.02207 | 0.05146 | 3.04220 | 0.47977 | 0.05066 | 0.02952 | 0.70000 | 0.28948 | 0.00850 | 0.00794 | 0.59230 | 0.11683 |  |
| *IL6* | 0.02107 | 0.02029 | 0.28480 | 0.06508 | 0.08768 | 0.05271 | 0.42480 | 0.12840 | 0.08252 | 0.09012 | 0.59450 | 0.12850 |  |
| *IL7* | 0.01213 | 0.01278 | 0.16230 | 0.03638 | 0.06967 | 0.03734 | 0.08780 | 0.04456 | 0.04992 | 0.05117 | 0.28930 | 0.08423 |  |
| *INPP5D* | 0.00782 | 0.00866 | 0.30410 | 0.06564 | 0.02970 | 0.02223 | 0.30730 | 0.10516 | 0.02448 | 0.03101 | 1.50110 | 0.22828 |  |
| *KLF10* | 0.02085 | 0.02035 | 0.50510 | 0.08665 | 0.04488 | 0.03731 | 0.32130 | 0.11916 | 0.01661 | 0.02171 | 0.81430 | 0.14096 |  |
| *LRP6* | 0.00138 | 0.00142 | 0.24590 | 0.05631 | 0.01026 | 0.00695 | 0.33520 | 0.11439 | 0.00354 | 0.00401 | 0.57920 | 0.09386 |  |
| *LRRC17* | 0.00631 | 0.00646 | 0.17390 | 0.04167 | 0.02737 | 0.02624 | 0.47560 | 0.14842 | 0.01575 | 0.01811 | 0.69470 | 0.12860 |  |
| *MC4R* | 0.00235 | 0.00226 | 0.14160 | 0.03679 | 0.01285 | 0.00979 | 0.44700 | 0.14232 | 0.00734 | 0.00901 | 0.93200 | 0.16842 |  |
| *MEF2A* | 0.00446 | 0.00458 | 0.25930 | 0.05283 | 0.00733 | 0.00564 | 0.29480 | 0.08843 | 0.01167 | 0.01252 | 0.45230 | 0.09917 |  |
| *MEF2C* | 0.00222 | 0.00319 | 0.26730 | 0.03405 | 0.00475 | 0.00369 | 0.14610 | 0.04230 | 0.00586 | 0.00644 | 0.19890 | 0.04635 |  |
| *MEPE* | 0.03619 | 0.04010 | 0.46260 | 0.09927 | # | # | # | # | 0.06057 | 0.06722 | 0.62420 | 0.13656 |  |
| *MGP* | 0.02180 | 0.02333 | 0.25210 | 0.06626 | 0.03902 | 0.03033 | 0.44330 | 0.12095 | 0.02384 | 0.02132 | 0.51460 | 0.10538 |  |
| *MITF* | 0.00121 | 0.00124 | 0.21570 | 0.04367 | 0.04483 | 0.04170 | 0.73980 | 0.18902 | 0.00624 | 0.00652 | 0.40920 | 0.07953 |  |
| *MMP2* | 0.00266 | 0.00287 | 0.36360 | 0.05891 | 0.01315 | 0.01162 | 0.37570 | 0.14407 | 0.01077 | 0.01046 | 0.62800 | 0.14103 |  |
| *MSX1* | 0.01224 | 0.04223 | 8.23760 | 1.61805 | 0.03718 | 0.03218 | 0.96410 | 0.33051 | 0.00896 | 0.01015 | 0.68740 | 0.14337 |  |
| *NBR1* | 0.02269 | 0.02108 | 0.24160 | 0.05924 | 0.04923 | 0.03780 | 0.34050 | 0.13241 | 0.02442 | 0.03202 | 0.90070 | 0.12434 |  |
| *NCDN* | 0.01855 | 0.01687 | 0.34670 | 0.07007 | 0.03939 | 0.02659 | 0.34200 | 0.12137 | 0.01206 | 0.02591 | 3.74840 | 0.54462 |  |
| *NF1* | 0.00112 | 0.00123 | 0.36400 | 0.06850 | 0.00471 | 0.00441 | 0.39870 | 0.12375 | 0.00256 | 0.00244 | 0.30460 | 0.06522 |  |
| *NOX4* | 0.00745 | 0.00908 | 0.28300 | 0.04542 | 0.02320 | 0.01820 | 0.31490 | 0.11147 | 0.01349 | 0.01330 | 0.31240 | 0.07820 |  |
| *OSR2* | 0.01758 | 0.05155 | 3.15190 | 0.56960 | 0.01467 | 0.01597 | 0.72260 | 0.23276 | 0.00590 | 0.00846 | 0.45100 | 0.11038 |  |
| *P2RX7* | 0.05433 | 0.04304 | 0.18090 | 0.06090 | 0.04568 | 0.03987 | 0.42750 | 0.16035 | 0.02475 | 0.02579 | 0.60600 | 0.12553 |  |
| *PAPSS2* | 0.00858 | 0.01579 | 1.46580 | 0.17066 | 0.01455 | 0.00921 | 0.26530 | 0.08736 | 0.01296 | 0.01350 | 0.81300 | 0.13706 |  |
| *PKDCC* | 0.01161 | 0.01538 | 0.59110 | 0.08101 | 0.03205 | 0.01927 | 0.35470 | 0.08909 | 0.01099 | 0.01299 | 0.95530 | 0.15433 |  |
| *PLA2G4A* | 0.00424 | 0.00457 | 0.30030 | 0.07545 | 0.09713 | 0.29638 | 0.94320 | 0.24503 | 0.00741 | 0.00954 | 0.98640 | 0.13534 |  |
| *PLXNB1* | 0.00578 | 0.00533 | 0.21750 | 0.04241 | 0.02916 | 0.02608 | 0.61620 | 0.17415 | 0.01848 | 0.01939 | 0.66520 | 0.13598 |  |
| *PTGER4* | 0.00632 | 0.00589 | 0.32340 | 0.06607 | 0.01898 | 0.01544 | 0.65730 | 0.17023 | 0.01335 | 0.01328 | 0.61300 | 0.13520 |  |
| *PTH* | 0.00853 | 0.00985 | 0.29880 | 0.05411 | 0.04726 | 0.03526 | 0.36860 | 0.13967 | 0.03339 | 0.04213 | 0.74840 | 0.13019 |  |
| *PTK2B* | 0.02336 | 0.02824 | 1.02480 | 0.27540 | 0.01602 | 0.01174 | 0.68450 | 0.19707 | 0.00732 | 0.00698 | 0.73590 | 0.12300 |  |
| *PTN* | 0.00373 | 0.00380 | 0.19200 | 0.03684 | 0.01493 | 0.01148 | 0.26620 | 0.09255 | 0.01599 | 0.01619 | 0.41110 | 0.09352 |  |
| *SBDS* | 0.00384 | 0.00524 | 0.91210 | 0.12255 | 0.01093 | 0.00794 | 0.41210 | 0.12835 | 0.00710 | 0.00820 | 0.87830 | 0.19283 |  |
| *SFRP1* | 0.00315 | 0.00301 | 0.47320 | 0.15504 | 0.02403 | 0.02312 | 1.12850 | 0.34750 | 0.00600 | 0.00668 | 0.79280 | 0.14881 |  |
| *SFRP2* | 0.00842 | 0.01355 | 0.78180 | 0.18007 | 0.01321 | 0.01143 | 0.46770 | 0.16156 | 0.00628 | 0.00686 | 0.87070 | 0.18741 |  |
| *SH3PXD2B* | 0.01140 | 0.00988 | 0.23890 | 0.06403 | 0.02745 | 0.01804 | 0.24850 | 0.08950 | 0.01987 | 0.02227 | 1.07070 | 0.17583 |  |
| *SPP2* | 0.02479 | 0.02629 | 0.44840 | 0.08599 | 0.05082 | 0.04856 | 0.22490 | 0.10343 | 0.05700 | 0.05923 | 0.90190 | 0.16448 |  |
| *SRD5A1* | 0.01134 | 0.01051 | 0.17770 | 0.04601 | 0.06481 | 0.05267 | 0.37060 | 0.11412 | 0.04001 | 0.03515 | 0.63600 | 0.12370 |  |
| *SRGN* | 0.02274 | 0.02513 | 0.55400 | 0.12658 | 0.06387 | 0.04781 | 0.28880 | 0.10404 | 0.05488 | 0.06271 | 0.63450 | 0.15241 |  |
| *SULF1* | 0.00465 | 0.00669 | 0.65820 | 0.08278 | 0.00991 | 0.00861 | 0.28350 | 0.09874 | 0.01203 | 0.01292 | 1.00940 | 0.13990 |  |
| *SULF2* | 0.00333 | 0.00378 | 0.38540 | 0.06276 | 0.00924 | 0.00793 | 0.31110 | 0.11497 | 0.01121 | 0.01356 | 1.90560 | 0.26305 |  |
| *SYK* | 0.00533 | 0.01060 | 0.87120 | 0.10892 | 0.02605 | 0.01820 | 0.31800 | 0.11063 | 0.01173 | 0.01427 | 1.18110 | 0.20682 |  |
| *TCF7L2* | 0.00253 | 0.00468 | 0.23940 | 0.03243 | 0.00492 | 0.00281 | 0.11720 | 0.03627 | 0.00871 | 0.00991 | 0.44960 | 0.07475 |  |
| *TFRC* | 0.02526 | 0.02154 | 0.16320 | 0.04533 | 0.11002 | 0.09551 | 0.66090 | 0.22590 | 0.03641 | 0.03707 | 0.55230 | 0.11152 |  |
| *TGFB3* | 0.00103 | 0.00099 | 0.21010 | 0.04939 | 0.01542 | 0.01180 | 0.46050 | 0.14713 | 0.00524 | 0.00602 | 0.51500 | 0.10443 |  |
| *TNFAIP3* | 0.01050 | 0.01028 | 0.34350 | 0.05494 | 0.03341 | 0.02375 | 0.31080 | 0.10328 | 0.01486 | 0.01366 | 0.49310 | 0.12421 |  |
| *TPH1* | 0.00372 | 0.00357 | 0.19130 | 0.03900 | 0.01514 | 0.01148 | 0.32670 | 0.10773 | 0.01277 | 0.01327 | 0.51810 | 0.10355 |  |
| *TPP1* | 0.10336 | 0.09532 | 0.86160 | 0.23031 | 0.04362 | 0.03292 | 0.78050 | 0.23728 | 0.01532 | 0.01576 | 0.39750 | 0.07901 |  |
| *TRAF6* | 0.00521 | 0.00724 | 0.64430 | 0.10823 | 0.02142 | 0.01552 | 0.25350 | 0.09333 | 0.01401 | 0.01347 | 0.43050 | 0.10515 |  |
| *TUFT1* | 0.00725 | 0.00991 | 0.87760 | 0.18696 | 0.04649 | 0.03052 | 0.68420 | 0.19214 | 0.02576 | 0.02928 | 0.75600 | 0.13769 |  |
| *VEGFA* | 0.00177 | 0.00248 | 0.06600 | 0.01168 | 0.02069 | 0.01400 | 0.17940 | 0.05670 | 0.02298 | 0.03318 | 0.54610 | 0.09767 |  |
