# Supplementary material for: Bone-associated gene evolution and the origin of flight in birds
Source: BMC Genomics. 2016 May 18;17:371. doi: 10.1186/s12864-016-2681-7 (PMC4870793; doi:10.1186/s12864-016-2681-7)
Supplement: Additional file 20: Table S14. — Divergence limit estimations. Calibration points retrieved from TimeTree. (DOC 35 kb) [file 12864_2016_2681_MOESM20_ESM.doc]

# Additional file 20: Table S15 - Divergence limit estimations. Calibration points retrieved from TimeTree.

|  |  |  | Upper limit (Myr) | Lower limit (Myr) |
| --- | --- | --- | --- | --- |
| Mammals | Loxodonta | Echinops | 93 | 78 |
|  | Homo | Pan | 13 | 2.7 |
|  | Cavia | Mus | 115 | 44 |
|  | Pongo | Otolemur | 98 | 40 |
|  | Macaca | Tarsius | 91 | 40 |
| Birds | Apaloderma | Aptenodytes | 103 | 66 |
|  | Calypte | Apaloderma | 106 | 66 |
|  | Calypte | Chaetura | 80 | 45 |
|  | Chlamydotis | Mesitornis | 99 | 74 |
|  | Cuculus | Corvus | 119 | 70 |
|  | Geospiza | Gallus | 135 | 86.5 |
|  | Mesitornis | Fulmarus | 86 | 82 |
|  | Phaethon | Egretta | 82 | 63 |
|  | Phalacrocorax | Cariama | 91.5 | 64 |
|  | Eurypyga | Nestor | 112 | 87 |
|  | Leptosomus | Aptenodytes | 114 | 66 |
|  | Leptosomus | Buceros | 114 | 67.6 |
