# Supplementary material for: Bone-associated gene evolution and the origin of flight in birds
Source: BMC Genomics. 2016 May 18;17:371. doi: 10.1186/s12864-016-2681-7 (PMC4870793; doi:10.1186/s12864-016-2681-7)
Supplement: Additional file 21: Table S15. — Body mass in birds and mammals. (DOCX 46 kb) [file 12864_2016_2681_MOESM21_ESM.docx]

# Additional file 21: Table S16 - Body mass in birds and mammals.

|  |  | Minimum Weight (g) | Maximum Weight (g) | Average Weight (g) | References |
| --- | --- | --- | --- | --- | --- |
| Mammals | *Ailuropoda melanoleuca* | 80000 | 125000 | 102500 | [[1](#_ENREF_1)] |
|  | *Bos taurus* | 147000 | 1363000 | 755000 | [[1](#_ENREF_1)] |
|  | *Callithrix jacchus* | 300 | 360 | 330 | [[1](#_ENREF_1)] |
|  | *Cannis familiaris* | 1000 | 70000 | 35500 | [[1](#_ENREF_1)] |
|  | *Cavia porcellus* | 700 | 1100 | 900 | [[1](#_ENREF_1)] |
|  | *Choloepus hoffmanni* | 4000 | 8000 | 6000 | [[1](#_ENREF_1)] |
|  | *Dasypus novemcinctus* | 3600 | 7700 | 5650 | [[1](#_ENREF_1)] |
|  | *Dipodomys ordii* | 55 | 96 | 75.5 | [[1](#_ENREF_1)] |
|  | *Echinops telfairi* | 175 | 279 | 227 | [[2](#_ENREF_2)] |
|  | *Equus caballus* | 227000 | 900000 | 563500 | [[1](#_ENREF_1)] |
|  | *Erinaceus europaeus* | 800 | 1200 | 1000 | [[1](#_ENREF_1)] |
|  | *Felis catus* | 4100 | 5400 | 5650 | [[1](#_ENREF_1)] |
|  | *Gorilla gorilla* | 72000 | 181000 | 126500 | [[3](#_ENREF_3)] |
|  | *Homo sapiens* | 54000 | 83000 | 68500 | [[4](#_ENREF_4)] |
|  | *Loxodonta africana* | 3600000 | 6000000 | 4800000 | [[1](#_ENREF_1)] |
|  | *Macaca mulatta* | 4000 | 12000 | 8000 | [[1](#_ENREF_1)] |
|  | *Macropus eugenii* | 400 | 9100 | 4750 | [[1](#_ENREF_1)] |
|  | *Microcebus murinus* | 40 | 70 | 55 | [[2](#_ENREF_2)] |
|  | *Monodelphis domestica* | 90 | 155 | 122.5 | [[1](#_ENREF_1)] |
|  | *Mus musculus* | 12 | 30 | 21 | [[1](#_ENREF_1)] |
|  | *Myotis lucifugus* | 5 | 14 | 9.5 | [[1](#_ENREF_1)] |
|  | *Nomascus leucogenys* | 5600 | 5800 | 5700 | [[5](#_ENREF_5)] |
|  | *Ochotona princeps* | 121 | 176 | 148.5 | [[1](#_ENREF_1)] |
|  | *Ornithorhynchus anatinus* | 800 | 2500 | 1650 | [[1](#_ENREF_1)] |
|  | *Oryctolagus cuniculus* | 1500 | 2500 | 2000 | [[1](#_ENREF_1)] |
|  | *Otolemur garnettii* | 721 | 822 | 771.5 | [[1](#_ENREF_1)] |
|  | *Pan troglodytes* | 26000 | 70000 | 48000 | [[1](#_ENREF_1)] |
|  | *Pongo abelii* | 30000 | 90000 | 60000 | [[1](#_ENREF_1)] |
|  | *Procavia capensis* | 1800 | 5400 | 3600 | [[6](#_ENREF_6)] |
|  | *Pteropus vampyrus* | 600 | 1100 | 850 | [[1](#_ENREF_1)] |
|  | *Rattus norvegicus* | 140 | 500 | 320 | [[1](#_ENREF_1)] |
|  | *Sarcophilus harrisii* | 4000 | 12000 | 8000 | [[1](#_ENREF_1)] |
|  | *Sorex araneus* | 5 | 14 | 9.5 | [[1](#_ENREF_1)] |
|  | *Ictidomys tridecemlineatus* | 100 | 220 | 160 | [[7](#_ENREF_7)] |
|  | *Sus scrofa* | 66000 | 272000 | 169000 | [[1](#_ENREF_1)] |
|  | *Tarsius syrichta* | 85 | 165 | 125 | [[1](#_ENREF_1)] |
|  | *Tupaia belangeri* | 50 | 270 | 160 | [[1](#_ENREF_1)] |
|  | *Tursiops truncatus* | 260000 | 500000 | 380000 | [[1](#_ENREF_1)] |
|  | *Vicugna pacos* | 55000 | 65000 | 60000 | [[1](#_ENREF_1)] |
| Birds | *Acanthisitta chloris* | 6 | 7 | 7 | [[8](#_ENREF_8)] |
|  | *Anas platyrhynchos* | 720 | 1580 | 1150 | [[9](#_ENREF_9)] |
|  | *Apaloderma vittatum* | 46 | 55 | 50 | [[8](#_ENREF_8)] |
|  | *Aptenodytes forsteri* | 22000 | 37000 | 29500 | [[1](#_ENREF_1)] |
|  | *Balearica regulorum* | 3000 | 4000 | 3500 | [[1](#_ENREF_1)] |
|  | *Buceros rhinoceros* | 2000 | 3000 | 2500 | [[8](#_ENREF_8)] |
|  | *Calypte anna* | 4 | 5 | 4 | [[1](#_ENREF_1)] |
|  | *Cariama cristata* | 2500 | 3000 | 2750 | [[10](#_ENREF_10)] |
|  | *Cathartes aura* | 850 | 2000 | 1425 | [[1](#_ENREF_1)] |
|  | *Chaetura pelagica* | 17 | 30 | 24 | [[9](#_ENREF_9)] |
|  | *Charadrius vociferus* | 75 | 128 | 102 | [[9](#_ENREF_9)] |
|  | *Chlamydotis undulata* | 1100 | 3100 | 2100 | [[8](#_ENREF_8)] |
|  | *Columba livia* | 265 | 380 | 323 | [[8](#_ENREF_8)] |
|  | *Colius striatus* | 36 | 80 | 58 | [[8](#_ENREF_8)] |
|  | *Corvus brachyrhynchos* | 316 | 620 | 468 | [[9](#_ENREF_9)] |
|  | *Cuculus canorus* | 54 | 60 | 57 | [[11](#_ENREF_11)] |
|  | *Egretta garzetta* | 350 | 550 | 450 | [[12](#_ENREF_12)] |
|  | *Eurypyga helias* | 189 | 295 | 242 | [[8](#_ENREF_8)] |
|  | *Falco peregrinus* | 530 | 1600 | 1065 | [[9](#_ENREF_9)] |
|  | *Fulmarus glacialis* | 450 | 1000 | 725 | [[1](#_ENREF_1)] |
|  | *Gallus gallus* | 485 | 1450 | 968 | [[8](#_ENREF_8)] |
|  | *Gavia stellata* | 1000 | 2700 | 1850 | [[9](#_ENREF_9)] |
|  | *Geospiza fortis* | 18 | 32 | 25 | [[8](#_ENREF_8)] |
|  | *Haliaeetus leucocephalus* | 3000 | 6300 | 4650 | [[9](#_ENREF_9)] |
|  | *Leptosomus discolor* | 160 | 301 | 231 | [[8](#_ENREF_8)] |
|  | *Manacus vitellinus* | 17 | 19 | 18 | [[8](#_ENREF_8)] |
|  | *Meleagris gallopavo* | 3000 | 11000 | 7000 | [[1](#_ENREF_1)] |
|  | *Melopsittacus undulatus* | 26 | 29 | 28 | [[8](#_ENREF_8)] |
|  | *Merops nubicus* | 34 | 59 | 47 | [[8](#_ENREF_8)] |
|  | *Mesitornis unicolor* | 93 | 113 | 103 | [[13](#_ENREF_13)] |
|  | *Nestor notabilis* | 800 | 1000 | 900 | [[14](#_ENREF_14)] |
|  | *Nipponia nippon* | 950 | 1600 | 1275 | [[13](#_ENREF_13)] |
|  | *Ophisthocomus hoazin* | 695 | 900 | 798 | [[8](#_ENREF_8)] |
|  | *Phalacrocorax carbo* | 2600 | 3700 | 3150 | [[9](#_ENREF_9)] |
|  | *Phaethon lepturus* | 220 | 410 | 315 | [[8](#_ENREF_8)] |
|  | *Phoenicopterus ruber* | 2100 | 4100 | 3100 | [[8](#_ENREF_8)] |
|  | *Picoides pubescens* | 21 | 28 | 25 | [[9](#_ENREF_9)] |
|  | *Podiceps cristatus* | 800 | 1490 | 1145 | [[1](#_ENREF_1)] |
|  | *Pterocles gutturalis* | 285 | 400 | 343 | [[8](#_ENREF_8)] |
|  | *Pygoscelis adeliae* | 3620 | 4990 | 4305 | [[1](#_ENREF_1)] |
|  | *Struthio camelus* | 90000 | 130000 | 110000 | [[1](#_ENREF_1)] |
|  | *Taeniopygia guttata* | 10 | 14 | 12 | [[1](#_ENREF_1)] |
|  | *Tauraco erythrolophus* | 210 | 325 | 268 | [[8](#_ENREF_8)] |
|  | *Tinamus guttatus* | 623 | 800 | 712 | [[8](#_ENREF_8)] |
|  | *Tyto alba* | 430 | 620 | 525 | [[1](#_ENREF_1)] |

1. Myers, P., et al., *The Animal Diversity Web (online).* in *Accessed at* [*http://animaldiversity.org*](http://animaldiversity.org)*.* 2014.

2. Garbutt, N., *Mammals of Madagascar: A complete guide*. 2007: Yale University Press.

3. Cawthon Lang, K. *Primate Factsheets: Gorilla (Gorilla) Taxonomy, Morphology, & Ecology*. 2005 [cited 2015 May].

4. Roberts, A., *Evolution The Human Story*. 2011: Dorling Kindersley Ltd.

5. Cawthon Lang, K. *Primate Factsheets: White-cheeked gibbon (Nomascus leucogenys) Taxonomy, Morphology, & Ecology* 2010 [cited 2015 May].

6. Burton, R., *The Marshall Cavendish International Wildlife Encyclopedia: WIL-ZOR*. Vol. 24. 1994: Marshall Cavendish.

7. Streubel, D.P. and J.P. Fitzgerald, *Spermophilus tridecemlineatus.* Mammalian Species, 1978: p. 1-5.

8. del Hoyo, J., et al. *Handbook of the Birds of the World Alive. Lynx Edicions, Barcelona*. 2015 [cited (retrieved from <http://www.hbw.com/> on 15 January 2015).

9. *Lab of Ornithology, Cornell All about birds 2015*. [cited 2015 15 January]; Available from: <http://www.allaboutbirds.org/>.

10. Hallager, S. *Avian Scientific Advisory Group*. 2014 [cited 2015 15 Janurary]; Available from: <http://aviansag.org/Fact_Sheets/Gruiformes/Red-legged_Seriema.pdf>.

11. Mullarney, K., et al., *Collins bird guide*. 1999: HarperCollins.

12. Seabrook-Davison, M.N.H. *Little egret. In Miskelly, C.M. (ed.) New Zealand Birds Online.* 2013 [cited 2015 25 January]; Available from: [www.nzbirdsonline.org.nz](http://www.nzbirdsonline.org.nz).

13. Grzimek, B., et al., *Grzimek's animal life encyclopedia*. 2004: Gale Farmington Hills, Michigan.

14. Dunning Jr, J.B., *CRC handbook of avian body masses*. 1992: CRC press.
